# Methane Oxidation to Methanol

**DOI:** 10.1021/acs.chemrev.2c00439

**Published:** 2022-12-02

**Authors:** Nicholas F. Dummer, David J. Willock, Qian He, Mark J. Howard, Richard J. Lewis, Guodong Qi, Stuart H. Taylor, Jun Xu, Don Bethell, Christopher J. Kiely, Graham J. Hutchings

**Affiliations:** † Max Planck−Cardiff Centre on the Fundamentals of Heterogeneous Catalysis FUNCAT, Cardiff Catalysis Institute, School of Chemistry, Cardiff University, Main Building, Park Place, CardiffCF10 3AT, United Kingdom; ‡ Department of Materials Science and Engineering, National University of Singapore, Singapore117575, Singapore; § National Center for Magnetic Resonance in Wuhan, State Key Laboratory of Magnetic Resonance and Atomic and Molecular Physics, Innovation Academy for Precision Measurement Science and Technology, Chinese Academy of Sciences, Wuhan430071, P. R. China; ∥ University of Chinese Academy of Sciences, Beijing100049, P. R. China; ⊥ Department of Chemistry, University of Liverpool, Crown Street, LiverpoolL69 7ZD, United Kingdom; # Department of Materials Science and Engineering, Lehigh University, 5 East Packer Avenue, Bethlehem, Pennsylvania18015, United States

## Abstract

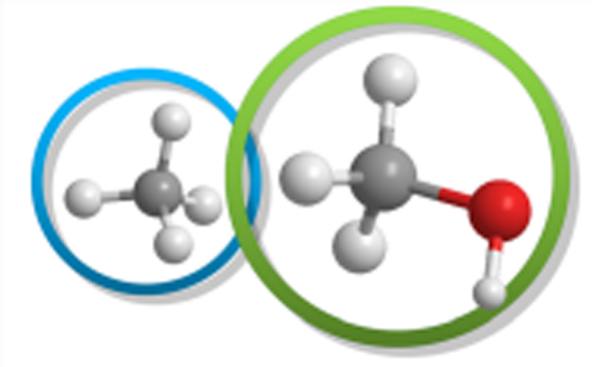

The direct transformation of methane to methanol remains a significant challenge for operation at a larger scale. Central to this challenge is the low reactivity of methane at conditions that can facilitate product recovery. This review discusses the issue through examination of several promising routes to methanol and an evaluation of performance targets that are required to develop the process at scale. We explore the methods currently used, the emergence of active heterogeneous catalysts and their design and reaction mechanisms and provide a critical perspective on future operation. Initial experiments are discussed where identification of gas phase radical chemistry limited further development by this approach. Subsequently, a new class of catalytic materials based on natural systems such as iron or copper containing zeolites were explored at milder conditions. The key issues of these technologies are low methane conversion and often significant overoxidation of products. Despite this, interest remains high in this reaction and the wider appeal of an effective route to key products from C–H activation, particularly with the need to transition to net carbon zero with new routes from renewable methane sources is exciting.

## 1 Introduction and Context

### 1.1 Is There Still a Need for New Research for the Selective Oxidation of Methane to Methanol?

The conversion of methane, the main component of natural gas has been viewed as a grand challenge for catalysis chemists for over a century. Every decade, a new approach is found that seems to herald a new route to effective catalysis to meet this challenge. The result is that there has been a wide range of publications on the topic, but as yet there has been no large-scale application of this research. It is therefore pertinent to ask whether or not this grand challenge still exists today?

Although the world’s natural gas resources remain abundant,^1^ in the context of climate change and aspirations for net zero carbon emissions, it seems appropriate to re-examine the purpose and motivation for research aimed at new ways to convert methane to higher value fuels and chemicals. The 20th century concept of large scale “stranded gas” resources needing new, more cost-effective routes to market is out of date. The progressive increase in scale and efficiency of conventional methanol processes,^2^ combined with the development of a global liquefied natural gas (LNG) industry,^3^ have already provided viable solutions for exploiting large scale maritime gas (i.e., gas with ready access to the sea). As arguably the least damaging fossil fuel, demand for natural gas has a longer projected lifetime than for coal and oil,^1,4^ but with reserves-to-production ratios stable at ∼50 years,^1^ this can probably be met from known, exploitable resources. If any major natural gas resources remain genuinely “stranded”, perhaps they should be left in place.

The global warming potential (GWP) of methane is estimated to be 28–36 over 100 years (GWP of CO_2_ is defined as 1), and the emission of methane from human activity is the second most important contributor to climate change after CO_2_.^5^ Oil and natural gas facilities account for approximately 24% of all anthropogenic methane emissions,^6^ representing a major issue that must be addressed by the industry without delay. There are established technical solutions for >75% for this problem that should be implemented urgently (with net costs estimated at less than the value of methane recovered^7^). An estimated 142 BCM of natural gas was flared in 2020, mostly associated with oil production.^8^ This is equivalent to ∼3.5% of all natural gas production,^9^ and there is growing pressure, with emerging commitment, to reduce this dramatically by 2030.^8^ In addition to the implementation of gas gathering pipeline networks (for export, reinjection, or local power generation), small-scale chemical conversion to liquid fuels or chemicals offers a potential solution, alongside other options such as compressed natural gas (CNG), mini-LNG^10^ or creating portable, local power demand (e.g., mobile high intensity computing^11^). Hopefully, most of today’s flaring of associated gas will be eliminated by 2030 using existing or near commercial technologies (the IEA’s Net Zero by 2050 scenario proposes 90% reduction by 2030^8^), probably too soon for new chemistry and catalysis to be widely implemented. Therefore, the opportunity for novel chemical processes to valorize associated gas lies mainly with future oil developments, not yet in the detailed planning phase. This may be a very limited opportunity set if demand for oil declines on a trajectory consistent with 1.5 °C global warming scenarios which require few, if any, new oil developments.^12^ To be competitive with those alternatives outlined above, and meet ever more stringent environmental expectations, such chemistries will need to be highly efficient, capturing most of the carbon, as well as being cost-effective. In other words, an inefficient conversion of associated gas, even if low-cost, may be seen as only a partial reduction in flaring and emissions.

The world will continue to need methanol (and its derivatives), which is currently almost entirely derived from natural gas (∼65%) and coal (∼35%, mainly in China).^13^ Demand for methanol reached 106 million tonnes in 2021,^14^ almost doubling over the previous decade, and is expected to continue to grow strongly.^13^ More than 60% of current demand is as chemical feedstock, mainly for the manufacture of olefins (32%), formaldehyde (23%), and acetic acid (8%). Methanol is also widely used in transport fuels via methyl *tert*-butyl ether (MTBE) (11%), biodiesel (3%) and by direct blending or substitution in the gasoline pool (11%), the latter growing strongly.^14^ Low-cost natural gas will remain an attractive feedstock for methanol, especially if strong growth in shale gas production returns in the U.S. following its moderation in 2019–2020. However, there is growing interest in renewable methanol which may increasingly drive and compete for market growth in the coming decades^13^ and potentially displacing a large proportion of fossil-based supply (perhaps as much as 50% by 2050^15^). Biomethane is a legitimate feedstock for renewable methanol; indeed, this is already being used in Europe as a cofeed with natural gas to otherwise conventional methanol production,^16,17^ and Topsoe is operating a demonstration plant for biogas to methanol using compact, electrified reforming.^18,19^

Nevertheless, in many cases, other biogenic feedstocks will be more cost-effective, and these routes are also emerging (e.g., Enerkem municipal solid waste (MSW) to methanol process^20^). Whatever the feedstock, the known gasification/syngas-based routes offer the prospect of ready integration with renewable electricity and green hydrogen to boost carbon utilization, with approaching 100% utilization being feasible, thus raising the performance bar for any new, direct methane to methanol routes. Ultimately, a new competitor may emerge for methanol production based on CO_2_ and green hydrogen, initially using byproduct CO_2_ of various kinds, but perhaps with CO_2_ from direct air capture in due course.^13^ A pioneering commercial methanol plant hydrogenating CO_2_ recovered from flue gas with green hydrogen has been operating in Iceland since 2012.^21^

Overall, the need for a new, direct conversion of methane to methanol (or other derivatives) is less clear than it was in the 1980s and 1990s, when major efforts in this area first got underway. There are efficient alternative solutions to many of the perceived needs, and environmental expectations are much higher. What does seem clear is that high energy and carbon efficiency will be required for new catalysis to be of practical interest. For this reason, we begin this review with the performance targets for direct oxidative conversion of methane (section 1.2) before moving through the approaches that have been put forward in the literature. The review will place emphasis on mechanistic insights that could lead to further developments to meet these targets. Section 2.1 then scopes out the use of high temperature homogeneous reactions that occur when methane reacts with oxygen in the absence of a catalyst. This puts in place many of the radical based elementary steps that occur in catalytic systems using gas phase reagents, radicals that become a recurring theme through the review. The possibility of alternative low temperature catalyzed reactions in a liquid solvent have been inspired by naturally occurring enzymes, and so section 2.2 briefly introduces these systems and outlines the performance that has been achieved under laboratory conditions.

### 1.2 Performance Targets

Modern, conventional natural gas to methanol processes in favorable locations have typical thermal efficiencies of 66–68% (lower heating value, LHV),^22,23^ with corresponding carbon utilization between 4% and 8% higher. Future improvements in efficiency are possible, perhaps by up to 5%,^23^ and introducing low carbon energy and/or green hydrogen could improve the effective carbon utilization still further. Therefore, whatever the context, a carbon utilization of 75% seems a *minimum* performance hurdle for any new, direct conversion process aimed at competing in conventional markets, especially if CO_2_ emissions start to attract significant penalties. It seems unlikely that lower capital costs can significantly soften this target in a future, low emissions world, and even higher carbon utilizations may eventually be required.

Technical and economic evaluations of direct methane to methanol concepts carried out in the late 1980s and early 1990s were summarized by Foulds and Gray in 1995.^24^ These studies provided a reasonably consistent view that selectivity to methanol is more important than once-through conversion, although ∼5% is a likely minimum, with selectivities of ∼80% at 5% once-through conversion or ∼70% at 10% conversion being required for approximate parity with conventional processes. A contemporaneous evaluation involving a reputable engineering contractor^25^ suggested an even higher requirement of 95% selectivity at 10% conversion for a competitive process. An industrial analysis based on heat transfer cost indices concluded that a direct process with 2.5% methane conversion and 80% methanol selectivity has capital costs approximately 15% higher than conventional routes, although this is subject to considerable uncertainty.^26^ More recently, Baliban et al.^27^ included direct methane to methanol cases in a global optimization study of natural gas to liquid fuel processes. Here, a direct oxidation case with 13% methane conversion and 63% methanol selectivity, followed by methanol-to-gasoline (MTG) conversion, gave ∼15% higher final gasoline product costs than routes based on steam reforming, even at quite small scale (1 kbd). By inspection, a higher selectivity of around 75% would bring costs to approximate parity, which remains consistent with the earlier Foulds and Gray view.^24^ It is worth a note of caution at this point that some commercial and patent literature in this area does not specify wt % or mol % when quoting oxygenate yields, which can be misleading.

These are formidable performance targets, and it is important not to deny the opportunity for innovative process engineering to overcome some of the perceived downsides of conceptual, direct routes. Nevertheless, it seems clear that at least a high selectivity of ∼75% at meaningful once-through conversions (i.e., ≥5%) will be needed to be potentially competitive with established approaches aimed at conventional methanol markets. Realistically, something beyond this is likely to be required to provide a compelling incentive for major new catalytic process development. This surpasses the performances reliably reported to date using molecular oxygen as the oxidant and implies that a successful system will require features that limit the further oxidation of the methanol product, which is normally regarded as much more reactive than the methane feedstock.

The selective partial oxidation of methane to methanol with molecular oxygen is, of course, strongly exothermic (CH_4_ + ^1^/_2_O_2_ → CH_3_OH, Δ*H* = −126 kJ mol^–1^ standard change at 298 K), somewhat more so than methanol from syngas (CO + 2H_2_ → CH_3_OH, Δ*H* = −90.5 kJ mol^–1^) but significantly less exothermic than Fischer–Tropsch (CO + 2H_2_ → −(CH_2_)– + H_2_O, Δ*H* = −150 to −160 kJ mol^–1^ for typical products). However, any nonselective generation of CO or CO_2_ greatly increases the heat release; for example, even 20% selectivity to CO_2_ renders partial oxidation of methane to methanol ∼70% more exothermic than Fischer–Tropsch. This reinforces the desire for high selectivity to manage heat release in practical reaction systems at productivities comparable to current industrial processes such as methanol or Fischer–Tropsch. Typical reactor productivities for these industrial processes are in the range 5–30 carbon moles L^–1^ h^–1^,^28,29^ which suggests productivity should ideally be in the moles L^–1^ h^–1^ range. Operating pressures of at least several bar are also highly desirable (preferably >10 bar), with reaction temperatures at or above 150 °C to facilitate heat recovery by raising high pressure steam.

It is also important to consider the selectivity of oxygen utilization for systems using molecular oxygen. For example, if only one of the oxygen atoms from the O_2_ molecule is incorporated into the methanol product (as is the case for the well-known methane monooxygenase system,^30^section 2.2), and there is no other sacrificial reductant, the maximum theoretical methanol selectivity is 80% (5CH_4_ + 4O_2_ → 4CH_3_OH + CO_2_ + 2H_2_O). Strategies to incorporate both oxygen atoms into the methanol product are required to exceed this limit. In contrast, selective conversion of methane to formaldehyde or acetic acid, both major industrial methanol derivatives, requires only 50% selectivity based on oxygen.

## 2 Background Chemistry

### 2.1 High Pressure, Moderate Temperature

The gas phase partial oxidation of methane to methanol and formaldehyde at high pressure (20–100 bar) and moderate temperature (350–500 °C) has been known for over a century, with significant experimental effort during the 1980s and 1990s, typically with 2–10 vol % oxygen and a few seconds residence time in the reactor. This area has been thoroughly reviewed by other authors,^31−34^ and we are not aware of significant new experimental work since then. This section will therefore give only a brief summary in order to provide context for catalytic studies; first, as a performance benchmark and, second, to introduce the gas phase radical chemistry that occurs under these conditions.

At temperatures below about 600 °C, and in the presence of significant oxygen partial pressures, the equilibrium:
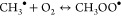
lies strongly to the right,^31^ and the chemistry of methylperoxy radicals is therefore of central importance in this system. The two self-reactions:
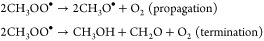
have broadly competitive rates in the relevant temperature range,^35,36^ with the reaction sequence:
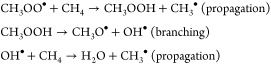
contributing to chain branching. The methoxy radicals form partial oxidation products via competing reactions:
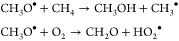
with the HO_2_^•^ produced being able to participate in radical recombination reactions with itself and methylperoxy, as well as activate methane:
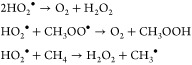
Substantially more stable than methyl hydroperoxide,^37^ hydrogen peroxide may make a small contribution to chain branching or, more likely, be lost at reactor walls or react further in the liquid product. Formaldehyde is more reactive than methanol and oxidizes to CO via HCO^•^ in reactions with oxygen or other radicals^36^ or possibly decomposes to CO and H_2_.^34^ Final conversion of CO to CO_2_ is generally underpredicted by kinetic models compared to experiment, although CO still usually dominates, supporting the proposal that this is mainly a heterogeneous reaction occurring at reactor walls.^31,33^ Naturally, as methane conversion increases, other reactions of the methanol and formaldehyde products make larger contributions to the mechanism, and the description above becomes a highly simplified view.

**Figure 1:**
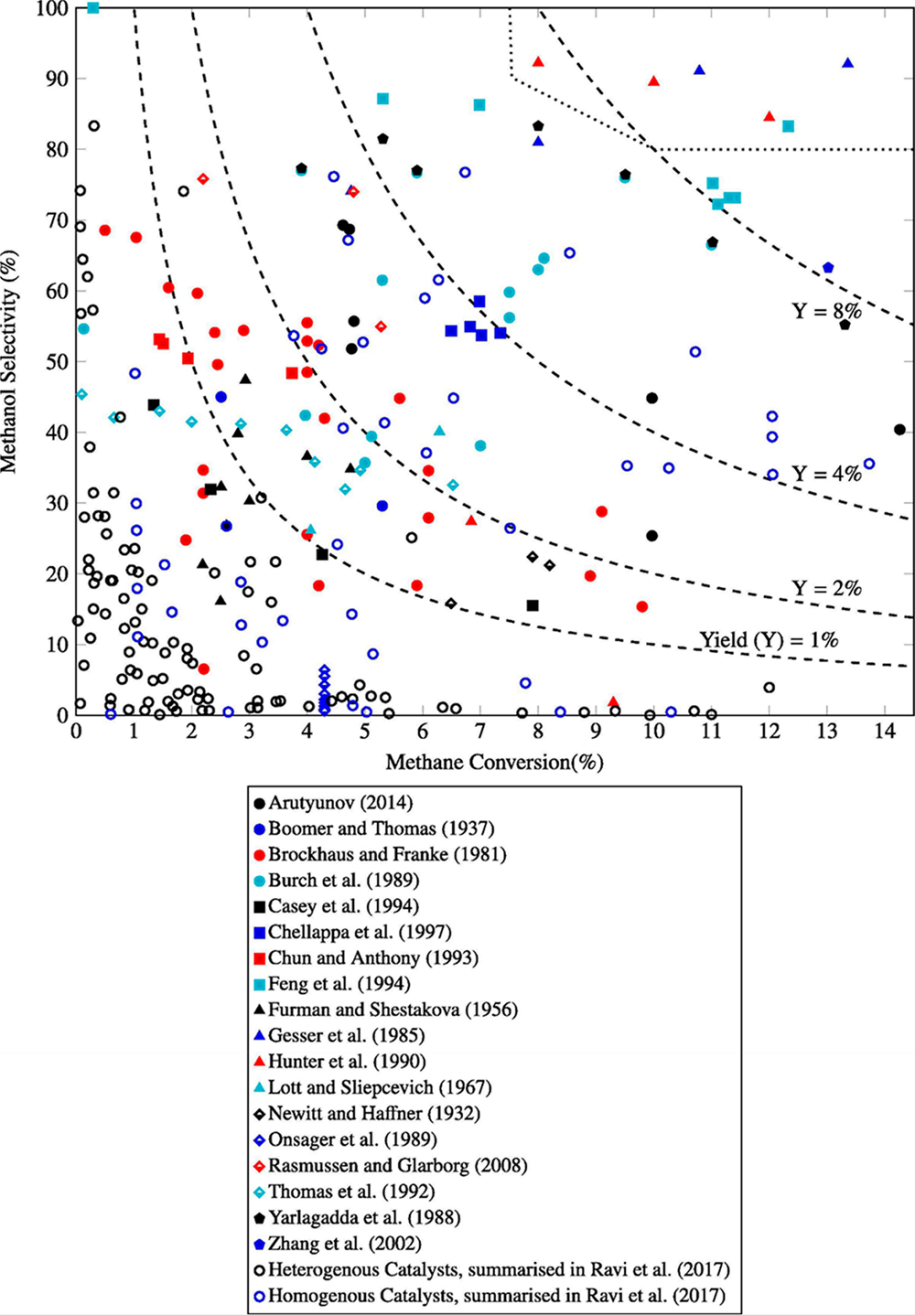
Methanol selectivity versus methane conversion for gas phase reactions. Reproduced with permission from ref (38). Copyright 2021 Elsevier.

The substantial scatter in experimental results is illustrated in Turan et al.’s recent comparison of historical data with kinetic models from the literature (Figure 1).^38^ Not all of the experimental data has been confirmed by other workers, and a methanol selectivity of around 40–60% at ∼5% methane conversion with limiting once-through methanol yield of ∼2.5 mol % in a premixed system seems to be reasonably reproducible.^31^ There may be opportunities to increase once-through methane conversion and methanol yield, although not selectivity, by multiple stages of oxygen addition^33^ or separate addition of oxygen into an intensively back-mixed reaction chamber,^39^ perhaps up to around 10% conversion. Some of the variation in experimental results is due to reactor design and materials, with “inert” materials such as quartz and Pyrex generally giving better methanol selectivities than stainless steel,^34^ especially at lower pressures. Methanol yields as high as 7–8 mol % (13% methane conversion, 60% methanol selectivity) have been claimed in quartz reactors carefully designed to eliminate all gas/metal contact,^40^ although this seems to be an outlier from the main body of results.

Addition of higher hydrocarbon components representative of natural gas, and of other vapor phase “sensitizers”, has been shown to reduce reaction temperature, although the impact on methanol selectivity is modest.^41,42^ There may be opportunities to modify the gas phase homogeneous chemistry by addition of a heterogeneous catalyst,^34^ but this would need to compete with the high radical flux from the homogeneous reactions and a beneficial effect on methanol selectivity is likely to be very difficult to achieve in his way.^33^ However, it is important that the possibility of a homogeneous gas phase contribution is considered during work aimed at heterogeneous catalysis under conditions of high pressure and temperatures in the 300–600 °C range.

In summary, although somewhat short of commercial performance targets for widespread application, the gas phase homogeneous system is capable of relatively high methanol selectivity at low conversion and outperforms most of the known heterogeneous catalytic systems using molecular oxygen as oxidant.^43^ Indeed, this gas phase chemistry forms the basis for a new, small-scale methane to methanol process currently being promoted for certain niche applications.^44^ Recent design studies also illustrate a potential application for remote locations where low yields of methanol are required for local uses, such as methane hydrate suppression, with the bulk of the product remaining as gaseous fuel.^45^

#### 2.1.1 Nonthermal Plasmas

Commercial water electrolysis currently requires an electrical energy input of 0.10–0.16 kWh mol^–1^ of hydrogen^46,47^ which could be used to hydrogenate carbon dioxide to give e-methanol (implying 0.3–0.48 kWh mol^–1^ of e-methanol). Electrically heated reforming could have a much lower power demand for production of syngas and hydrogen, for example, 0.025 kWh mol^–1^ hydrogen equivalent for a scaled-up inductively heated reformer.^48^ This translates to a power demand for methanol of around 0.08 kWh mol^–1^. An alternative use of electrical power is to support reactions in gas phase CH_4_/O_2_ or CH_4_/CO_2_ mixtures in a nonthermal plasma (NTP) at near ambient conditions, either with or without the presence of a heterogeneous catalyst, as has been reviewed recently by Li et al.^49^ and Nozaki et al.^50^ Modeling of the plasma chemistry in a dielectric barrier discharge (DBD), which is the practical configuration usually employed, suggests that electron impact dissociation of CH_4_ to CH_3_^•^ radicals and H atoms is the primary driver for reaction. In the presence of molecular oxygen, CH_3_^•^ forms CH_3_OO^•^, followed by a cascade of reactions to oxygenates, including methanol, and carbon oxides; electron impact dissociation of O_2_ to atomic oxygen species also makes a secondary contribution. In the presence of CO_2_, CH_3_^•^ radicals mainly recombine to form C_2_^+^ species, with CH_2_^••^ from electron dissociation of CH_4_ also playing a significant role in the formation of formaldehyde and CO.^51^

Even for empty reactors, DBD systems have narrow annular reaction volumes with high surface-to-volume ratios and surface/reactor wall effects are likely to be significant in all cases (especially at ambient pressure). Indeed, an impressive 27.5% methanol yield (36.2% selectivity at 76% CH_4_ conversion) recently reported in an empty reactor is partly attributed to the oxidized copper electrode surface, as well as optimization of other reaction and discharge parameters.^52^ The electrical power input in this case was equivalent to ∼0.95 kWh mol^–1^ methanol. A number of studies have reported increased methane conversions when reactor volumes are filled with solid “catalysts”, and although these increases are generally modest, they represent a large increase in reaction rate given the reactor volume occluded by the catalysts and correspondingly large reductions in residence times. For example, Chawdhury and co-workers have recently shown that adding an Fe/γ-Al_2_O_3_ material into the reactor volume increases methane conversion from 7% to 13%, with a corresponding improvement of methanol selectivity from ∼20% to ∼36% (with total oxygenates of ∼71%).^53^ At the same time, energy efficiency improves from the equivalent of 1.85 kWh mol^–1^ of methanol to 0.58 kWh mol^–1^ methanol, which is not far above the water electrolysis based e-methanol figures quoted previously and possibly the most important effect of the catalyst. Similarly, Yi and co-workers report an increase in methane conversion from ∼4% to ∼6% with associated increase in methanol selectivity from 42% to 50% (76% to 81% for total oxygenates) on adding a NiO/γ-Al_2_O_3_ material.^54^ Energy efficiency again improves very substantially from 1.3 kWh mol^–1^ methanol to 0.71 kWh mol^–1^ on addition of the catalyst. There may also be some limited additional value available from the coproducts (formic acid, formaldehyde, C_2_ hydrocarbons, CO, and H_2_).

Mixtures of methane and CO_2_ tend to produce more higher hydrocarbons and a more complex mixture of liquid C_1_ and C_2_ oxygenates^51,55^ and appear to require even more electrical power, but the potential application to biogas (in particular) remains intriguing. Clearly, for both CH_4_/O_2_ and CH_4_/CO_2_, there is a very complex interaction between the gaseous “plasma phase” and reactor/electrode/catalyst surfaces, including adsorbed species, where bulk catalysts and surfaces affect the physical nature of the discharge and the discharge affects the chemistry at the surfaces. This will require highly probing experimental techniques supported by modeling to deconvolute.^49^ Ambient pressure is generally not a process advantage, and mixed oxygenate products will require separation, but near ambient temperature may enable in situ condensation of products.^49^ However, electrical power requirements will need to improve considerably in order to compete with alternative routes to “e-methanol”, particularly those based on electrified reforming.

### 2.2 Methane Oxidation Using Enzymes

Methanotrophic bacteria are considered to have existed on Earth for about 2 billion years. They utilize methane as their sole energy source. Methanotrophs use a class of enzymes, methane monooxygenases (MMOs), to oxidize methane to methanol as the first stage of methane metabolism. There are two types of MMO, namely (i) a soluble form (sMMO) that has a diiron active center, and (ii) a membrane bound particulate form (pMMO) which has a Cu active site.^56,57^ These enzymes have been studied extensively in recent years,^58^ with the most studied possibly being the pMMO used by the bacterium *Methylococcus capsulatus* (Bath),^59^ which was first isolated in the Roman baths in Bath, UK. Although pMMO is certainly the most predominant form of MMOs found in natural methaneotrophs, it is very difficult to isolate in a pure form,^59^ and hence many studies have been on sMMOs as these are easier to work with. This section of the review will briefly consider the active site of the iron-based sMMO, the mechanism of methane oxidation, and the rates of oxidation with and without cofactors, so that well-informed comparisons can be made with the chemocatalysts which are the focus of this review. Additionally, we will briefly consider pMMO and in particular recent work by Koo et al., demonstrating an effective strategy to reconstitute pMMO in nanodiscs with lipids from the native organism,^60^ which has been a significant challenge.

sMMO is a multicomponent enzyme which comprises three key components: a hydroxylase (MMOH) which converts methane into methanol, a reductase (MMOR) that activates the oxygen and transfers this to the active center of the hydroxylase, and a regulatory protein (MMOB) that controls the admission of the methane to the active site of the hydroxylase. The active site is buried deep within the structure, and the methane and oxygen are transported to the active site through a hydrophobic cavity that runs through the center. Methanol once formed being hydrophilic is readily ejected from the enzyme, preventing overoxidation.

**Figure 2:**
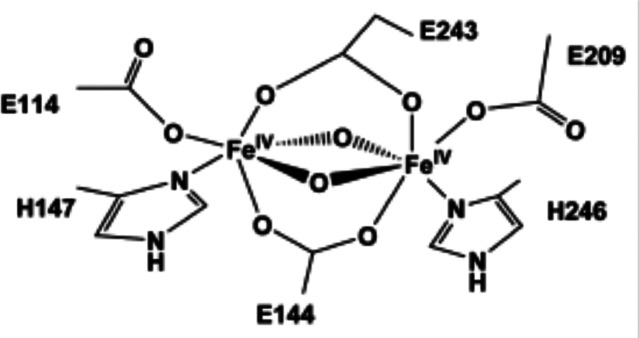
Diamond-core structure of compound Q proposed in sMMO with two Fe(IV) bridged by oxygen atoms. The numbers denote amino acids in the side chains: H, histidine; E, glutamate (Figure 6). Reproduced with permission from ref (58). Copyright 2017 American Chemical Society. Adapted with permission from ref (61). Copyright 2015 Nature.

**Figure 3:**
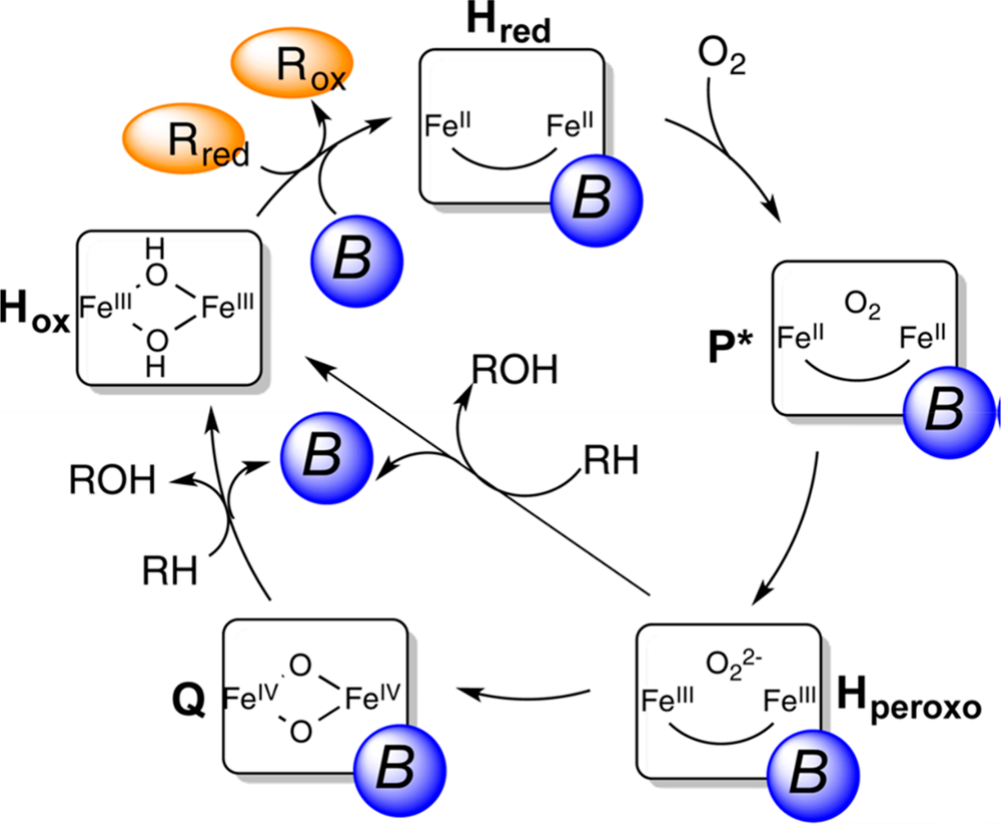
Catalytic cycle of sMMO. *R*_red_ and *R*_ox_ represent the reduced and oxidized reductase MMOR, respectively, and B is the regulatory component MMOB. Reproduced with permission from ref (62). Copyright 2015 American Chemical Society.

The active site for methane oxidation, often referred to as compound Q, comprises a diiron cluster, the precise structure of which was, until recently, a matter of debate. In 2015, Banerjee et al.^61^ solved the structure (Figure 2). The reductase activates the O_2_ delivering a hydroperoxy species to this diiron active center, and to achieve this it requires a nicotinamide adenine dinucleotide cofactor (NADH). The overall mechanism was described by Lippard and co-workers^62^ (Figure 3), which shows the interaction of the three components to bring about the overall hydroxylation of methane. The diiron active site of MMO is often used as a starting point for the design of chemocatalysts,^56,58,61,62^ as is the cyclic nature of the mechanism. However, the oxidation state of the iron is stabilized as Fe(IV) by the amino acids adjacent to the site, and this is not possible to readily replicate in a chemocatalyst. While sMMO can activate methane, methane is not the sole hydrocarbon that can be utilized as a substrate; sMMO can also use other hydrocarbons as substrates (such as substituted cyclohexane). Furthermore, in these reactions, there are aspects of regioselectivity,^63^ and no enantioselectivity is observed with prochiral substrates.

In terms of catalytic efficiency of methane activation, sMMO can react methane with 100% selectivity to methanol with a turnover frequency (TOF) of 95 mol_methanol_ mol_Fe_^–1^ h^–1^ with a turnover number (TON) of 19 and an activity of 5.05 mol kg_cat_^–1^ h^–1^ (50 °C, 12 min in water, O_2_/NADH).^64^ If hydrogen peroxide is used in place of O_2_ and the NADH cofactor, the activity is markedly decreased to only 0.027 mol kg_cat_^–1^ h^–1^ (50 °C, 12 min in water, O_2_/NADH). MMO only uses one of the oxygens in O_2_ selectively (see section 1.2). Therefore, 100% methanol selectivity is only possible because of the presence of a cofactor to scavenge the other oxygen. If methane is the only reductant present in the system (i.e., no other cofactor), there will be a stoichiometric limit on methanol selectivity of 80%.

sMMO is the only monooxygenase that can activate methane, but there are a range of other monooxygenases^58^ that can utilize a wide range of hydrocarbon substrates and the use of these enzymes in chemical transformations could be of great interest in the future. Recently, the prospects of using sMMO to make methanol as part of a gas to liquids process has been reviewed,^65^ and a number of challenges were identified that present obstacles should this route to exploit natural gas be pursued. These include gas–liquid mass transfer limitation and the potential poisoning of the enzyme by impurities in the natural gas. These will also be critical for any chemocatalysts operating in the liquid phase. However, the potential toxicity of methanol to sMMO at the higher concentrations of methanol that any commercial process requires could present a major hurdle to large scale utilization of sMMO.

pMMO comprises of three subunits (PmoA, PmoB, and PmoC), these are arranged as a trimer of the respective protomers. In contrast to sMMO, the active site is copper based and is considered to be located in PmoC.^60^ This copper site is denoted Cu_c_ and is associated with two other Cu centers in PmoB, however, these are not present in all pMMOs.^66^ Methane activity of this methane monooxygenase are related to conservation of the active center structure, and this is compromised greatly upon removal from the native membrane environment^67^ although not related to loss of copper ions, hence the prevalence of sMMO in the literature, as discussed above. However, it is possible to reconstitute pMMO into bicelles^67^ or more recently nanodiscs^57^ has afforded researchers an opportunity to meaningfully characterize the active centers of this enzyme, where methane activity is retained. In the case of reconstitution with nanodiscs, additional copper (as CuSO_4_) is required along with the native lipids to regain methane oxidation activity when used with the reductant duroquinol.^57^ A turnover frequency of 0.012 s^–1^ was reported which compares favorably to membrane bound studies on pMMO^68^ of ca. 0.026–0.042 s^–1^. The mechanism of methanol formation with duroquinol was recently proposed by Peng et al.,^69^ whereby a proton transfer reaction facilitates coordination of duroquinol to the Cu_c_(II) site in the PmoC subunit, followed by oxygen binding and hydrogen atom abstraction to release a dione. A secondary duroquinol molecule then undergoes a hydrogen atom abstraction to generate H_2_O_2_ and a coordinated Cu_c_(II)-duroquinol negatively charged species. An electron is transferred from the bound O^–^ of the duroquinol to the Cu_c_(II) to form Cu_c_(I), further electron transfer occurs to the coordinated peroxide from Cu_c_(I) to restore Cu_c_(II) state, followed by hydrogen atom abstraction on the now coordinated peroxy radical to release H_2_O and leave the Cu_c_(II)-O^•–^ methane active species. A further electron is transferred from the Cu_c_(II) to the coordinated duroquinol radical, allowing CH_4_ to react and generate CH_3_OH. The remaining duroquinol-Cu_c_(II) bound via an O^–^ species reacts with the protonated glutamine residue (Glu-H) to complete the reaction cycle. The activity afforded with duroquinol can be improved with NADH as the reductant, for example, the specific activity *Methylococcus capsulatus* (Bath) expressed as nmol mg_TOTAL PROTEIN_^–1^ min^–1^ was reported to be between 12 and 20 with duroquinol^68^ and 40–70 with NADH.^67^ In the case of using native lipids with the nanodisc methodology, the activity is retained at 7.2 nmol mg_TOTAL PROTEIN_^–1^ min^–1^.^57^

### 2.3 General Observations on Methane Partial Oxidation Using Heterogeneous Catalysis

**Figure 4:**
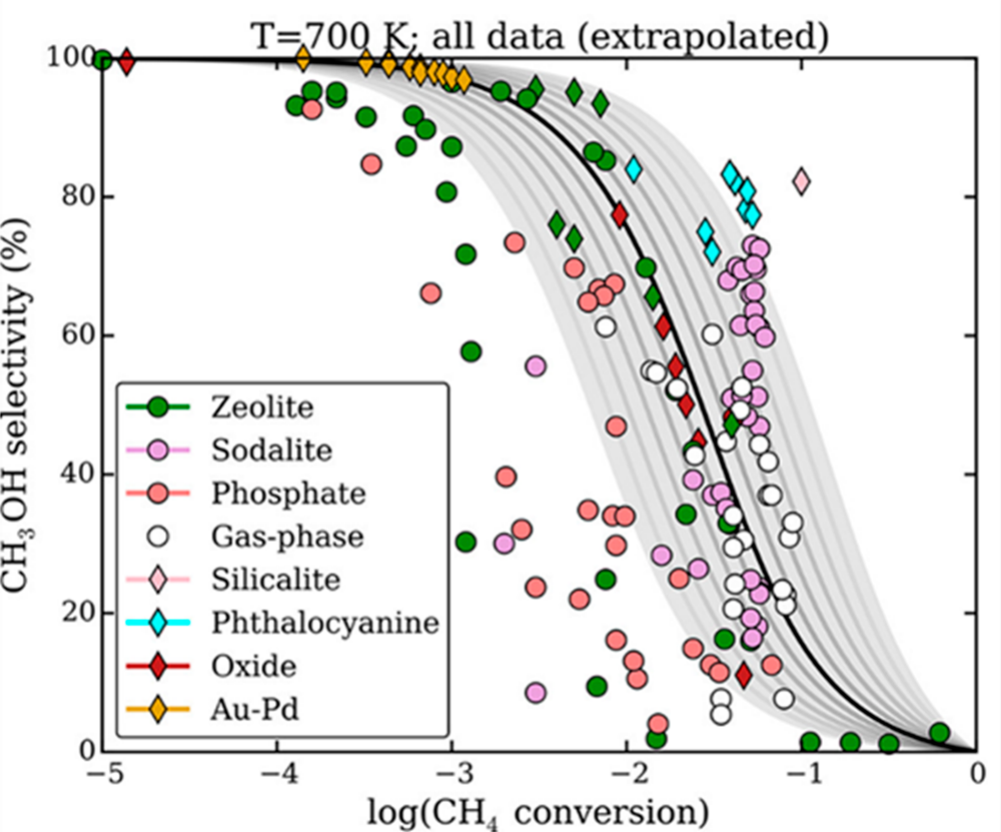
Experimental selectivities and conversions of single-site catalysts for methane oxidation to methanol. The image shows data whose selectivities have been extrapolated to the gas phase at 700 K, based on the relative rate constants for CH_4_ → CH_3_OH and CH_3_OH → CO_2_ derived from the difference between the free energies of activation for methane and methanol. Colors denote different catalyst morphologies, diamonds are aqueous experimental reaction conditions, and circles are gas phase. Reproduced with permission from ref (70). Copyright 2018 American Chemical Society.

A recent survey of catalytic methane to methanol oxidations^70^ shows some degree of consistency in the relationship between selectivity and conversion for systems described as having a single C–H bond activation site for methane and methanol via a radical pathway (Figure 4). This study uses a simple model based on the relative free energies of activation for methane and methanol to adjust for differing test conditions and compares a wide range of catalytic systems; homogeneous gas phase oxidation is also found to be consistent. This suggests that the desired performance is beyond the capability of systems, where the C–H bonds in methane and methanol can both react with similar active species without some further influence on reactivity. Suggested approaches include protecting groups (including bonding to a heterogeneous surface), in situ methanol “collectors”, and introducing diffusion control, such that C–H bond activation is no longer rate determining.^70^ Further strategies worth considering could include creating environments surrounding catalytic sites that reject methanol product, or in situ conversion to a more oxidation resistant derivative, possibly involving coproducts such as CO, the most likely candidate being acetic acid. Of course, a more complex, multisite catalytic mechanism may also show a different selectivity/conversion relationship, but the great majority of candidates reviewed were inferior in this respect.^70^

The relative free energy of activation model referred to above predicts very low methanol selectivity (i.e., <10%) at methane conversions above 1% and temperatures below ∼250 °C for gas phase/heterogeneous systems. It should be noted, of course, that reducing temperature is not expected to be beneficial when the undesired, further reactions of products have lower activation energies than the activation of methane. However, introducing liquid water improves the predicted selectivity/conversion relationship by 4 orders of magnitude at 50 °C^70^ via an assumed solvation effect on the relative free energies of activation. The performance of the limited number of aqueous systems reviewed appeared to somewhat exceed these lower temperature predictions, suggesting there may be additional benefits from having an aqueous environment, although the performance is still below economic targets.

Further insight into the possible role of water is provided by Bunting et al.^71^ In their DFT and ab initio molecular dynamics studies of relative methane and methanol activation, they point out that C–H activation may not be rate determining over certain catalysts (i.e., a number of face-centered-cubic (fcc) metal surfaces), with subsequent reaction of the surface bound intermediates *CH_3_ (from methane, with “*” indicating surface adsorbed species) or *CH_2_OH (from methanol) with *O/*OH having higher activation barriers. Nevertheless, relative activation barriers for these C–O bond forming reactions still always favors methanol oxidation over methane oxidation, both in heterogeneous/gas phase systems and in the presence of liquid water. However, in the aqueous phase, the relative abundance of *OH compared to *O is likely to be enhanced and the kinetics of coupling with these species becomes “kinetically indiscriminate”. This offers the prospect of favoring the direct coupling of *CH_3_ with *OH to form methanol directly and minimizing coupling of *CH_3_ with *O to form *CH_3_O, which can also form methanol but in competition with dehydrogenation to *CH_2_OH and further oxidation. Of course, liquid water may also serve to promote the conversion of *CH_3_O to methanol.

The two theoretical studies described above reinforce the observations made by other authors of a beneficial effect of an aqueous environment on methanol formation.^72,73^ Realistically, however, aqueous systems will produce a dilute methanol product, and new or emerging separations technologies will be needed to bridge the gap to an affordable, final distillation stage, most likely through pervaporation techniques.^74−77^ Accordingly, work on direct methane oxidation in aqueous media should seek methanol product concentrations of at least a few wt %.

There may be niche applications where the challenging performance criteria described in section 1.2 are not required, specifically where the product is not intended for conventional markets and high methane conversion or the use of high purity oxygen are not required. An example could be in very remote oil and gas operations, where methanol may be required for use as a local fuel, in gas processing, or for methane hydrate suppression, and where methanol import has a higher cost or has a high environmental impact.^31^ Other examples that have been described are in NO_*x*_ reduction for gas fired power generation and using coal-bed methane to methanol in conjunction with coal/methanol slurries.^33^ However, these niche opportunities may not be able to justify, or indeed require, the large investment usually required for major new catalytic process developments and may therefore be limited to the known gas phase partial oxidation reaction (described in section 2.1).

## 3 High Temperature Gas Phase Selective Methane Oxidation

### 3.1 Metal Oxide Catalysts

The direct gas phase selective oxidation of methane, with the aim to form the oxygenates methanol and formaldehyde, has been studied extensively, especially in the 1980s and 1990s. The approach generally used high temperatures, with a preference for metal oxide catalysts. Many of these more historical studies have been reviewed previously.^78−81^ Although a popular approach at the time, there are inherent issues with the catalytic high temperature gas phase approach. This section summarizes some of the key findings and sets out some of the issues encountered, which provide a basis to critically assess how more effective catalyst design approaches could be developed.

High temperature selective oxidation of methane has been investigated for many years. For example, in 1934, Wiezevich and Frolich investigated methane partial oxidation by O_2_ in a flow reactor at 132 bar.^82^ In an empty reactor tube, methane reacted at 500 °C and the temperature was lowered when natural gas was used as an alternative; at 390 °C, 30% of the condensable product was methanol. It was stated that the addition of iron, nickel, or aluminum catalysts to the reactor all increased the methanol yield, although no specific detailed results were reported.

Early work using heterogeneous catalysts was extended by Boomer and co-workers.^83−85^ At pressures around 180 bar with natural gas and O_2_ in the range 4.1–12.0%, copper was an effective catalyst for increasing the yield of methanol. Under these reaction conditions, it was concluded that Cu_2_O was formed on the surface of the copper catalyst, and it was postulated that the oxygen of the Cu_2_O was the active oxidizing species for methane. Any traces of sulfur in the reaction stream significantly deactivated the copper catalyst.

A wide variety of catalysts have been investigated for the high temperature gas phase partial oxidation of methane with the target of producing oxygenates. Many of the catalysts studied are metal oxides, and so initially it is interesting to focus on some studies that have adopted a catalyst design approach. One such pioneering study by Dowden et al. proposed a hypothetical *virtual mechanism*.^86^ Analyzing the thermodynamics of the target reaction and side reactions, it was concluded that the key catalyst functions required were dehydrogenation and oxygen insertion. Oxidation reactions all led preferentially to formation of undesirable carbon oxides. The mechanism anticipated that initial interaction of CH_4_ with the surface resulted in dissociation to form methyl and methylene species. It was important that further methyl and methylene dehydrogenation was suppressed relative to surface migration because further dehydrogenation led to carbon oxides. Consequently, the generation of methyl species was favored over the more strongly bonded surface methylene, thus directing catalyst selection toward a metal oxide in preference to a metal. The suggestion that the surface methyl bond should be weaker than the surface oxygen bond was important to promote methyl migration onto the oxygen. Suitably weak dehydrogenation functions were metal d^0^, d^1^, d^5^, d^10^, or d^4^ electron configurations, while the oxygen insertion properties should be those of typical n-type oxides, with recommended components TiO_2_, V_2_O_5_, Fe_2_O_3_, MoO_3_, and ZnO. These should be present in a single crystallographic phase, with the different functional sites adjacent to each other to allow rapid surface species migration.

To preserve oxygenate selectivity, the introduction of a hydration function to the catalyst was required. Hydration enhanced the formation of surface methylene diol, which by analogy with oxidation in aqueous solution is relatively slowly attacked by one-electron oxidizing species. Phosphates and tungstates, in conjunction with single electron oxidant transition metal ions, were postulated as favorable for this task. The hydration component would also enhance the production of methanol relative to formaldehyde. It was concluded that suitable catalysts should be formulated from
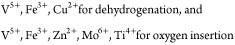
The *virtual mechanism* proposed was one of the first to develop a conceptual approach to selective methane oxidation, but it did not contain any significant experimental validation. However, the thinking obviously influenced a related patent by Dowden and Walker,^87^ who developed a series of two component oxide catalysts based on their mechanistic principles.^86^ Results were reported for MoO_3_/ZnO, MoO_3_/Fe_2_O_3_, MoO_3_/VO_2_, and MoO_3_/UO_2_ supported on ^1^/_3_Al_2_O_3_/SiO_2_ with a low surface area of ca. 0.1 m^2^g^–1^ and a loading of 5% active oxide. The best catalyst contained MoO_3_/Fe_2_O_3_, which showed a combined selectivity to CH_3_OH and HCHO of 80% at 3.5% methane conversion, yielding 869 and 100 g kg_cat_^–1^ h^–1^ of methanol and formaldehyde, respectively. Experimental conditions of 30 bar at a temperature of 430–500 °C, coupled with injection of liquid water to cool the reactor effluent within 0.3 s of leaving the catalyst bed, was required to maintain the high yields.

**Figure 5:**
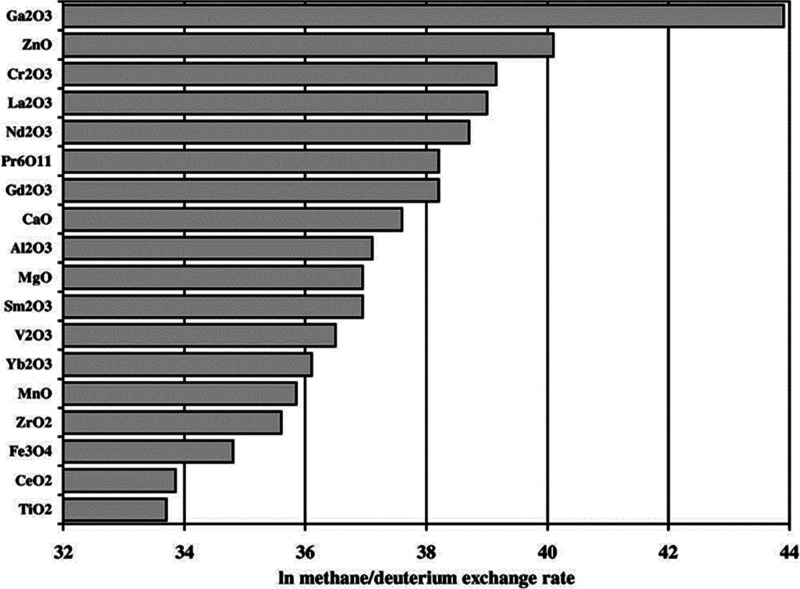
Rate of methane–deuterium exchange over a range of metal oxides at 500 °C normalized for the effect of surface area. Conditions: CH_4_ = 0.69 mL min^–1^, D_2_ = 0.83 mL min^–1^, GSHV = 290 h^–1^. Reproduced with permission from ref (91). Copyright 2002 Elsevier.

In another design approach, the activation of the reactants (CH_4_ and O_2_) and the desired methanol product have been considered over single metal oxides. The aim was to choose components effective for activating methane and oxygen, while preserving methanol, and then combining the components to promote catalytic synergy. MoO_3_ was identified as a potential catalyst component because even though it was effective for selective oxidation of methanol to formaldehyde, there was little further oxidation to carbon oxides at high temperatures.^88^ Furthermore, MoO_3_ showed exchange of the entirety of its lattice oxygen with the gas phase oxygen. The diffusion of oxygen throughout the lattice of the oxide was faster than the surface exchange, which was therefore the rate determining process. The exchange mechanism for these oxides operated by a combination of surface processes.^89,90^ The activation of O_2_ and the diffusion of oxide species throughout the lattice are recognized as important concepts of oxidation catalysts. Methane activation was assessed by isotopic exchange experiments between CH_4_ and deuterium,^91^ as the exchange reaction may be considered the first indicator of catalytic CH_4_ activation. The oxide Ga_2_O_3_ demonstrated a surface normalized rate of CH_4_/D_2_ exchange several orders of magnitude greater than any other oxide (Figure 5). Hence a 1:1 Ga_2_O_3_/MoO_3_ catalyst prepared by physical mixing was proposed, and the catalyst demonstrated significant activity for methane selective oxidation to formaldehyde.^92,93^ The addition of the Ga_2_O_3_ component increased methane conversion while maintaining the high selectivity of MoO_3_, thus validating the design approach.

Otsuka and Hatano investigated methane partial oxidation by O_2_ over a range of metal oxides supported on silica. They established a volcano-type relationship between conversion and cation electronegativity, while formaldehyde selectivity increased with increasing electronegativity;^94^ these relationships were used to rationally design catalysts. The correlations were explained by considering the role of electronegativity on the relative rates of initial H abstraction from CH_4_, oxygen insertion to form formaldehyde, and abstraction of H from HCHO to form carbon oxides. Based on these principles, it was concluded that acidic oxides would not be efficient for methane selective oxidation because, although high HCHO selectivity could be achieved, H abstraction from CH_4_ was inefficient. A B_2_O_3_/SiO_2_ catalyst was chosen because it had the highest formaldehyde selectivity, and other components were added to enhance H abstraction from CH_4_. Adding BaO and MgO to the B_2_O_3_/SiO_2_ system produced the greatest formaldehyde yields in the initial study. Extending the approach, a mixed oxide with the overall composition 1:2:2 Fe:Nb:B was developed, and it contained the phases FeNbO_4_, FeNb_11_O_29_, and B_2_O_3_.^95^ FeNbO_4_ was suggested to be responsible for methane activation and oxygen insertion to form formaldehyde, while B_2_O_3_ minimized overoxidation and direct oxidation of CH_4_ to CO*_X_*. At 870 °C and atmospheric pressure, an HCHO space-time-yield (STY) of 1210 g kg_cat_^–1^ h^–1^ was achieved, which represents one of the highest reported yields in the direct conversion process.

Lyons and co-workers^96−98^ have also used a design approach to develop a high temperature gas phase catalyst based on the principles of the cytochrome P450 enzymes. These oxidize methane to methanol and are thought to function via a high oxidation state ferryl species capable of alkane activation. A conceptual model was proposed in which the redox potential of the Fe^2+^ center was modified, suppressing the irreversible conversion to the Fe^3+^-O-Fe^3+^ μ-oxo complex in favor of the Fe^3+^-O-O-Fe^3+^ μ-peroxo species, which facilitates formation of the active ferryl Fe^5+^=O species.

The catalyst developed by Lyons et al.^97^ was a sodalite microporous framework with >10 wt % Fe substituted for Al^3+^ in the framework positioned at exchangeable sites. Calcination at 550 °C was required to form the most active catalyst, credited to partial framework collapse, and corroborated by XRD and EPR evidence, that drove Fe from framework sites into exchangeable positions associated with residual framework Fe to create an active center. A conceptual mechanistic pathway, based on the development of framework and extra-framework Fe interactions was proposed, with CH_4_ being activated at a surface generated ferryl intermediate, resulting in the release of methyl radicals to the gas phase. A 70% methanol selectivity at 5.7% conversion was achieved under operating conditions of 3:1 CH_4_:air at 416 °C, 53 bar pressure, and a GHSV of 530 h^–1^.

Another independent study of the Fe-sodalite catalyst by Betteridge et al., which reproduced the same reaction conditions, gave 33% methanol selectivity at 3.1% conversion.^99^ The apparent differences in activity between the two studies may be due to differences in reactor design, as the work of Lyons et al. mentioned the importance of a reactor bypass facility.^96^ Betteridge et al.^99^ confirmed the presence of Fe^3+^ in the sodalite framework in the synthesized catalyst, while postreaction Fe^2+^ species were identified along with dispersed <1 μm iron oxide particles, which were shown to be very effective for oxidizing methanol to carbon oxides. Theoretical studies indicated that a framework Fe^2+^–Fe^3+^ redox couple was the most energetically favorable site configuration. Calculations also showed that methane was not able to diffuse into the sodalite framework, thus limiting catalytic activity to the external crystallite surface.

One of the most widely used catalyst components for selective methane partial oxidation is molybdenum oxide, and such catalysts can be categorized into two general groups, namely (i) catalysts using bulk MoO_3_ crystals as the basis material and (ii) those which utilize a highly dispersed molybdenum species on a high area support.

Notable examples of MoO_3_-based catalysts have been mentioned previously when considering design approaches,^87,93^ but there are also many other examples described in the literature. One of the most active catalysts was reported by Stroud when investigating dual component metal oxide catalysts, with MoO_3_ as one of the components.^100^ The other component was one that must exhibit redox behavior and the oxides of Cu, Fe, Co, Ni, Cr, V, Sn, and Bi were all considered suitable. The best catalyst was found to be CuO/MoO_3_, producing an oxygenated product yield of 540 g kg_cat_^–1^ h^–1^ (at 19 bar pressure, 485 °C, and GHSV = 46700 h^–1^). The yield of oxygenates formed included C_2_H_5_OH and CH_3_CHO, as well as CH_3_OH and HCHO, because C_2_H_6_ was a major constituent (6.1%) of the initial natural gas feed employed. The presence of ethane was an important factor, one indeed acknowledged by Stroud. Gesser et al.^101^ have reviewed many cases when ethane was present in minor amounts in methane_,_ and deduced that it served to reduce the initial reaction temperature and enhance methanol yields when compared to pure methane.

Iron–molybdenum oxide catalysts have also been investigated by Otsuka et al., particularly focusing on Fe_2_(MoO_4_)_3_ catalysts.^102^ At atmospheric pressure, a formaldehyde selectivity greater than 75% was observed at low methane conversion (0.24%) at 650 °C, decreasing to 30% at 7.8% conversion (750 °C). Their experiments showed that formaldehyde was formed from the sequential oxidation of methanol, which is consistent with the known efficacy of iron molybdate phases for methanol selective oxidation to formaldehyde.^103^ Carbon oxides were derived from the oxidation of formaldehyde. Based on differences of product distributions in the presence and absence of catalyst and differences in the change of methane conversion with varying residence time, the authors concluded that the reaction mechanism was exclusively heterogeneous. Considering the high reaction temperatures employed, this deduction may seem somewhat counterintuitive, but a specially engineered reactor was used which tapered from 8 mm i.d. at the inlet to 1.5 mm i.d. at the outlet, which was designed to help to minimize gas phase reactions. When the oxidant was switched to N_2_O from O_2_, product selectivity switched from oxygen insertion products to methane coupling products such as C_2_H_6_ and C_2_H_4_.^104^

**Figure 6:**
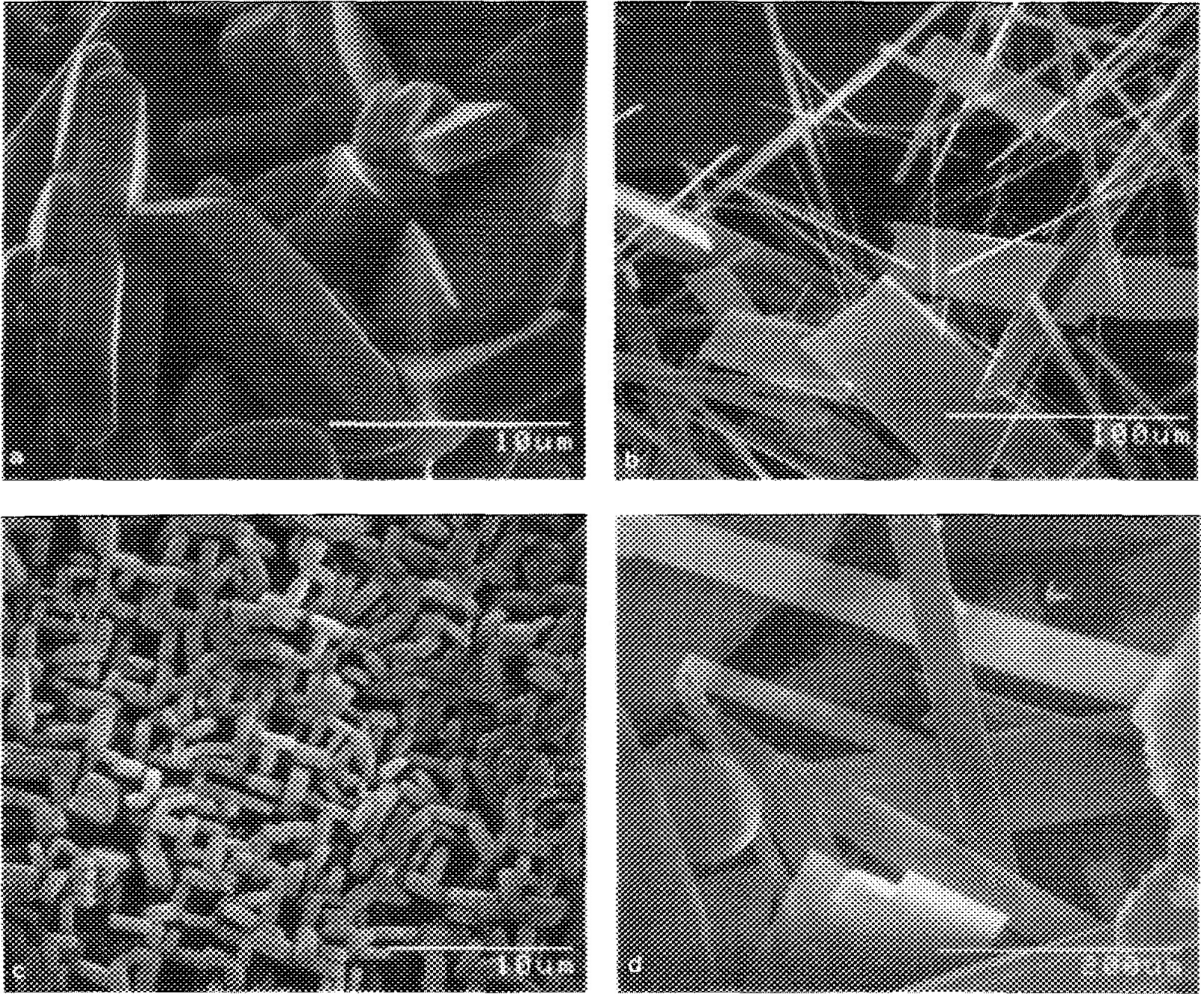
Scanning electron micrographs of MoO_3_ catalysts prepared under different conditions to vary the ratio of (010) basal planes to (100) side planes. Prepared by (a) MoO_3_ heated under nitrogen (MoO_3_-C); (b) cooling of molten MoO_3_ (MoO_3_-R); (c) oxidation of thin Mo metal sheet; (d) vapor deposition of MoO_3_. Reproduced with permission from ref (105). Copyright 1993 Elsevier.

An important study by Smith and Ozkan probed the effect of morphology and exposed surface facet planes for methane selective oxidation by MoO_3_.^105^ Several MoO_3_ catalysts were prepared to vary the ratio of (010) basal planes to (100) side planes (Figure 6); the MoO_3_-R catalyst preferentially exposed the (010) plane, while the MoO_3_-C variant exposed a greater number of (100) planes. The MoO_3_-C catalyst was more selective toward formaldehyde than MoO_3_-R by a factor of 2, with this structure sensitivity being evident over a range of varying CH_4_ and O_2_ concentrations. It was proposed that Mo=O sites, residing preferentially on the (100) plane, were active for selective oxidation, while Mo-O-Mo bridging sites, mainly on the (010) plane contributed to complete and sequential oxidation. In situ laser Raman spectroscopy, TPR, and ^18^O_2_ labeling studies further concluded that gas phase O_2_ directly reoxidized Mo-O-Mo sites, while Mo=O sites were reoxidized by diffusion of oxygen from the MoO_3_ lattice.

The most studied catalyst for gas phase CH_4_ selective partial oxidation is molybdenum oxide supported on high area SiO_2_. One of the earliest studies was reported by Liu et al. using a 1.7 wt % Mo/SiO_2_ catalyst with N_2_O as oxidant.^106^ A combined selectivity to methanol and formaldehyde of 84.6% was achieved at 8.1% methane conversion, when steam was co-fed with the reactants at 560 °C. A later more detailed publication from the same research group^107^ was unable to reproduce the catalyst performance from the earlier study. They reported that the combined oxygenated selectivity was lower at 78.7% at 2.9% conversion. EPR spectroscopy identified oxygen species on the surface from N_2_O decomposition, concluding that these species were responsible for the nonselective oxidation reactions. Surface O^–^ species formed from the interaction of N_2_O with Mo^5+^ species were also identified and proposed as the active sites for selective oxidation by H abstraction from CH_4_ to produce methyl radicals. A surface methoxide anion was formed by the reaction of methyl radicals with Mo^5+^O^–^ sites, resulting in the formation of methanol and formaldehyde.

Khan and Somorjai also investigated a silica-supported MoO_*x*_ catalyst for the selective oxidation by N_2_O^108^ and were able to reproduce the earlier catalyst performance.^107^ Kinetic analysis showed that below 540 °C formaldehyde and methanol were derived from parallel routes, while at higher temperatures formaldehyde was produced from a methanol intermediate. The role of co-fed water in both of these studies was an important factor. The specific role of water in the reaction mechanism was not clear, but thermal and radical quenching events can be envisaged. Khan and Somorjai also suggested that co-fed water prevented the deposition of carbonaceous material as no coking was evident on the catalyst.

Molybdenum oxide on silica catalysts have also been investigated using O_2_ rather than N_2_O as the oxidant. Spencer showed that the major reaction products were HCHO, CO, and CO_2_, although some trace amounts of CH_3_OH and H_2_ were also detected.^109^ The best catalyst was MoO_3_ supported on Cab-O-Sil silica, prepared by physical milling. Catalysts prepared by impregnation routes also proved to be active but less selective. Sodium impurities were important, and it was shown that concentrations as low as 300 ppm had a detrimental effect on methane conversion and selectivity to partial oxidation products. Further studies demonstrated that sodium impeded direct methane oxidation to formaldehyde and CO_2_, while promoting oxidation to CO.^110^ Initial methane activation was proposed to take place at a Mo–O^•^ surface radical species, generated thermally at the reaction temperature, and Mo^5+^ species were also postulated to be important in several of the reaction steps.

The identity of the support for highly dispersed molybdenum oxide species has an important role for methane selective oxidation. MgO and TiO_2_ supports resulted in the sole production of carbon oxides, while under the same reaction conditions using Spher-O-Sil (porous silica) and Cab-O-Sil (fumed silica) supports, formaldehyde was formed.^111^ The detrimental effect of sodium was once again confirmed, as it suppresses formaldehyde selectivity.^112^ Addition of alkali metal cations to the Mo/SiO_2_ catalyst was also studied in more detail.^113^ Such catalysts were doped with Na, K, and Cs, which formed new surface alkali molybdate species and decreased methane conversion and formaldehyde selectivity. In the absence of alkali metal cations, isolated MoO_*x*_ species were present, and the activity observed correlated well with the number density of these species.

The influence of oxidant, Mo loading, and silica support for MoO_*x*_/SiO_2_ catalysts was studied by Banares et al.^114^ Significant differences in activity and selectivity were observed over a range of Mo loadings with surface concentrations from 0.3 to 3.5 Mo atoms/nm^–2^ (0.5–16.2 wt %). Both methane conversions and formaldehyde selectivities were higher using O_2_ rather than N_2_O, indicating that O_2_ was the preferred oxidant. It was proposed that a Mars–van Krevelen mechanism operated and O_2_ was more effective at reoxidizing the catalyst. Further studies by the same group concluded from ^18^O_2_ tracer studies that oxygen from the catalyst was incorporated into the formaldehyde product, confirming the Mars–van Krevelen mechanism.^115^ However, employing oxygen isotope exchange and steady state oxygen isotope transient techniques, Mauti and Mims concluded that no information on the oxygen source for formaldehyde could be obtained.^116^ This was due to the substantial and rapid oxygen exchange of HCHO with the catalyst, through a reversible acetal surface species formed by reaction of HCHO with Mo=O sites.

Formaldehyde yield was maximized at a loading of 1 Mo atom nm^–2^, irrespective of the oxidant employed.^114^ Raman spectroscopy, XPS, and XRD studies indicated uniformly distributed Mo species interacting strongly with the silica surface, with loadings below 0.8 Mo atoms nm^–2^ forming a highly dispersed molybdate phase, while crystalline MoO_3_ was formed at higher loadings.

Depending on the Mo loading, three different species have been identified on the silica support and attempts have been made to correlate the structures with activity for methane selective oxidation.^117^ Loadings of 1–5 wt % showed a strongly interacting uniformly distributed phase of silicomolybdic acid (SMA). Polymolybdate species were formed at 5–10 wt %, and these covered the SMA but not the support. At 15 wt % loading, SMA was no longer detected, and crystalline MoO_3_ was formed. When using N_2_O as the oxidant. there was a direct correlation between the concentration of SMA and formaldehyde selectivity for lower loading catalysts.

Similar results to Barbaux et al.^117^ have been reported by Kasztelan et al.,^118^ as they also correlated methane selective oxidation activity with surface SMA, although the overall yields of partially oxidized products were low. The concentration of SMA was dependent on the pH of the molybdenum preparation solution rather than variation of Mo loading.

Smith et al. have also investigated the nature of the surface species on the MoO_*x*_/SiO_2_ catalyst and corroborated the presence of three surface Mo species.^105^ Below 2 wt %, there was a silicomolybdic species, and a surface coordinated polymeric molybdate was identified as the loading increased. At loadings above 3.5 wt %, crystalline MoO_3_ was again detected, with the polymolybdate species coexisting up to the highest loading examined at 9.8 wt %. The activity of the catalyst was once more found to be dependent on the Mo loading. A large decrease of methane conversion was observed at 5 wt % MoO_*x*_ loading, corresponding to an appreciable amount of crystalline MoO_3_ on the surface. The catalyst with the lowest MoO_*x*_ loading, 0.5 wt %, was the best, which again had the most dispersed silicomolybdic phase. Silicomolybdic species have terminal Mo=O sites, and these were postulated to be the active sites for the selective oxidation to formaldehyde. The Mo-O-Mo bridging species were thought to be nonselective oxidation sites, and their number increased at the expense of the terminal Mo=O sites as the MoO_*x*_ loading increased.

There is general agreement between these studies as to the type of supported molybdenum species present, but some differences are apparent, and the species formed are sensitive to preparation conditions. One important factor is the pH of the heptamolybdate solution used for impregnation. The Mo species in solution is dependent on the equilibrium:
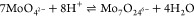
At pH 6, the Mo_7_O_24_^6–^ ion is predominant with Mo in an octahedral environment, while MoO_4_^2–^ tetrahedra form at pH 11. In addition to controlling the Mo species, pH also affects the net surface charge of the support and consequently dispersion. Ismail et al. have characterized the silica supported Mo species after impregnation to 8 mol % loadings at varying pH. At pH 6, both MoO_3_ islands and crystallites were present, with Mo in tetrahedral and octahedral coordination environments, respectively. MoO_3_ crystallites were again identified at pH 11, while in contrast preparation at pH 1 gave a highly dispersed silicomolybdate phase.

Molybdenum has also been supported on ultrastable zeolite Y, exhibiting low formaldehyde selectivity at low methane conversion.^119^ The best catalyst prepared by impregnation was limited to activity from MoO_3_ crystallites located on the external surface of the zeolite and performance correlated with MoO_3_ dispersion. Although some Mo ions were located within the framework cavities, they were inactive, further demonstrating the importance of the molybdenum species for effective catalysis and highlighting the range of species that have been proposed as being active.

Silica supported vanadium oxide catalysts have also been extensively used for gas phase selective methane oxidation, with one of the earliest investigations being reported by Somorjai and co-workers.^120^ Spencer and Pereira reported that VO_*x*_/SiO_2_ selectively oxidized methane to formaldehyde using O_2_, producing high selectivity at low conversion.^121^ Formaldehyde oxidation experiments showed that CO was the primary product and followed a sequential oxidation mechanism to CO_2_. Direct comparison with MoO_*x*_/SiO_2_ showed the silica supported vanadium oxide catalyst was more active.

Kennedy et al. showed that formaldehyde yields under both methane rich and lean conditions were dependent on the vanadium oxide loading on the silica support.^122^ Optimum yields were obtained when the V loading was in the range 1–4 wt %. Over this range formaldehyde selectivity was constant, so yield was controlled by methane conversion. Activity was related to the redox properties of the vanadium species, where higher loadings showed reduced yields because vanadium was reoxidized slowly, while low vanadium loadings did not possess sufficient extractable oxygen. Therefore, it was only those catalysts with loadings between 1 and 4 wt % that were able to have a sufficiently high reoxidation rate and supply of extractable lattice oxygen.

Kartheuser and Hodnett demonstrated a relationship between the dispersion of vanadium oxide on SiO_2_ and formaldehyde selectivity.^123^ The dispersion was measured by reduction of NO by NH_3_ at 200 °C and measuring evolution of N_2_. Initial N_2_ formation was used to quantify surface V=O sites and subsequently dispersion.^124^ Determined at constant conversion, maximum formaldehyde selectivity was achieved at maximum vanadium dispersion. It was postulated that higher selectivity was achieved over smaller vanadium oxide particles because they were less efficient for further formaldehyde oxidation due to fewer active oxygen sites on small domains compared to larger ones. The same conclusion was also derived by Chen and Wilcox over a similar catalyst when methane was oxidized with either O_2_, N_2_O, or a combination of both.^125^

Insight into the mechanism of the VO_*x*_/SiO_2_ catalyst has been provided by a temporal analysis of products (TAP) approach.^126^ Oxygen interacted strongly with the catalyst surface, producing a species with a long active lifetime between 5–60 s. Conversely, CH_4_ surface interaction was very weak, leading to very short lifetimes. Surface oxygen species activated methane, forming methyl radicals, which reacted further with the catalyst, extracting lattice oxygen that was incorporated to form formaldehyde.

Further mechanistic investigation showed that the ability of VO*_x_*/SiO_2_ to exchange oxygen with gaseous O_2_ was low in the absence of methane, but when methane and O_2_ were present simultaneously, the rate was increased by a factor of ca. 4.^127^ This increase was attributed to a redox mechanism, which only operated when methane was present. It was confirmed that oxygen associated with the catalyst was involved in formaldehyde production, as well as CO and CO_2_, but the contribution from lattice oxygen could not be determined due to the considerable secondary oxygen exchange of these products. These conclusions were similar to those drawn for the MoO*_x_*/SiO_2_ catalyst.^116^

The studies on silica supported Mo and V oxides have employed both O_2_ and N_2_O as oxidants, and catalyst performance has been evaluated using a range of conditions. However, some general conclusions can be drawn around the established reaction pathways. Kinetic analysis concluded that the MoO_*x*_/SiO_2_ catalyst oxidized methane to the primary products formaldehyde and CO_2_ via parallel reaction pathways through a common activation step.^109,110^ CO was produced by subsequent oxidation of formaldehyde and could be further oxidized to CO_2_. Conversely, methane oxidation over VO_*x*_/SiO_2_ followed a sequential pathway, with formaldehyde as the only primary product, which was oxidized first to CO then sequentially to CO_2_.^121^ Microkinetic simulations indicated that yields of selective oxidation products could be optimized over VO_*x*_/SiO_2_ by careful engineering of the reactor geometry to reduce overoxidation, while the inherent formation of CO_*x*_ by MoO_*x*_/SiO_2_ could not be reduced by an engineering approach.^128^

The extensive research on supported molybdenum and vanadium oxide catalysts has established that silica was an excellent support, and SiO_2_ alone has also been used as a catalyst for this reaction. Kasztelan and Moffat showed that a commercial Grace-Davidson 400 grade silica was active for methane partial oxidation.^129^ Formaldehyde selectivity was 10% at 0.7% methane conversion at 514 °C and ambient pressure with O_2_ oxidant, replacing O_2_ with N_2_O, and CO was the major product. A wider range of silicas has also been investigated, demonstrating that Cab-O-Sil (fumed silica), Ludox silica gel, and silicic acid were all active for forming formaldehyde.^118^ All the silicas showed similar reactivity trends to empty reactors at 620 °C and elevated pressure. The activation energy for HCHO formation was independent of the catalyst, concluding that formaldehyde was formed from a homogeneous gas phase reaction. CO and CO_2_ activation energies were dependent on the catalyst used, possibly indicating the catalyst was responsible for formaldehyde oxidation. Ethane was present in the methane feed, and this has been shown to enhance selective oxidation products.^100,101^

Parmaliana et al. also investigated silica catalysts at a lower temperature (520 °C), including precipitated, extruded, fumed, and gel variants.^130^ Precipitated silicas were the most active, producing a formaldehyde STY at least 3 times greater than extruded or sol–gel silicas, with fumed silica showing very poor performance. In contrast, Sun et al. obtained appreciable formaldehyde STYs over fumed silica and silica gel catalysts at a high temperature of 780 °C.^131^ Ethane was also a significant product, with selectivities greater than formaldehyde, and both were determined to be primary products, with formaldehyde postulated to be formed from a surface methoxy species and C_2_H_6_ from gas phase methyl radical coupling.

Other metal oxides commonly used as catalyst supports have also been evaluated. Parmaliana et al. showed that γ-Al_2_O_3_, MgO, TiO_2_, and ZrO_2_ all predominantly formed CO and CO_2_, with low selectivity to ethane over MgO and ZrO_2_.^130^ A similar conclusion was reached by Kastanas et al., observing mainly total oxidation products over γ-Al_2_O_3_ and MgO.^132^

Kobayashi et al. investigated the effect on the partial oxidation of CH_4_ by doping a high area silica with 0.05 atom % of 3d transition metal ions.^133^ At a high space velocity and 600 °C, bare SiO_2_ showed low activity for formaldehyde formation. Addition of the metal ions enhanced the yield in all cases, and it was most pronounced by Fe^3+^ addition, which increased formaldehyde STY by an order of magnitude over SiO_2_. The activity was attributed to highly dispersed isolated metal ions, as catalysts comprising of the simple oxides only formed CO_*x*_. The Fe catalyst was the most active due to the efficient redox cycle of the Fe center.

Chun and Anthony investigated a range of catalysts for selective methane oxidation at a relatively high pressure of 48 bar.^134^ These included SiO_2_, TiO_2_, mixed and single oxides of Fe, Mo, Cu, V, and Sn, Ag/γ-Al_2_O_3_, and Pyrex beads. At temperatures required for almost complete O_2_ conversion, the product distributions were all similar and not affected by the catalyst, indicating that homogeneous reactions in the void volume of the catalyst bed were significant with respect to heterogeneous reactions. The presence of an oxide surface was also responsible for inhibiting free radical homogeneous reactions. The study emphasizes the important contribution of homogeneous gas phase reactions during methane selective oxidation, especially at elevated pressure. Consequently, the influence of surface reactions is diminished, and it is difficult to control product selectivity at the high temperatures required to activate gas phase methane over metal oxide catalysts.

**Figure 7:**
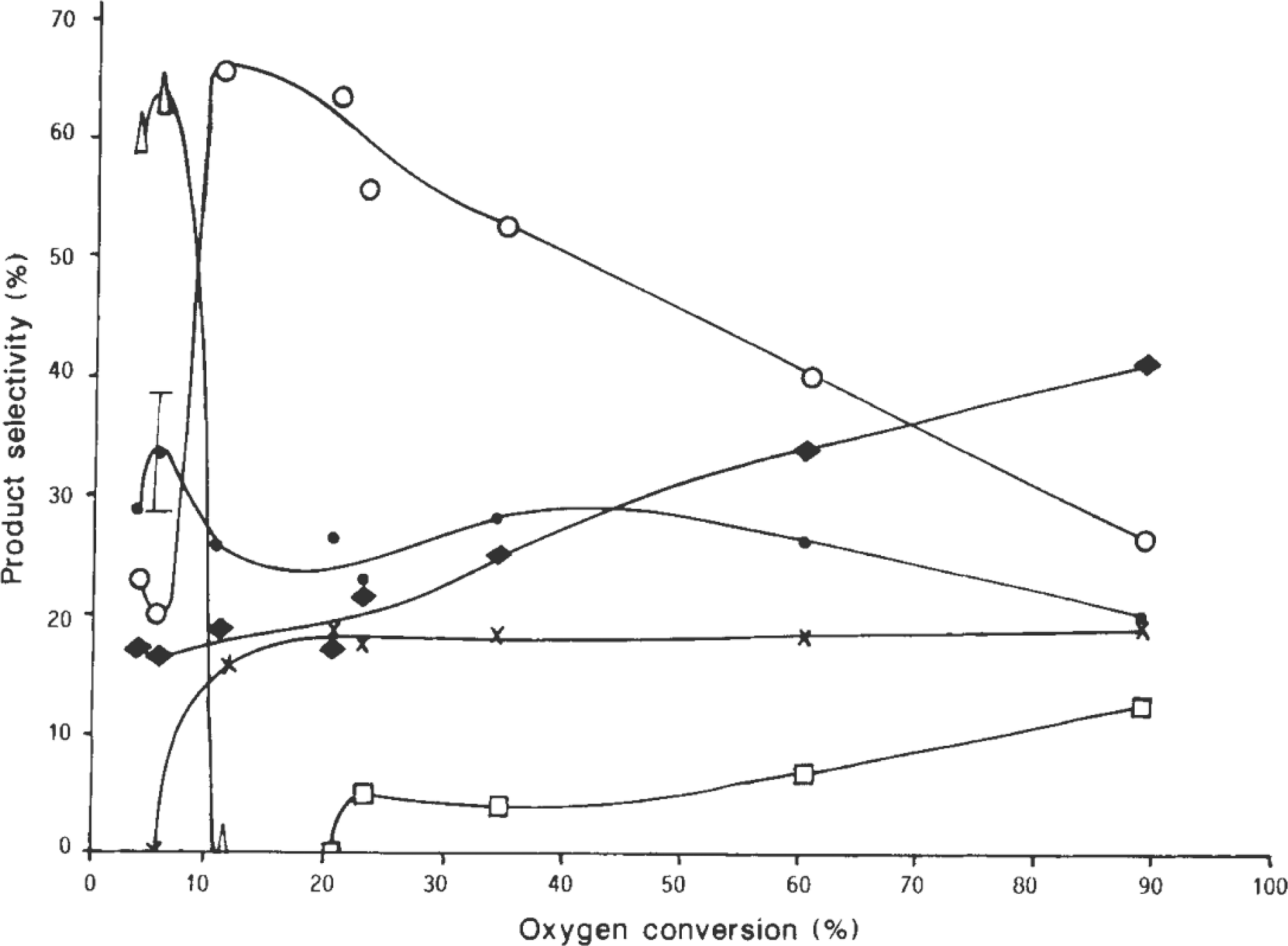
Changes in selectivity in methane conversion over a magnesium oxide catalyst as a function of flow rate and oxygen conversion. χ, ethane; □, ethene; ○, carbon monoxide; ◆, carbon dioxide; ●, hydrogen; Δ, formaldehyde. Values are accurate to ±1% at oxygen conversions >60%, but only to ±5% at conversions <10%. Solid lines are guides to the eye. Reproduced with permission from ref (135). Copyright 1990 Springer Nature.

Hargreaves et al. showed how MgO, recognized as an effective methane oxidative coupling catalyst, can be switched to produce formaldehyde with a significant STY at 750 °C by control of the reaction conditions.^135^ The switch from C_2_H_6_ (and CO) to HCHO was accomplished by increasing the GHSV of the reactant feed (Figure 7). Below 10%, O_2_ conversion selectivity to formaldehyde was significant but decreased as O_2_ conversion increased, while ethane and CO selectivity increased. The selectivity switch was rationalized by considering the possible reactions of methyl radical intermediates. The concentration of gas phase radicals decreased linearly as the GHSV was increased, and because ethane formation was proportional to the square of the radical concentration, production declined. Whereas, at low oxygen conversion, there was a relatively high O_2_ partial pressure in the catalyst bed and methyl radicals reacted preferentially with O_2_, leading to formaldehyde.

The same type of product switch has also been demonstrated by Sinev et al. for methane selective oxidation by O_2_ over Fe, Zn, and Zr phosphate catalysts at 725 °C.^136^ The selectivity switch was controlled by increasing the O_2_ partial pressure of the reactant feed, at lower values formaldehyde was the dominant product, but it decreased at the expense of ethane and CO as O_2_ concentration increased. In agreement with the earlier study on MgO,^135^ it was concluded that formaldehyde and ethane were derived from the common methyl radical intermediate. Based on observed differences between catalysts, the formation of HCHO was not purely a gas phase process, and there was an influence from surface reactions. The same iron phosphate catalyst was studied further and cofeeding water increased formaldehyde formation, and the major product was formic acid.^137^

In contrast to the selectivity switches regulated by reaction conditions, Sojka et al. demonstrated a selectivity switch by chemical modification of a ZnO catalyst.^138^ In the temperature range 500–850 °C, methane in air was oxidatively coupled to C_2_ products and oxidized to CO. Doping with low amounts of equimolar concentrations of Cu^1+^ and Fe^3+^ switched the main product to formaldehyde, attributed to the Cu and Fe redox couples and the Lewis acid properties of Fe^3+^. These functionalities were proposed to trap methyl radicals on surface sites, which were subsequently oxidized to surface methoxide.

In a deliberate strategy to exploit gas phase radicals, a double layered catalyst bed of Sr/La_2_O_3_ and MoO_*x*_/SiO_2_ is described by Sun et al.^139^ The first 1 wt % Sr/La_2_O_3_ bed was selected to provide a flux of methyl radicals to the MoO_*x*_/SiO_2_ bed, which would convert the radicals to formaldehyde. Adding the Sr/La_2_O_3_ bed before the MoO_*x*_/SiO_2_ bed had a detrimental effect on selectivity, for example, at 630 °C, selectivity was decreased from 100% to 3.3%, however, methane conversion increased substantially from 0.08% to 8.2%, resulting in an increase of formaldehyde STY. Mixing the beds together resulted in a decrease of formaldehyde STY by 2 orders of magnitude.

A novel approach at the time by Wada and co-workers, reported the use of UV radiation to enhance oxygenates from catalytic methane oxidation over MoO_*x*_/SiO_2_^140^ and MoO_*x*_/ZnO.^141^ Reaction temperatures were lowered significantly (190–277 °C), and irradiation of MoO_*x*_/SiO_2_ and MoO_*x*_/ZnO produced formaldehyde as the major reaction product with traces of methanol, and no carbon oxides were produced. When the UV source was removed neither catalyst showed any activity.

The more historical studies outlined in this section have shown that a wide range of metal oxide-based catalysts have been developed and evaluated for gas phase direct selective oxidation of methane to oxygenates. One feature that is apparent from many studies is the inverse relationship between methane conversion and selectivity toward methanol and formaldehyde. Many studies have reported high oxygenate selectivity, 100% in some examples, but it was only high at low methane conversion, consequently per pass yields were very low. This phenomenon can be related to the very high temperatures that are required to activate CH_4_ over metal oxide catalysts, and the subsequent overoxidation of the desired oxygenated products to the more thermodynamically favored carbon oxides. Nevertheless, some appreciable STYs of oxygenated products have been achieved when very low contact times were used, but the very low conversions achieved are far from ideal.

It is clear from the studies presented in this section that the mechanism of methane selective oxidation over the metal oxide catalysts is complex, and many different mechanisms have been proposed over many different catalysts. Although claims have been made for purely surface mediated mechanisms, it appears more likely that both heterogeneous and gas phase homogeneous reactions are involved. This seems likely, particularly considering the high reaction temperatures which have to be used and the beneficial effects encountered when increasing the pressure. The contribution from homogeneous gas phase reactions introduces additional complexity to control selectivity, hence approaches that can activate methane under much milder conditions and maximize surface reactions, while minimizing gas phase reactions, would offer a more effective strategy for designing better selective methane oxidation catalysts.

### 3.2 Microporous Materials with N_2_O as Oxidant: α-Oxygen

The use of N_2_O as an oxidant has received much attention for methane oxidation over polyoxotungstates^142^ or silica supported catalysts,^107,120^ but particularly over iron containing zeolites which is the focus of this subsection. As discussed, zeolites have many properties that can facilitate selective methane oxidation; among these are a confinement effect,^143^ thermal stability, and an ability to host mono- or binuclear active sites.^144,145^ For example, Fe or Cu sites present in zeolitic structures have been reported and used in both liquid^144,146^ and gas phase reactions.^147^ Examples of gas phase reactions are the oxidation of benzene to phenol and methane to methanol using N_2_O over Fe-modified ZSM-5 catalysts.^148−150^ Nitrous oxide decomposition^151^ (eq 1) can be achieved over many types of catalysts, including perovskites,^152−156^ ceria-based catalysts,^157−159^ spinels,^160−162^ and iron containing zeolites.^147,163,164^ In the last case, H-ZSM-5 has been frequently used as a support.^165−167^ Crucially, the use of nitrous oxide over modified-zeolite catalysts results in an oxidized metal site that can facilitate methane oxidation through, what is commonly termed, an active α-oxygen species (eq 2).
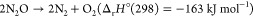
1
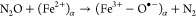
2The following literature examples illustrate the conditions required to decompose N_2_O. Xie et al. reported complete decomposition of N_2_O at 450 °C over a 7.64 wt % Fe-ZSM-11.^168^ In contrast, Wood et al.^169^ reported 84% conversion at 500 °C using an 0.57 wt % Fe-ZSM-5 catalyst. Sobalik et al.^170^ reported that with an equivalent Fe loading, the Si:Al ratio was crucial for N_2_O decomposition with ferrierite (FER); a catalyst with a Si:Al ratio of 8.5 outperformed a catalyst with a Si:Al ratio of 10.5. Further, Rauscher et al. confirmed that catalysts with low Si:Al ratios were effective for N_2_O decomposition.^165^ A comparison of the performance in N_2_O decomposition for different zeolite structures was reported by Melián-Cabrera et al., Fe-ZSM-5 (Si:Al = 11.4) achieved 95% conversion of N_2_O at 500 °C, while, in contrast, Fe-BEA achieved just 20% conversion at 575 °C.^171^ The high temperatures used to decompose N_2_O are not commensurate with the reaction conditions discussed in early reports regarding methanol or phenol formation,^148^ and so these systems should really be thought of as stoichiometric transfer reagents. However, the formation of α-oxygen can occur at temperatures below 200 °C, so that, in principle, catalytic turnover for low temperature methane partial oxidation is possible. The Si/Al ratio is an important factor for activity of an N_2_O decomposition catalyst, and so it can be reasoned that this factor plays an important role in producing the active oxygen species for methane oxidation. The Si:Al ratio can also dictate the metal loading, and hence the density of active sites that can be achieved. For example, the active Fe site is thought to form in close proximity to Al in the zeolite framework so that low Si:Al ratios should be able to accommodate a higher concentration of active sites.^172^

**Figure 8:**
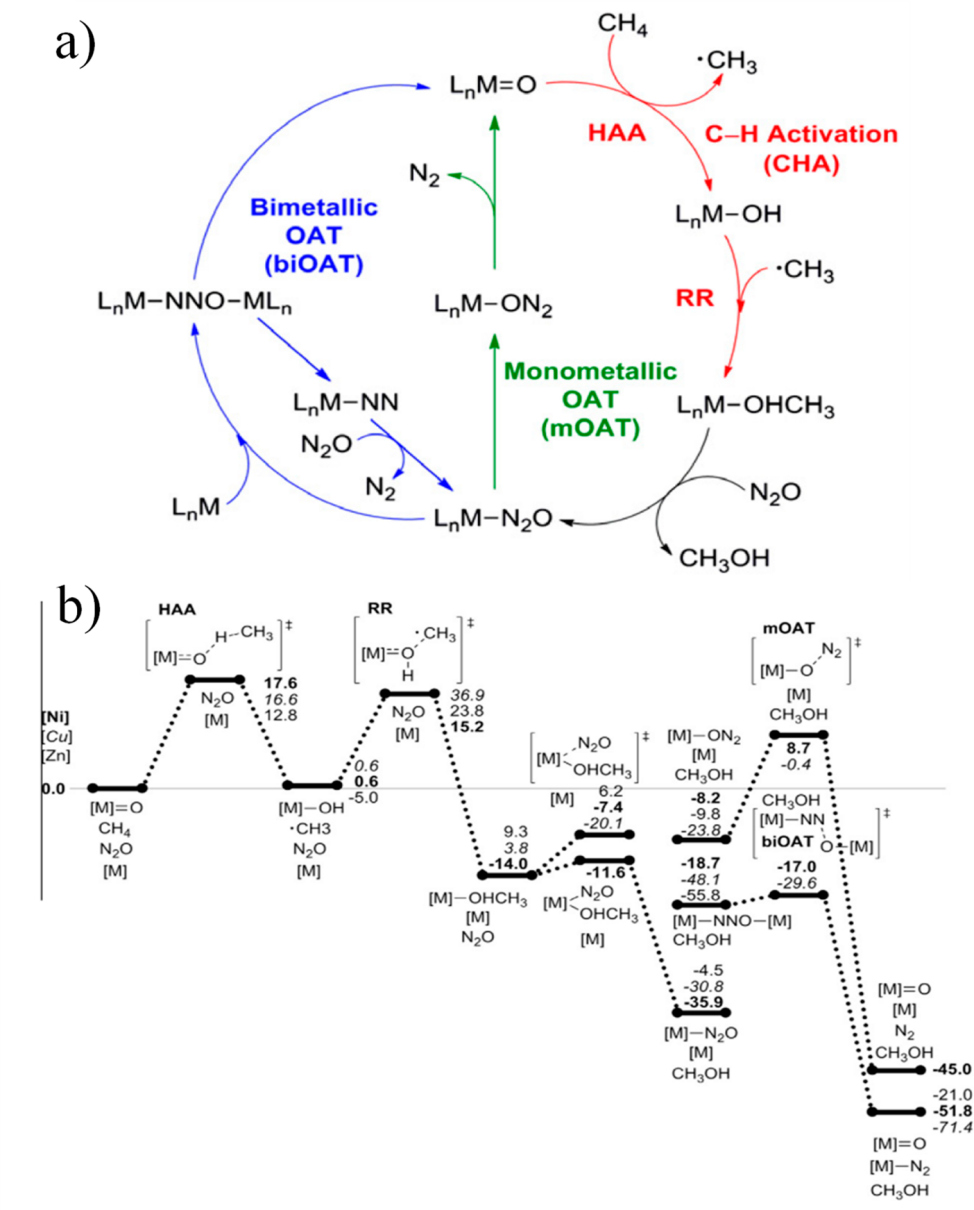
(a) General mechanism for methane oxidation to methanol by metal oxo groups. HAA, hydrogen atom abstraction; RR, radical rebound; OAT, oxygen atom transfer. (b) Calculated free energy surface (B3LYP/6-311+G(d)) for M = Ni, Cu, and Zn. The metal cations are stabilized in a metal complex with a bidentate CH_5_N_2_ ligand as shown for the [Cu]=O example inset in (b). energies are in kcal mol^–1^. Adapted with permission from ref (174). Copyright 2013 Elsevier.

Homogeneous metal catalysis using N_2_O as oxidant for methane to methanol has also been considered. The general catalytic cycle developed is shown in Figure 8a. Oxygen atom transfer (OAT) from N_2_O to the metal center creates a metal oxo group and placing the metal center in a high oxidation state (formerly, Cu^3+^=O, Ni^3+^=O and Zn^3+^=O), methane undergoes C–H activation (CHA) to reduce the metal center and create an M–OH radical species which combines with the ^•^CH_3_ fragment in a radical rebound (RR) step to produce methanol which, in homogeneous reactions, can be displaced by N_2_O. Cundari and co-workers have mapped out the free energy surface for this scheme using hybrid density functional theory calculations (Figure 8b).^173,174^ Their calculations indicate that the highest barrier along the pathway is the C–H bond activation step for Ni, but the radical rebound step becomes rate determining for Cu and Zn when a model bidentate CH_5_N_2_ ligand is used to form the complexes. The calculations also highlight that the most efficient route to displacing methanol with further oxidant is via bimetallic OAT involving two metal complexes. At the concentrations possible for extra-framework cations in zeolites, it is likely that only the monometallic OAT route would be possible.

#### 3.2.1 Fe-Containing Zeolites

The formation of the α-oxygen active site for methane oxidation is related to that proposed for the decomposition of N_2_O, where the active oxygen species remains on an Fe site rather than recombining. An early example of the reactive nature of this site is found in benzene oxidation to phenol over Fe-ZSM-5 with N_2_O.^148,149,175^

**Figure 9:**
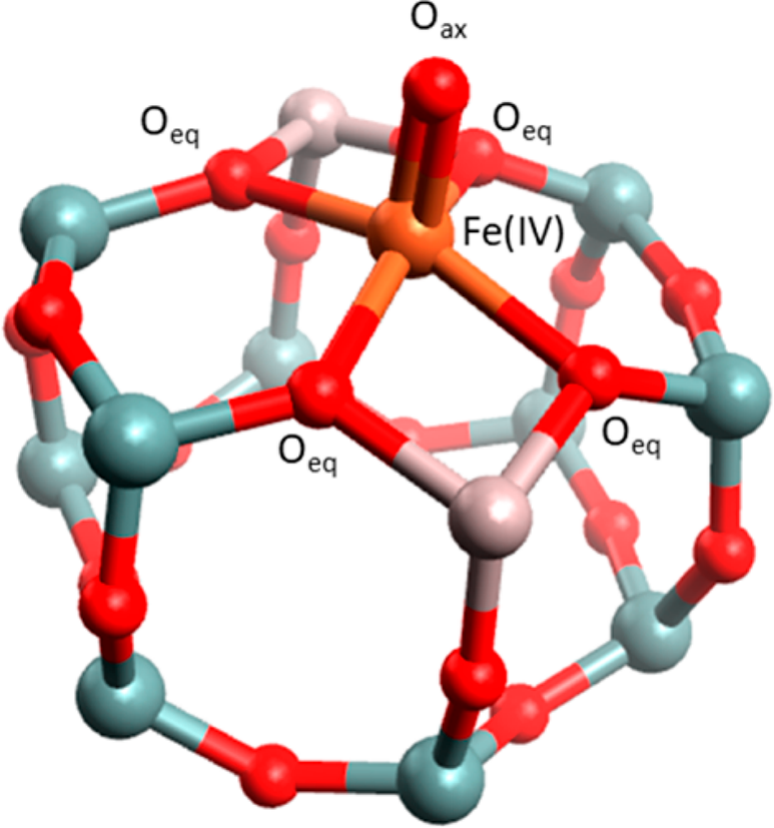
Simulated BLYP structure illustrating the Fe^4+^=O α-oxygen site within a six-member ring of the Fe-CHA zeolite structure. Adapted with permission from ref (188). Copyright 2018 American Chemical Society.

The addition of a reductant in the feed-stream can facilitate the abstraction of oxygen from the oxidized active site, greatly increasing the N_2_O decomposition rate at reduced temperatures.^169,176−179^ For example, propane has been reported as an effective reductant in the N_2_O decomposition reaction,^180−183^ as have CO, ethane and methane.^184−186^ Furthermore, oxidative dehydrogenation of propane can be achieved over metal-modified zeolites, and this topic has been recently reviewed by Jiang et al.^187^ The exact nature of this site is now better understood, and its formation is schematically illustrated in Figure 9,^145,188,189^ which has been adapted from the detailed study by Bols et al.^188,189^ For some time, the nature of the active site was debated, with literature proponents supporting either mono- or dinuclear Fe sites as being responsible for the decomposition of N_2_O and the subsequent formation of the active oxygen species.^149,190−192^ In either case, extra-framework Fe is considered to be the active site for the formation of an α-oxygen species,^193−198^ which is formed by decomposing N_2_O over a reversible redox α-Fe^2+^ site.^189,199,200^ After this oxygen addition it has been suggested that either mononuclear Fe^4+^=O^2–^ (or Fe^3+^-O^•–^), similar to the species considered in Figure 8, or dinuclear Fe as an oxo-bridged Fe^3+^O^2–^Fe^3+^ species were the most appropriate candidate models for the α-oxygen active site.^200−202^ Snyder et al.^145^ reported that a mononuclear α-Fe^2+^ in an extra-lattice site was present in Fe-β zeolite (BEA), based on magnetic circular dichroism spectroscopy.

**Figure 10:**
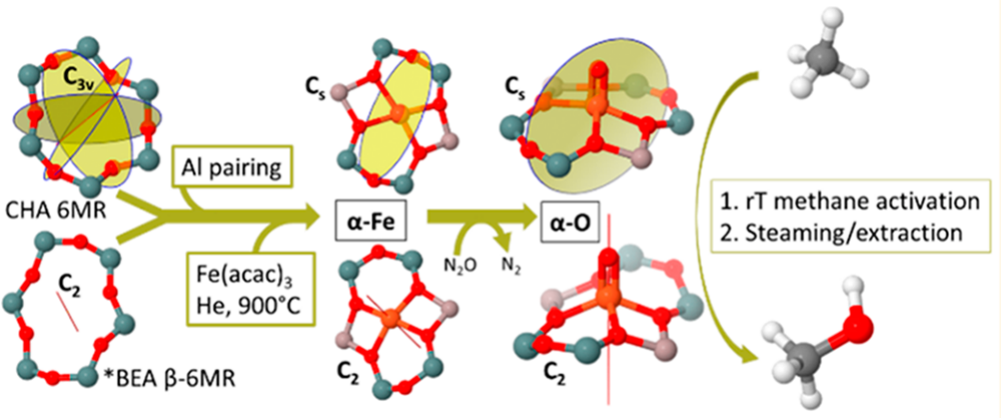
Reaction schematic the introduction of α-Fe into six-membered rings within zeolite (CHA 6-MR or *BEA β-6MR), the reaction of α-Fe with N_2_O to produce α-oxygen and then methane activation, radical rebound to produce methanol which is extracted from the zeolite by steaming. Color scheme: C, light gray; H, white; O, red; Fe, orange; Si, gray; Al, light brown. Reproduced with permission from ref (188). Copyright 2018 American Chemical Society.

A high spin Fe^4+^=O species (Figure 9) was described as the reactive intermediate with confinement of methane within the zeolite pores facilitating the reactivity observed. Mössbauer spectroscopy has also been used to investigate the structure of the active sites of Fe-ZSM-5, and it was found that the active oxygen species with adjacent Fe^2+^ ions existed as mononuclear sites upon decomposition of N_2_O in Fe-ZSM-5. Recently, Bols et al. have confirmed through DFT and Mössbauer, FT-IR and diffuse reflectance UV–vis–NIR spectroscopy studies that the active site comprises an Fe^2+^ that is the precursor to Fe^4+^=O as the α-oxygen intermediate (Figure 10). Maximizing the number of these species would therefore be a distinct advantage to enhancing methanol yields, and a study by Bols et al. has reported the preparation of a Fe-containing zeolite, whereby >70% of Fe is in the α-Fe^2+^ form.^203^

Initially, the selective oxidation of methane over Fe-modified zeolites was achieved in a cyclic process, that is, the α-oxygen species is first formed and then reacted with methane in a separate step, followed by the desorption or extraction of methanol. Ovanesyan et al.^201^ reported in 1998 that an α-oxygen species was responsible for the formation of methanol. This system has subsequently been investigated extensively by Panov and co-workers.^200,204,205^ They reported that methane was activated over Fe-ZSM-5 by an α-oxygen species formed on the Fe center when N_2_O was used as the oxidant. Typically, the catalyst was pretreated at a range of temperatures (>500 °C) to convert the Fe^3+^ present into an Fe^2+^ state, which are referred to as the α-Fe site. The catalyst was then exposed to nitrous oxide to form the α-oxygen species. Crucially, they reported that the surface α-oxygen species could not be generated with molecular oxygen due to the strong stabilization of the parent ZSM-5 zeolite. The radical anionic nature of the α-oxygen species facilitated the cleavage of the methane C–H bond via hydrogen abstraction, which could proceed at room temperature.^200,203^

The active site undergoes a structural rearrangement to facilitate the formation of the α-oxygen by decomposing N_2_O over the reversible redox α-Fe sites, which revert to Fe^3+^. Panov and co-workers demonstrated that a three-step process could be used to form methanol: first, an N_2_O pretreatment of Fe-ZSM-5 was needed to form the α-oxygen species, second, the feed-gas was switched to methane to perform the stoichiometric methane-to-methanol oxidation, and last, methanol had to be extracted from the catalyst. Even at room temperature, the methoxy and hydroxyl groups formed can be subsequently adsorbed on the α-Fe sites, which can yield methanol directly on the surface of the zeolite.^206^ This process can be described as quasicatalytic, as the methanol formed needs to be extracted from the catalyst surface via hydrolysis; in this case, a solvent system consisting of a mixture of acetonitrile and water was used and with this with methodology a 94% selectivity to methanol was achievable.

**Figure 11:**
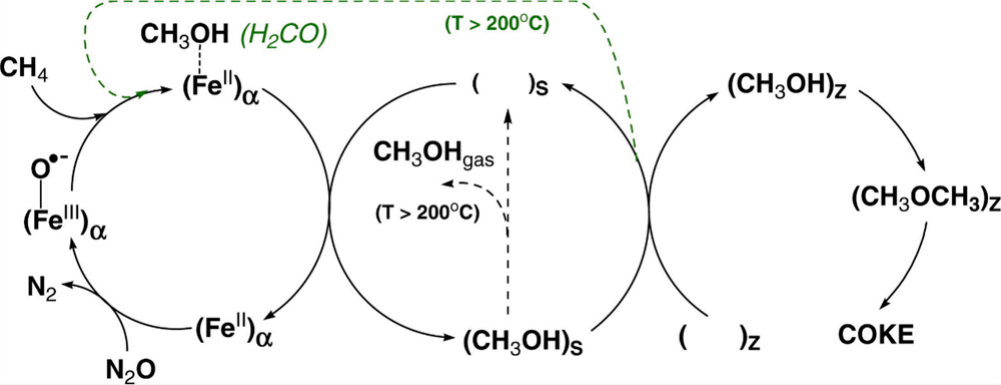
Mechanistic scheme of quasicatalytic and catalytic oxidation of methane. Solid lines indicate the steps that are present in both the quasicatalytic and catalytic modes of the reaction. Dotted lines display the steps that are present only in the catalytic mode. Adapted with permission from ref (200). Copyright 2014 Elsevier.

If this process is operated at an appropriate temperature, it can be fully catalytic^200,207,208^ under continuous flow conditions; however, deactivation is observed through active site blocking by carbon (Figure 11). The influence of surface acidity was investigated by Chow et al.^208^ over MFI zeolites. The presence of Al was necessary to form the active cationic form of the Fe species; however, methanol was found to be unstable over Brønsted acid sites. Carbon deposits were formed via the hydrocarbon pool mechanism, significantly limiting the methanol selectivity and yield. These deposits can accumulate rapidly (<1 h), limiting the methanol yield and reducing the carbon mass balance to ca. 40%. However, as Chow et al. revealed, the conversion of methane was not significantly reduced and over a 2.5 h reaction period remained at ca. 2%.^208^ Furthermore, carbon deposits or coke were reported to be a secondary product, whereby the methanol produced migrates to a Brønsted acid site and initially undergoes a reaction consistent with the methanol to olefins process (MTO).^200,207^

**Figure 12:**
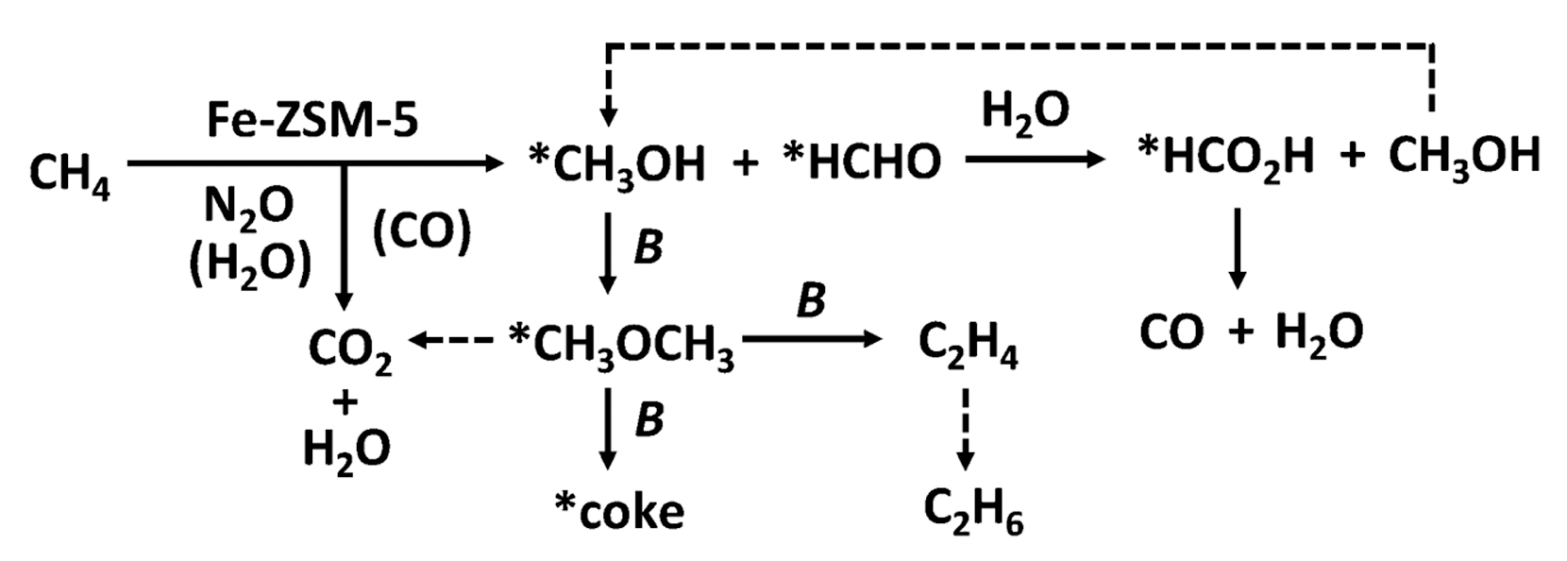
Proposed reaction network for CH_4_ oxidation with N_2_O over Fe-ZSM-5 catalysts according to delplot analysis; B is Brønsted acid site, and * indicates adsorbed or intermediate species not detected in the reactor effluent. Reproduced with permission from ref (207). Copyright 2018 Wiley-VCH.

Crucially, cofeeding water with CH_4_ and N_2_O aided the desorption of methanol from the catalyst, boosting stability and minimizing selectivity to coke.^200,209^ Chow et al. used a Delplot method to explore the reaction pathways and the influence of water on the reaction mechanism (Figure 12).^208^ They found that water acts to desorb methanol, and as such the carbon mass balance is greatly improved, beginning at >80% (*t* = 0.5 h) and decreasing slightly to 70% over the reaction period of 2.5 h online. The lower activity of the catalyst under these conditions was ascribed to the loss of active Fe sites, resulting in a concomitant decrease in N_2_O activation. The use of water in the feed has a secondary benefit through the reduction of C2 products in the postreactor stream. In this case, the decrease was ascribed to the loss of Brønsted acidity, which limits the formation of C_2_H_6_ from C_2_H_4_ via the hydrocarbon pool mechanism (Figure 12).^208−211^

#### 3.2.2 Different Frameworks

Methane oxidation is not limited to Fe containing MFI framework zeolites; indeed, much of the work on computationally and experimentally elucidating the active site has been achieved with beta (BEA) or chabazite (CHA) zeolites.^188,189^ Early work in this field by Knops-Gerrits and Goddard III demonstrated the influence of the framework structure, Fe-Fe/O/Si distances, pore dimensions, and the Si:Al ratio on the cyclic methanol extraction process following formation of α-oxygen.^212^ They investigated the α-sites of [Fe]-CIT-5, [Fe]-MOR, [Fe]-ZSM-5, and [Fe]-CHA (where Fe was in the zeolite synthesis solution) as compared to those where Fe was added postsynthesis. They found that the addition of Fe, postsynthesis resulted in active catalysts, and the yield of methanol was greatest over 5% Fe-ZSM-5.

Zhao et al.^213^ directly compared the activity and product distribution from reaction over Fe-ZSM-5, Fe-BEA, and Fe-FER catalysts. Framework acidity, pore character, and composition were assessed on the basis of both methanol yield and N_2_O decomposition. The findings suggested that the higher Al content of Fe-FER was crucial to stabilize the active Fe center, which was reflected in a greater methanol yield (TOF_CH4_ 125 h^–1^ with 200 mg catalyst at 350 °C). The lower Lewis acidity and smaller pore dimensions of Fe-ZSM-5 resulted in increased coking and subsequent deactivation (TOF_CH4_ 88.1 h^–1^ with 200 mg catalyst at 350 °C). Zhao et al.^214^ also investigated two preparation methods to illustrate the differences between catalyst formed via solid state (SSIE) and liquid ion exchange (IE). N_2_O conversion at 360 °C was higher over the Fe-FER-IE catalyst. At low methane conversion levels (<2%) the major products were dimethyl ether (DME) and CO, whereas the Fe-FER-SSIE catalyst was more selective to DME.

Different preparative methods of Fe-Faujasite Y catalysts were investigated by Zhu et al.^215^ Solid state ion exchange (Fe-Y-O) and incipient wetness impregnation (Fe-Y-I) methods were used to assess the influence of the catalyst preparation method on selective methane oxidation. Zhu et al.^215^ suggested from this work that incipient wetness impregnation gave an appreciable concentration of Fe_2_O_3_ particles that could be observed via TEM, whereas these entities were absent on the Fe-Y-I sample. This helped explain why the concentration of α-Fe sites as determined by N_2_O titration was 63% higher for the Fe-Y-O sample than the Fe-Y-I catalyst, and hence the methanol yield was significantly improved over Fe-Y-O. However, further analysis suggested the active site in this catalyst formulation was comprised of extra-framework dinuclear Fe^2+^ complexes.

Addition of extra-framework Al to increase the α-Fe site density was reported by Li et al.^216^ Mordenite (MOR) was chosen to illustrate this synthetic strategy in which aluminum nitrate was added to the zeolite along with ferrocene prior to activation. Three samples were characterized by ^27^Al MAS NMR following different levels of treatment and revealed that the structure could support more Al in extra-framework positions. Analysis of the comparative quantities of octahedrally coordinated Al (i.e., extra-framework) and tetrahedral Al (i.e., framework) indicated that the extra-framework sites increased in concentration, whereas the framework Al did not. Subsequently, the selectivity to methanol and DME was found to be highest over the Fe-MOR material with the highest Al content.

Clearly, the framework structure, Al loading, Fe:Al ratio, surface acidity (Brønsted and Lewis), and pore character all contribute to efficient methanol production (Table 1). The effectiveness of the catalyst can be related to the density of 6-membered rings (Figure 9) within the zeolite structure,^203^ as this is believed to be the position that provides optimal stabilization for the active site. To progress this technology further, the zeolite with the greatest loading of α-Fe must be prepared and reaction conditions optimized to promote methanol yields at the expense of CO or carbon deposits. Limitations with respect to methane conversion, typically less than 10% must be accounted for in any future reactor design. Ideally, both methane and N_2_O can be sourced from waste streams (e.g., biosourced off gas and adipic or nitric acid production), and this may prove invaluable in future applications. However, the need for a co-fed solvent such as water to reduce carbon deposition and maintain methanol desorption does reduce the scope of this approach due to postreaction processing costs.

**Table 1:** Comparison of Methanol Yields over Fe-Containing Zeolites with N_2_O as the Oxidant from Continuous Flow Reactions

catalyst	catalyst mass (g)	reaction temp (°C)	flow conditions flow rate (mL min^–1^) GHSV (h^–1^) (feed composition)	methane conv (%)	methanol STY or selectivity	ref
2% Fe-ZSM-5(30 Si:Al)	0.44	300	55 mL min^–1^	1.7	@55 min	(208)
			3600 h^–1^(39:10:1Ar:CH_4_:N_2_O)		14.3 μmol g^–1^ h^–1^	

2% Fe-ZSM-5 (30)	0.44	300	55 mL min^–1^	1.1	@65 min	(208)
			3600 h^–1^(29:10:10:1Ar:CH_4_:H_2_O:N_2_O)		98.9 μmol g^–1^ h^–1^	

Fe-MOR (3.4% Al)	0.2	300	20 mL/min	0.9	12.2% CH_3_OH select	(216)
Fe-MOR (3.9% Al)	0.2	300	20 mL/min	1.3	16.8% CH_3_OH select	(216)
Fe-MOR (4.3% Al)	0.2	300	20 mL/min	1.4	16.4% CH_3_OH select	(216)
Fe-Y-O (solid-stateion-exchange)	0.2	375	60 mL/min(1:1:1He:N_2_O:CH_4_)	*ca*. 4	6.2% select	(215)
Fe-Y-I (incipient wetness impregnation)	0.2	375	60 mL/min(1:1:1He:N_2_O:CH_4_)	*ca*. 1	1.5% select	(215)
Fe-FER	0.2	350	70 mL/min(65:28:7He:CH_4_:N_2_O)	2.8% @60 min	21% select	(213)
Fe-FER SSIE (0.5% Fe)	0.2	318	70 mL/min(65:28:7He:CH_4_:N_2_O)	1.5	15% (DME 50%)	(213)
Fe-FER IE (0.5% Fe)	0.2	318	70 mL/min(65:28:7He:CH_4_:N_2_O)	1.5	14% (DME 42%)	(214)

## 4 Liquid Phase Selective Methane Oxidation

### 4.1 Homogeneous Catalysis

The very considerable body of work on methane functionalization by homogeneous catalysis has been comprehensively reviewed by Periana and colleagues^217,218^ and by Chepaikin et al.^219^ Homogeneous catalysis often provides a greater opportunity for mechanistic analysis than heterogeneous catalysis, and we refer readers to these insightful, expert reviews for this interpretation. In this article, we will limit our comments to summaries of selected work to exemplify some of the approaches taken, focusing on catalytic systems using O_2_ or O_2_ regenerable oxidants.

Continuous processes employing homogeneous, dissolved catalysts in a liquid phase reactor generally require a separate step for disengaging catalyst and product so that the catalyst can be returned to the reactor. In principle, introducing additional process steps could be economically feasible providing these are efficient in both materials and energy, under conditions that are compatible with the overall process recycles. Examples of such additional steps could include recovering methanol from a protected intermediate product or regeneration of an oxidizing coreagent for recycle to the reactor. This regeneration step would need to use molecular oxygen to be economically feasible.

#### 4.1.1 Methanol via Methyl Esters

The most successful approaches to date within this area employ electrophilic metal complexes in strongly acidic conditions to form methyl esters (i.e., CH_3_OSO_3_H or CF_3_CO_2_CH_3_), a common feature of proposed mechanisms being C–H activation to form an L_*x*_M-CH_3_ intermediate without immediate change in formal oxidation state of the metal M. The electron withdrawing effect of the ester groups provides product protection by inhibiting further reaction of the remaining C–H bonds in the methyl group.

**Figure 13:**
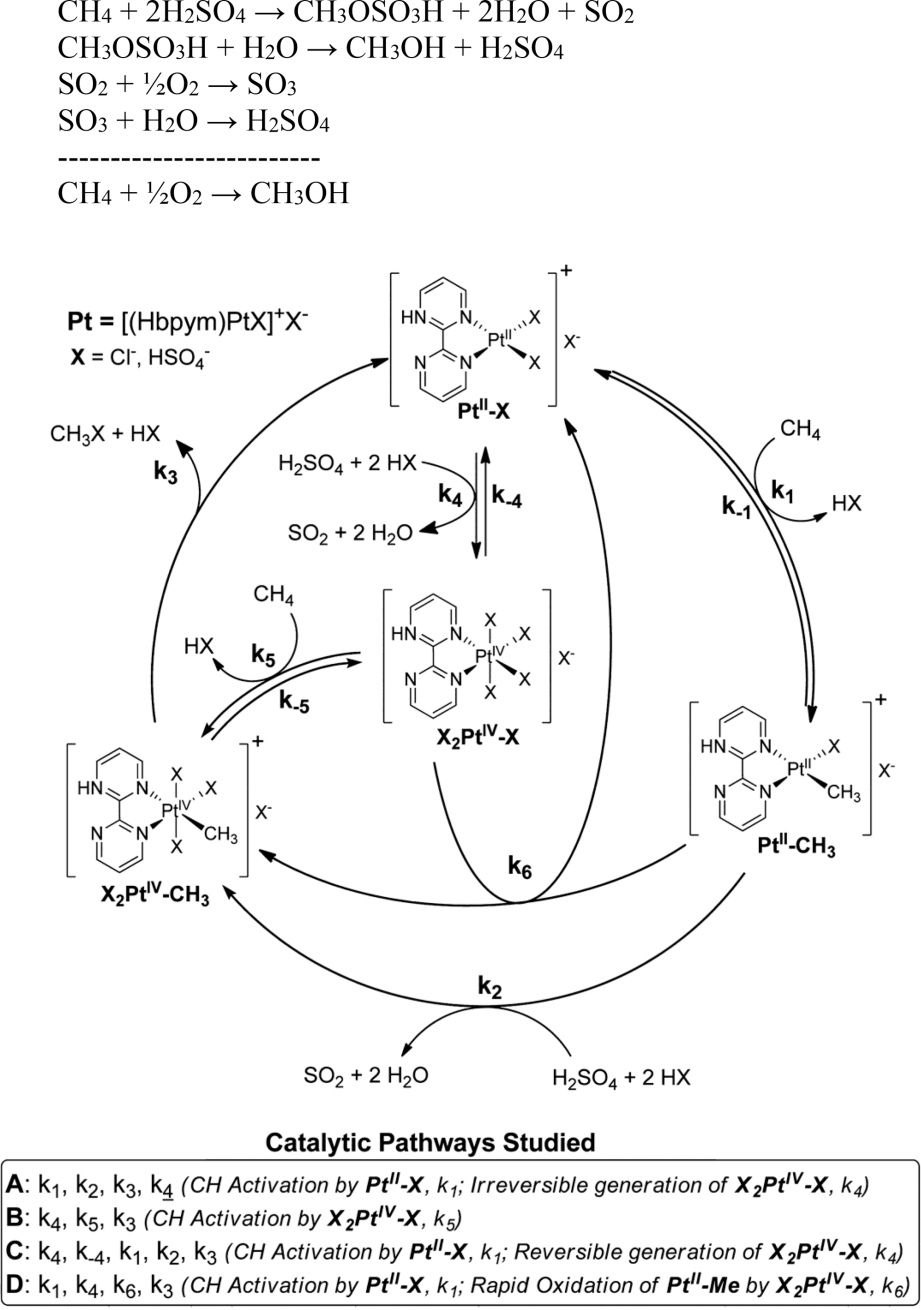
Plausible pathways for the Periana–Catalytica system that may account for the observed high stability. A postulated “self repair” mechanism is shown that returns X_2_Pt^IV^-X species, which is inactive for C–H activation, to an active Pt^II^-X form. Reproduced with permission from ref (221). Copyright 2013 American Chemical Society.

The best known example is Periana’s Pt^II^(bpym)Cl_2_ system (bpym = 2,2′-bipyrimidinyl), which catalyzes the oxidation of methane in highly concentrated sulfuric acid to methyl bisulfate (CH_3_OSO_3_H) and protonated methanol ([CH_3_OH_2_]^+^). This process has a reported selectivity to methanol derivatives of up to 81% at ∼90% methane conversion (batch experiment starting with 102% oleum as both reaction solvent and oxidizing agent at 220 °C under 34.4 bar methane).^220^ Following electrophilic CH activation to form a Pt^II^–CH_3_ species, simplified mechanisms describe oxidation to Pt^IV^–CH_3_ by sulfuric acid and reductive elimination/functionalization to liberate methyl bisulfate and regenerate the active Pt^II^ catalyst (Figure 13).^217^ This would need to be integrated with separate process steps for hydrolysis of methyl bisulfate to methanol and for oxidation of SO_2_ with air to give an overall methane to methanol process:

These authors estimated that their intermediate products are more than 2 orders of magnitude less reactive than methane in their system due to the electron withdrawing effects of the ^–^OSO_3_H or ^+^OH_2_ substituents.^217^

The Pt(bpym)Cl_2_ catalyst has good solubility in the reaction system and reported volumetric productivities are within an industrially relevant regime for homogeneous catalysis. For example, an estimated 3.6 mol L^–1^ h^–1^ productivity compares with 15–40 mol L^–1^ h^–1^ for real industrial carbonylation processes.^222^ The corresponding turnover frequency (TOF) is ∼10^–3^ s^–1^, and the turnover number (TON) is around 300 without observed loss of activity. However, the reaction is strongly inhibited by product water (TOF drops to ∼10^–5^ s^–1^ at 90% H_2_SO_4_), therefore practical rates and conversions would require Pt catalyst inventories that are two to 3 orders of magnitude above affordable levels.^223^

Simple chloroplatinic salts were the basis for Shilov’s early, pioneering work on methane activation that inspired this field. Here, the Pt^II^ salt, K_2_PtCl_4_, catalyzes methane conversion to CH_3_Cl and CH_3_OH using a Pt^IV^ salt (K_2_PtCl_6_) as a theoretically regenerable and recyclable oxidizing agent (eq 3):
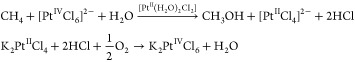
3However, this system suffers from a number of issues including low TOFs (<10^–5^ s^–1^ at 100 °C) and decomposition to inactive Pt black (TON < 20).^217^ More recently, the use of inorganic Pt salts has been re-examined in an H_2_SO_4_/SO_3_ solvent and oxidizing agent system similar to Periana’s,^224^ but seeking to optimize the catalyst activity by using excess SO_3_ concentrations. At low catalyst concentrations (partly to avoid solubility and stability issues) and high SO_3_ concentrations (i.e., up to 65% oleum), remarkably high TOFs were reported with high selectivity to methyl bisulfate and good catalyst stability (TON at least 650–940). For K_2_PtCl_4_ (600 μM) in 20% oleum at 215 °C, the derived TOF exceeded 25000 h^–1^ with >98% selectivity to methanol derivatives. Despite the low catalyst concentrations, reported volumetric productivities are as high as ∼16 mol L^–1^ h^–1^, well within a practical regime, and catalyst concentrations were around 2 orders of magnitude lower than for the Periana system. However, as Periana and colleagues point out,^217^ the use of excess SO_3_ (in oleum) is highly problematic because of its reaction with product water to give a net conversion to sulfuric acid, which is practically and economically irreversible.

Although publications in this area invariably consider catalyst stability in the reaction environment, they rarely address its disengagement from the reactor effluent for recycle to the reactor, as would be necessary for a continuous flow process configuration. Catalyst stability, or its regeneration, through product workup, separation, and recycle is equally important, and this is where commercial processes using homogeneous catalysts can suffer catalyst losses. One approach to avoiding this issue is to anchor the organometallic catalyst onto a solid support, and this has been demonstrated using a polymer of 2,6-dicyanopyridine with accessible bipyridyl units to coordinate platinum in an analogous way to Periana’s system.^225^ Batch reactions carried out in excess SO_3_ (see issue above) gave similar turnover frequencies to the homogeneous Periana catalyst at 215 °C, and this was apparently stable over multiple runs. Although encouraging, Pt inventory remains high, and the extremely low levels of Pt leaching that would be required for a practical system have not been confirmed under continuous flow reaction conditions. Furthermore, all systems where methyl bisulfate is the direct product require addition of excess water to release the desired methanol product and will incur substantial costs associated with subsequent removal of this water to regenerate concentrated sulfuric acid for recycle to the primary reaction system.

#### 4.1.2 Methane to Acetic Acid

An alternative approach to forming protected methyl ester intermediates is to form commercially relevant methanol derivatives directly, notably acetic acid, which is significantly more resistant to further oxidation than methanol. It is also worth noting that, as discussed in section 1.2, fully selective oxidation of methane to methanol with molecular oxygen requires both oxygens in the O_2_ molecule to be placed into the product,^226^ which is reminiscent of “dioxygenase” biological systems. In contrast, selective oxidation of methane to acetic acid coproduces water, accounting for half of the oxygen consumed (as in “monooxygenase” systems,^227^ see section 2.2) and potentially offering more feasible mechanisms for oxygen activation.

**Figure 14:**
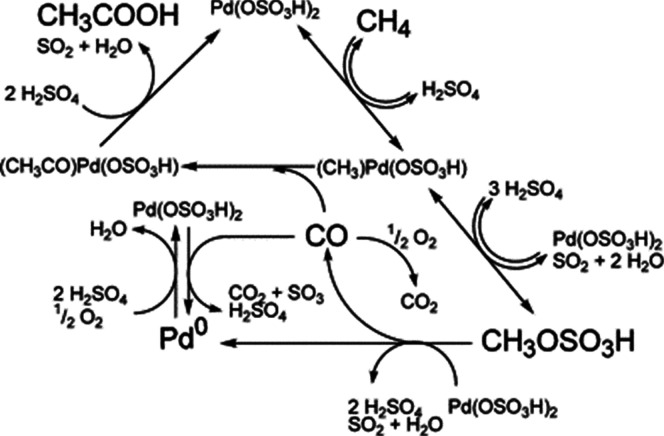
Proposed reaction mechanism for methane to acetic acid with Pd^2+^ catalyst in 96% H_2_SO_4_. Reproduced with permission from ref (229). Copyright 2006 Elsevier.

Direct methane to acetic acid has been demonstrated using a Pd(SO_4_)_2_ catalyst in 96% sulfuric acid (∼12% oxygenate yield, selectivity of 72% C-mol acetic acid, and 17% methanol equivalent, TOF ∼ 10^–3^ s^–1^ at 180 °C), with ^13^C labeling confirming that both the methyl and carbonyl components of the product originate from methane (eq 4):^228^
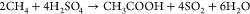
4The acetic acid is thought to be formed by reductive elimination from a Pd^II^-acyl intermediate, itself a result of the relatively facile insertion of CO resulting from over oxidation of methane (via methyl bisulfate) into a Pd^II^–CH_3_ bond. In this system, the Pd^0^ coproduced with acetic acid is returned to Pd^II^ by reaction with sulfuric acid, releasing SO_2_, which must then be separately reoxidized by O_2_ to SO_3_ to regenerate the sulfuric acid. However, the Pd^0^ → Pd^II^ oxidation step is not fully effective, and loss of active catalyst as insoluble Pd black appears to limit the lifetime of the catalyst system (TON < 20). Bell and co-workers have found that direct use of optimized molecular oxygen partial pressure in similar systems can lead to enhanced yields of acetic acid and near complete retention of Pd in solution (∼96% after 4 h with 200 psi CH_4_ and 125 psi O_2_ at 180 °C). Under these conditions, selectivity to acetic acid is 39% C-mol with 53% selectivity to CO; other significant products include CH_3_SO_3_H and CH_3_(SO_3_H)_2_.^229^ These authors described a somewhat different mechanism in which acetic acid is formed by the reaction of the Pd^II^-acyl species (actually (CH_3_CO)Pd(OSO_3_H)) with sulfuric acid, which retains the Pd^II^ oxidation state. Pd^0^ is formed during the oxidation of methyl bisulfate or CO intermediates and reoxidized to Pd^II^ by sulfuric acid and oxygen (Figure 14).

Yuan and co-workers^230^ have also shown oxidation of methane to acetic acid in the presence of molecular oxygen using K_2_PdCl_4_ catalyst in the presence of H_5_PMo_10_V_2_O_40_ (HPA) in trifluoroacetic acid (TFA) solvent. Very high selectivity to acetic acid was observed (>96%) at >10% methane conversion (80°C; 3.0 MPa CH_4_; 0.5 MPa O_2_) with TON > 4000 based on Pd. The HPA is thought to reoxidize Pd^0^ to Pd^II^ with molecular oxygen, but other aspects of the mechanism have not been established, especially the source of the carbonyl group in the acetic acid product.

Most industrial acetic acid is now manufactured by the carbonylation of methanol. Carbonylation of methane with CO as a coreagent, plus oxidant (strictly speaking carboxylation), could be of practical interest providing the CO is used efficiently. Low pressures of added CO enhance acetic acid formation in Periana’s Pd(SO_4_)_2_/sulfuric acid system^228^ and also in Bell’s approach using molecular oxygen,^229^ but higher CO partial pressures inhibit reaction, most likely due to the reduction of Pd^II^ to Pd^0^ by CO. Early work on methane carbonylation with CO over Pd(OAc)_2_ and/or Cu(OAc)_2_ catalysts in a TFA solvent, and with K_2_S_2_O_8_ as oxidant, showed clear catalysis of acetic acid formation with respect to the metal salts and that the persulfate could be replaced as a coreagent by molecular oxygen with over 400% yield of acetic acid based on Pd^231^ (80 °C, 20.3 bar CH_4_, 15.2 bar CO, 15.2 bar O_2_, 40 h). However, the very limited information on CO_2_ formation suggests that this is actually the main product, presumably from CO. This Pd/Cu acetate in the TFA/K_2_S_2_O_8_ system also catalyzed carboxylation of methane with CO_2_, although at very low rates. At about the same time, Sen and co-workers reported formation of acetic acid from methane, CO, and oxygen in an acidic (HCl) aqueous medium with RhCl_3_ catalyst and iodide promoter at around 100 °C,^232^ although rates were very low (TOF ∼ 0.1 h^–1^). Progressive substitution of water solvent by perfluorobutyric acid increased turnover dramatically (up to 2.9 h^–1^ at 80 °C in 6:1 (v/v) acid to water), but also switched the liquid products from acetic acid to predominantly methanol/methyl ester.^233^ This was attributed to the competition between CO insertion into a L_*x*_Rh-CH_3_ bond (to form acyl intermediate and then acetic acid) and nucleophilic attack by perfluorobutyrate ions on the same species. This work also showed that methane is substantially more reactive in this system than methanol or methyl ester. Subsequent DFT studies of mechanism support the proposal of methane C–H bond activation by [Rh(CO)_2_I_2_]^−^ through either an oxidative addition or σ-bond metathesis process, giving [Rh(CO)_2_I(CH_3_)]^−^ or [Rh(CO)_2_I_2_(H)CH_3_]^−^ complexes.^234^ As before, these Rh-CH_3_ species are either hydrolyzed to form methanol/methanol derivatives or undergo CO insertion to form acyl intermediates which are then hydrolyzed to release acetic acid. In both cases, the hydrolysis step produces [Rh(CO)_2_IH]^−^ which is oxidized by O_2_ via peroxo and hydroxo complexes to regenerate [Rh(CO)_2_I_2_]^−^.

Labeling studies reported by Sen and co-workers show that very little methane is converted to CO_2_ in their system,^233^ but CO_2_ formed from the added CO is not quantified. In a similar system using TFA/water instead of perfluorobutyric acid/water solvent, Chepaikin and co-workers also show tunability of products between acetic acid and methanol/methyl ester as a function of acid strength, as well as generally higher rates (e.g., TOFs of 71, 9, and 12 h^–1^ for methanol/methyl ester, acetic acid, and formic acid products, respectively, with RhCl_3_/NaCl/KI in TFA/water solvent at 95 °C, 6 MPa CH_4_, 1.84 MPa CO, 0.58 MPa O_2_).^235^ Importantly, these authors also quantify CO_2_ formation (from CO), which is always more than an order of magnitude greater than liquid products from methane, showing that neither CO nor O_2_ are used efficiently. Indeed, these authors propose mechanisms for methane activation involving Rh oxo or peroxo complexes formed in the presence of hypoiodous acid (HOI) itself derived from co-oxidation of HI and CO (i.e., HI + O_2_ + CO → HOI + CO_2_).^219^

#### 4.1.3 Overview

**Figure 15:**
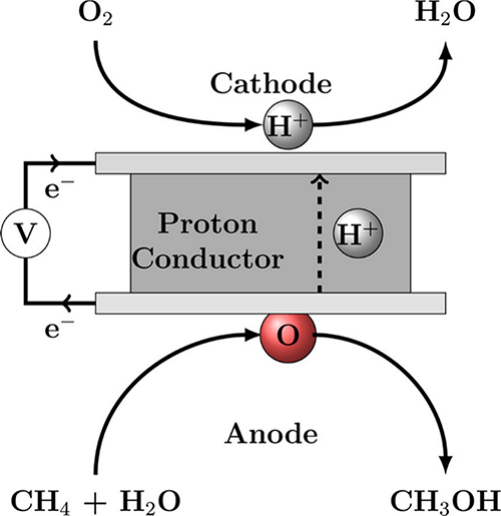
Schematic of an electrochemical cell for the overall conversion of methane to methanol. The anode reaction is CH_4_(g) + H_2_O(g) → CH_3_OH(g) + 2H^+^ + 2e^–^, and the cathode reaction is ^1^/_2_O_2_(g) + 2H^+^ + 2e^–^ → H_2_O(g). Reproduced with permission from ref (238). Copyright 2016 American Chemical Society.

In summary, work with homogeneous catalysts has provided important insights into mechanisms and strategies for methane functionalization, as discussed much more comprehensively in the reviews referenced above.^217,219^ However, to date, these do not provide a basis for the development of practical processes. One interesting new direction could be combination with electrochemistry (Figure 15). The continuous oxidation of methane to methanol has recently been demonstrated using a Shilov-like catalyst system but driving the reoxidation of Pt^II^ to the Pt^IV^ oxidizing agent electrochemically, equivalent to equation A above.^236,237^

Using a Pt anode, a moderately acidic electrolyte (0.5 M H_2_SO_4_/10 mM NaCl), 10 mM Pt^II^/Pt^IV^ chloroplatinic salts, and careful modulation of the cell current to control the Pt^II^/Pt^IV^ ratio (∼30% as Pt^II^), methane (500 psi) was slowly oxidized to methanol and methyl chloride at 130 °C.^236^ This model system used vanadyl sulfate as a sacrificial oxidant at the cathode but could be readily combined with O_2_ reduction. Reaction rates were extremely low (TOF ∼ 0.3 h^–1^), and although initial selectivity to CH_3_OH(Cl) was high at ∼96%, overoxidation was clearly significant at longer reaction times (∼82% CH_3_OH(Cl) selectivity at ∼9 overall TON). Furthermore, electrode current densities were orders of magnitude below practical levels.^238^ Nevertheless, points of interest include inhibition of inactive Pt^0^ formation and the controllability of the Pt^II^/Pt^IV^ ratio, and this may be a fruitful area for future research. An electrochemical approach has also been reported for the electro-oxidation of Pd^II^SO_4_ to a Pd^III^ dimer (Pd^III^_2_) in fuming sulfuric acid, which then activates methane at remarkably high rates.^239,240^ The reported TOF of ∼2000 h^–1^ at 140 °C under 500 psi CH_4_ is approximately 5000 times higher than for conventional catalysis with Pt^II^SO_4_ under similar conditions. An unconventional mechanism has been proposed^241^ in which the Pd^III^_2_ species abstracts hydrogen from methane to form a CH_3_^•^ radical and reduced Pd_2_^II,III^ dimer. The CH_3_^•^ radical can then recombine with the Pd_2_^II,III^ dimer to form a CH_3_Pd^III^_2_ species which liberates methyl bisulfate by reductive elimination; this process is stoichiometric in Pd^III^_2_ overall. Alternatively, the CH_3_^•^ radical can initiate a radical chain reaction with SO_3_ forming methanesulfonic acid, which is generally the major product (eq 5 and 6).
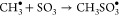
5
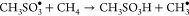
6As before, where excess SO_3_ is used, hydrolysis of products to release methanol leads to a net conversion of SO_3_ to sulfuric acid, and as a result product separations and recycles would be highly challenging. However, this work demonstrates how electrocatalysis can access novel species, and perhaps mechanisms, with potentially enhanced reactivity, and is now an active area of research. We recommend a recent article from Roman-Leshkov and colleagues^242^ for a broad review of electrocatalytic approaches to methane activation encompassing both homogeneous and heterogeneous catalysis, including a manifesto for future research.

### 4.2 Metal Nanoparticles

#### 4.2.1 Supported AuPd Catalysts with H_2_O_2_ as Oxidant

**Figure 16:**
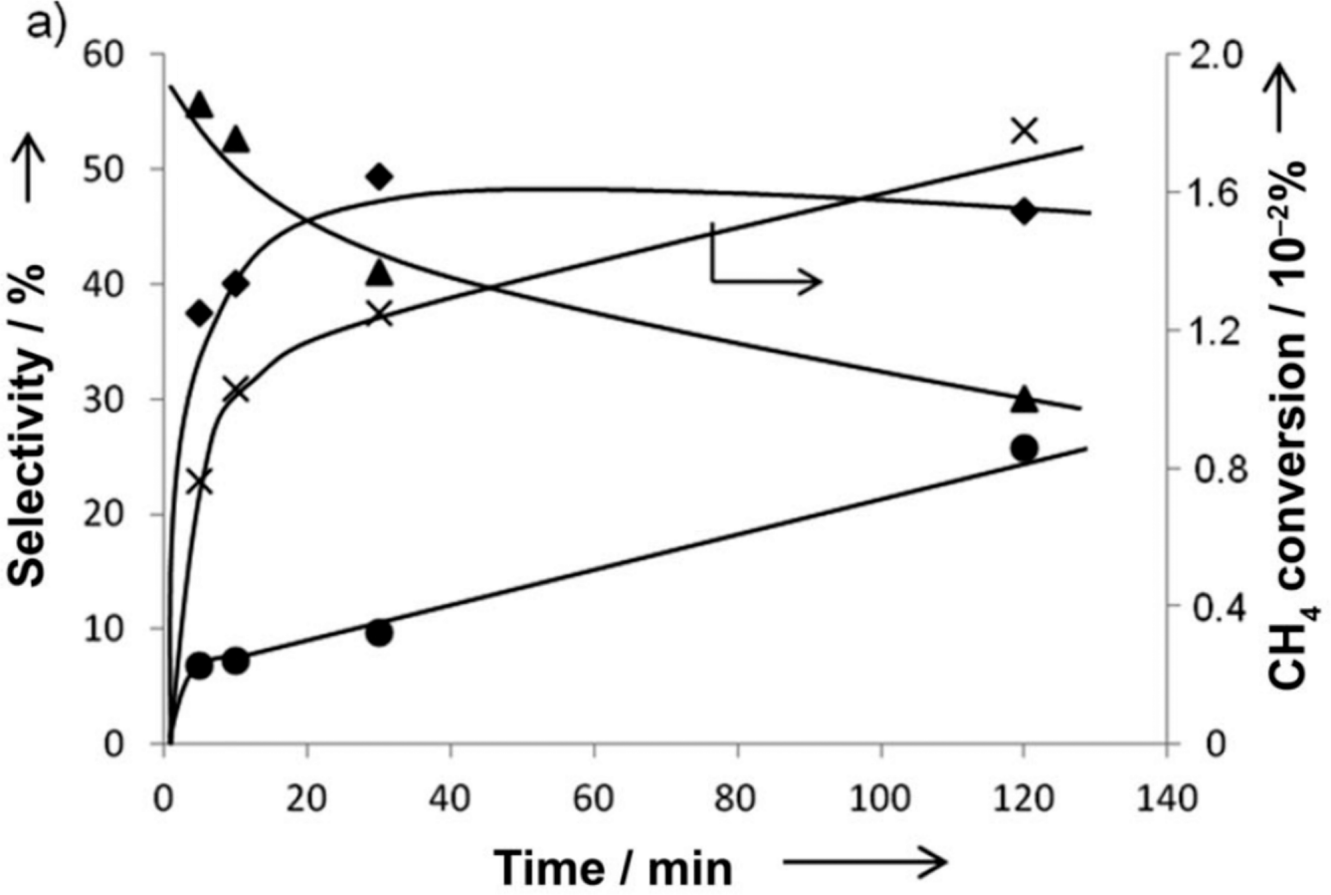
Selectivity as a function of time for methane oxidation using H_2_O_2_ as oxidant in the presence of a 1 wt % AuPd/TiO_2_ catalyst prepared by incipient wetness. Symbols: methyl hydroperoxide (▲), methanol (◆), CO_2_ (●) and methane conversion (crosses). Reaction conditions: *P*_(CH4)_ = 30.5 bar, [H_2_O_2_] = 0.5 M, *T* = 50 °C, stirring rate = 1500 rpm, and catalyst mass = 10 mg. Reproduced with permission from ref (250). Copyright 2012 Wiley-VCH.

A low temperature alternative route to the selective oxidation of methane has focused on the use of AuPd alloys. Indeed, the formation of H_2_O_2_ from molecular H_2_ and O_2_ has been well studied over AuPd surfaces,^243−245^ in addition to the selective activation of primary C–H bonds.^246,247^ Both reactions have been linked through the formation of intermediate hydroperoxyl and hydroperoxy species. The activation of CH_4_ by H_2_O_2_ is widely considered to proceed through a radical mechanism, in which ^•^OH radicals generated over AuPd surfaces are key in the activation of the C–H bond through hydrogen abstraction, widely considered to be the rate limiting step.^248^ Ab Rahim et al.^249^ found that the first oxidation product observed is methyl hydroperoxide, which is gradually converted into methanol (Figure 16). Using electron paramagnetic resonance (EPR) spectroscopy, they also established the formation of both methyl and hydroxyl radical species during the selective oxidation of methane, a key observation. This suggests that while methane oxidation over AuPd surfaces, with H_2_O_2_ as an oxidant, shares a common first product with that over Cu and Fe containing zeolites, the radical intermediates differ.

**Figure 17:**
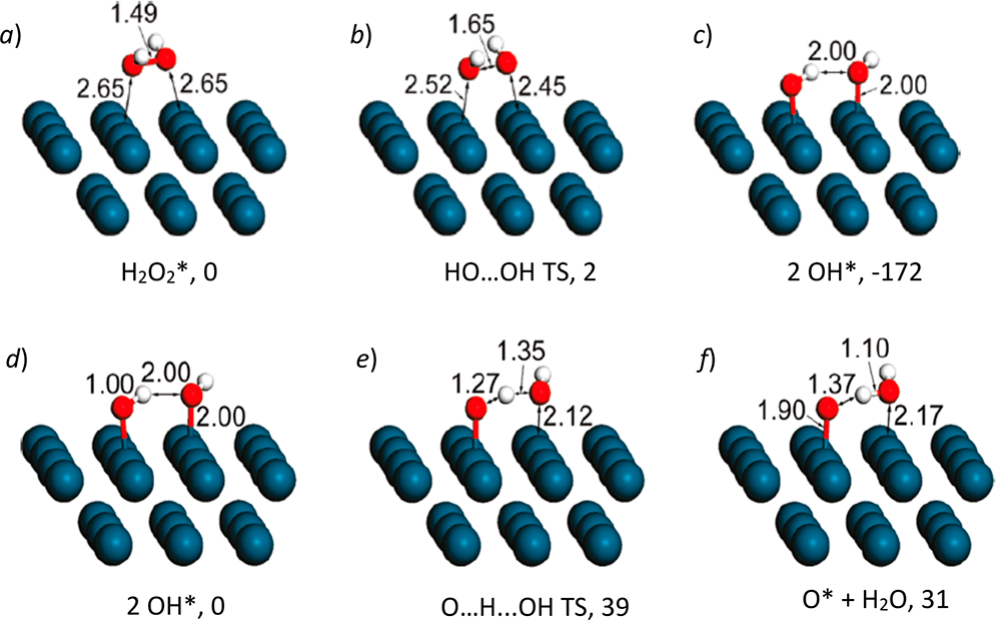
Calculated structures and energies for H_2_O_2_ decomposition (a–c) and the disproportionation of the resulting surface OH* groups (d–f). Energies relative to intermediates (*a* for a–c and *d* for d–f) in kJ mol^–1^ given underneath graphics, distances indicated on graphics in Å, * indicates an adsorbed species. Atom colors: Pd, blue; O, red; H, white. PBE simulations carried out with a periodic slab model four layers thick, only upper two levels are shown. Adapted with permission from ref (252). Copyright 2011 American Chemical Society.

In particular, in the zeolite case, methyl radicals could not be seen by EPR spectroscopy.^251^ So for AuPd/TiO_2_ a credible mechanism follows the sequence:
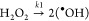
7
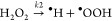
8
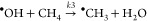
9
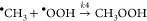
10
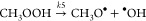
11
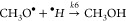
12The mechanism laid out in eqs 7–12 shows the pathway for radicals derived from H_2_O_2_ to activate methane and to give the first oxidation products seen in the AuPd/TiO_2_ catalyzed reactions. We note that, in contrast to HOO^•^, H^•^ and CH_3_O^•^ radicals are both highly reactive, meaning that the production of methyl hydroperoxide through (eq 10) will occur in preference to the direct reaction to methanol through (eq 12). However, the same radicals can also recombine to give water as has been well studied in experiments with similar catalysts used in the in situ synthesis of H_2_O_2_ from hydrogen and oxygen.^243^ To achieve high H_2_O_2_ utilization, there is a need to inhibit this H_2_O_2_ self-decomposition, particularly given the relatively high costs of commercial H_2_O_2_ and the first-order dependence of H_2_O_2_ decomposition on the concentration of the oxidant. Indeed, a comprehensive multivariance analysis by Serra-Maia et al.^248^ has outlined many of the critical factors responsible for the unselective decomposition of H_2_O_2_ and has shown this to be favored over Pd-rich and larger AuPd nanoparticles. The problem of H_2_O_2_ decomposition has also been highlighted by computational work. For example, Yoshizawa and co-workers^252^ have used DFT calculations with the gradient corrected functions of Perdew, Burke, and Ernzerhof (PBE)^253^ to look at the adsorption and decomposition of H_2_O_2_ on Pd(111) surfaces. Figure 17 shows the resulting structures and calculated energies.

The dissociation of H_2_O_2_ is strongly exothermic, with a calculated change in energy between molecularly adsorbed H_2_O_2_* and two surface OH* groups of −172 kJ mol^–1^. The surface OH* groups are stabilized by a hydrogen bonding interaction, which also orients the groups to allow the proton transfer required for disproportionation to O* and H_2_O. The disproportionation process is endothermic with respect to 2OH* and has a more substantial barrier than seen for H_2_O_2_ decomposition. However, this may become more favorable if the full aqueous solvent of the experimental conditions were considered. In addition, the energetics for hydrogenation as a decomposition route was calculated, but this was found to be less favorable than disproportionation of 2 OH* and direct hydrogenation of adsorbed H_2_O_2_* could also be ruled out on energetic grounds.

The use of dispersion corrected density functional theory (PBE-D3) allows an estimate for the molecular adsorption energy of H_2_O_2_ to be obtained. Using this method and periodic slab models with five atomic layers, Nasrallah et al.^254^ obtained adsorption energies (*E*_ads_) of −45 kJ mol^–1^ and −47 kJ mol^–1^ for Au(111) and Au(100) surfaces. Molecular adsorption is more favorable on Pd surfaces (*E*_ads_(Pd(111)) = −61 kJ mol^–1^*E*_ads_(Pd(100)) = −62 kJ mol^–1^) with the exothermic dissociation to give 2OH*, also having a more negative reaction energy for Pd than Au; (Δ*E*_dis_(Pd(111)) = −188 kJ mol^–1^, cf. Δ*E*_dis_(Au(111)) = −98 kJ mol^–1^ and Δ*E*_dis_(Pd(100)) = −254 kJ mol^–1^, cf. Δ*E*_dis_(Au(100)) = −186 kJ mol^–1^). Notably, the reaction barrier for the dissociation is also much lower for Pd than for Au, with Pd(111) giving a vanishingly small barrier of 5 kJ mol^–1^ while even the more reactive of the Au surfaces, Au(100), having a barrier of 38 kJ mol^–1^.

The interaction of H_2_O_2_ with models of small nanoparticles have also been investigated with similar conclusions that the dissociation into 2 OH* is extremely facile with a calculated adsorption energy in this dissociated state of −237 kJ mol^–1^ at the base of a Au_10_ cluster formed from a (7,3) atom bilayer of close packed atoms.^255^ Interestingly, in the same paper, it is shown that dissociation of H_2_O_2_ on the rutile TiO_2_(110) takes place by proton donation to the surface to produce ^–^OOH* and a surface hydroxyl group.

While early studies by Ab Rahim et al. demonstrated the efficacy of supported AuPd catalysts to catalyze the selective oxidation of methane over AuPd/TiO_2_,^249,250^ more recently further investigations have focused on improving H_2_O_2_ utilization by limiting the decomposition of the oxidant at surface sites. This can be achieved through thermal pretreatment of the support, prior to immobilization of alloyed AuPd colloids, or exposure of the supported metal catalysts to high temperature oxidative heat treatments.^256^ The modification of the anatase/rutile ratio of the TiO_2_ support prior to catalyst preparation was found to be particularly effective in significantly improving the methane oxidation productivity compared to previous approaches that utilized analogous AuPd/TiO_2_ catalysts prepared by more conventional synthetic routes such as incipient wetness. Indeed, the TOF for the optimal AuPd/TiO_2_ (rutile) supported catalyst (103 h^–1^) was considerably greater than that observed over earlier TiO_2_ based materials (7 h^–1^) under identical reaction conditions while also offering higher oxygenate selectivity (90.7% and 85.4%, respectively).

Despite the promising selectivity observed over supported AuPd catalysts, product formation rates are still comparatively low. The incorporation of Cu^2+^ in Fe-based ZSM-5 catalysts is known to improve methane oxidation selectivity toward methyl hydroperoxide and methanol while suppressing further oxidation reactions.^257^ The incorporation of Cu^2+^ into supported AuPd catalysts was also found to increase methane oxidation rates by a factor of 5 compared to supported AuPd alone. In addition, catalytic H_2_O_2_ utilization was found to be inherently related to methanol synthesis rates, with nonselective H_2_O_2_ consumption increasing significantly in line with catalyst productivity.

The role of the support is far from simple, with several materials able to generate radical species when exposed to peroxides. For example, DFT calculations indicate that H_2_O_2_ adsorbed to the surface of hydroxylated rutile TiO_2_ will form surface bound OOH species.^255^ While experimentally TiO_2_ alone is unable to catalyze methane oxidation under the conditions typically used with AuPd/TiO_2_ catalysts,^250^ the involvement of oxygen species generated from H_2_O_2_ on the support cannot be ruled out, and it seems reasonable that the support mediated reactions may consume the H_2_O_2_ oxidant via competitive side reactions. Similarly, some supports are able to promote termination of radical chains and so can substantially modify the rate of oxygenate formation.^250,258^ Acidic supports are able to promote H_2_O_2_ stability through inhibition of H_2_O_2_ degradation pathways.^249,259,260^ Beneficial effects observed through reagent confinement in micropores for AuPd nanoparticles immobilized on aluminosilicates have also been observed. ZSM-5 supports in particular have been widely studied as supports for metal nanoparticles for the selective oxidation of methane.^144,261,262^ However, while these catalysts significantly outperform analogous materials supported on common oxides, the activity can often be largely attributed to the zeolite, and indeed both catalytic activity and H_2_O_2_ utilization has been found to be greater over the bare ZSM-5 compared to the AuPd loaded analogue.^263^

Alternative studies have set out to overcome limitations associated with low methane solubility, which inherently inhibits oxygenate production rates. In particular the high surface areas, pore structure, and tolerability of metal organic frameworks^264−267^ have led to their investigation as hosts for AuPd nanoparticles, resulting in high production rates.^268^ Perhaps unsurprisingly, these studies, which utilized both H_2_O_2_ and molecular O_2_ as the oxidant, identified the need to control the supply of reactive oxygen species, with methanol selectivity promoted and CO_2_ formation rates inhibited through the control of oxidant ratio.

#### 4.2.2 Colloidal Metal Nanoparticles As Catalysts for Methane Oxidation

Precious metal colloidal catalysts represent a burgeoning field of research, with the solution phase generation of metal nanoparticles offering a high degree of control over particle size, shape, and composition.^269^ Many of the unsupported colloidal systems utilized recently for selective oxidation of methane can trace their inspiration back to the early work on homogeneous catalysis systems from Periana and co-workers, who reported the activity of Hg and Pt complexes toward methanol and its derivatives.^220,270^ Jones et al.^271^ also demonstrated the ability of cationic Au or chelated Pd species to oxidize CH_4_.^272^ However, these earlier works relied on the utilization of high reaction temperatures and strongly acidic media, such as oleum, to facilitate the reaction. More benign systems that utilize H_2_O_2_ have been reported to offer reasonable activity toward CH_4_ activation. However, the utilization of complex solvent systems and concerns around the agglomeration and precipitation of homogeneous catalysts has been a major limitation.^273−275^ Recent studies have identified the ability of ligand stabilizers to enhance the selectivity of colloidal AuPd species toward H_2_O_2_,^276,277^ presumably through inhibition of H_2_O_2_ diffusion to the metal surfaces and control of the rate of hydroxyl radical production. In a similar manner, the addition of such stabilizers to supported metal catalysts has also been demonstrated to improve product selectivity. However, selective H_2_O_2_ utilization is far greater over unsupported colloidal analogues than for the supported systems even with the use of stabilizers. This could be as a result of the formation of highly faceted alloy particles and an extended metal–support interface which promotes H_2_O_2_ decomposition pathways. These features are absent in the unsupported colloidal systems. As the colloidal stabilizers are themselves organic molecules that could be oxidized, the choice of stabilizer is an important factor in catalyst design. Poly(vinyl)alcohol (PVA) for example yields appreciable concentrations of methanol in a blank reaction with catalyst and oxidant but in the absence of methane. By way of contrast, poly(vinylpyrrolidone) (PVP) does not readily decompose to methane oxidation products under the mild reaction conditions used for H_2_O_2_ driven methane oxidation.^278^

**Figure 18:**
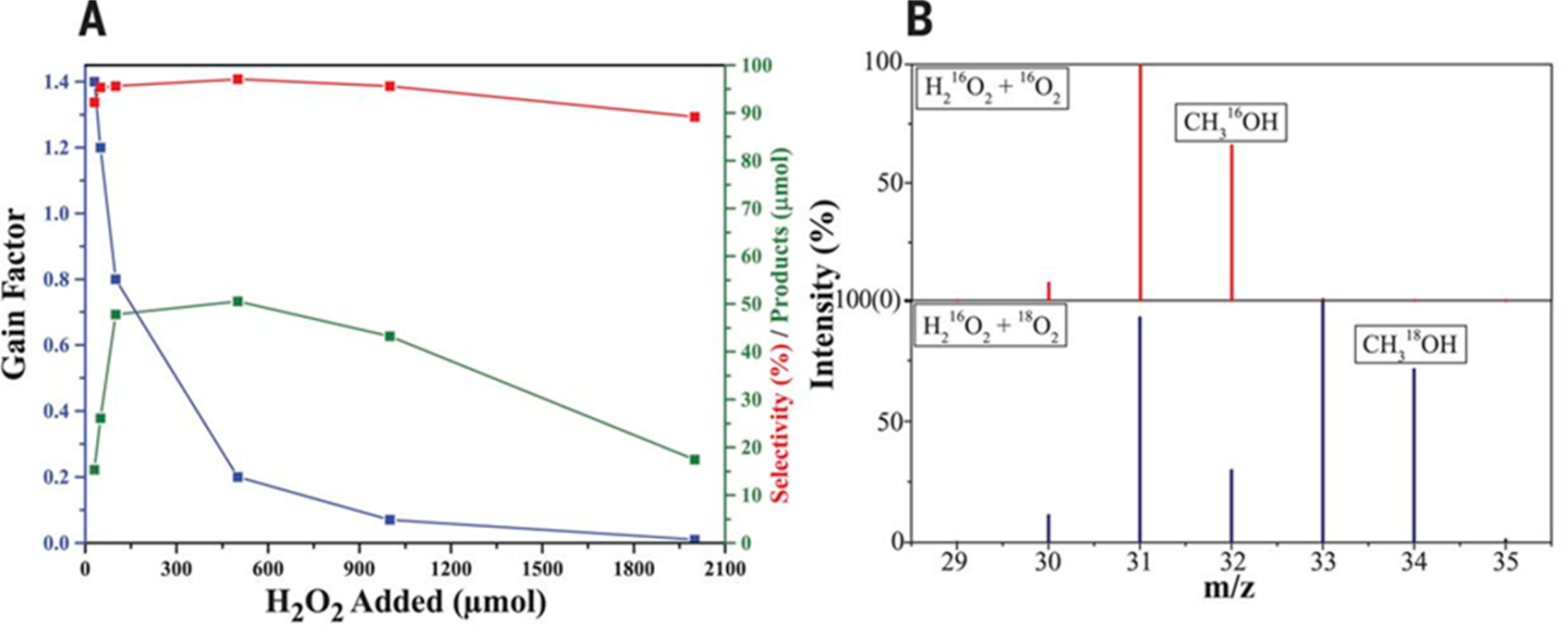
Methane oxidation reactions carried out over unsupported Au–Pd colloids. (A) Gain factor (blue), selectivity (red), and total amount of products (green) as a function of the different amounts of H_2_O_2_ used. (B) GC-MS spectra of CH_3_OH formed (mass = 32 and 34 for CH_3_^16^OH and CH_3_^18^OH, respectively) during methane oxidation with a Au–Pd colloid via H_2_^16^O_2_ + ^16^O_2_ (upper spectrum) or H_2_^16^O_2_ + ^18^O_2_ (lower spectrum). For CH_4_ oxidation with ^18^O_2_, >70% of ^18^O_2_ molecules were incorporated in the CH_3_OH product. *m*/*z*, mass/charge ratio. Adapted with permission from ref (278). Copyright 2017 AAAS.

The Au:Pd ratio has also been shown to affect H_2_O_2_ utilization and in turn catalytic activity toward methane oxidation.^279,280^ It should be noted that the activity of such colloidal AuPd nanoparticles has been found to compare favorably to both methane monooxygenase (MMO) and Fe-Cu/ZSM-5 catalysts,^281^ although in the case of MMO molecular oxygen is utilized as the terminal oxidant with clear advantages over H_2_O_2_. Agarwal et al.^278^ have demonstrated that the application of AuPd colloids, prepared with PVP in conjunction with low concentrations of H_2_O_2_, can initiate the incorporation of a significant fraction (70%) of gas phase O_2_, as evidenced by ^18^O_2_ labeling experiments (Figure 18). Notably, this represented the first example of the low temperature selective oxidation of methane to methanol, where molecular oxygen was demonstrably incorporated into the partial oxidation product stream. Perhaps inspired by this earlier work, Xu et al. have recently investigated the utilization of a combination of H_2_O_2_ and molecular O_2_ oxidants over MOF (ZIF-8) encapsulated AuPd nanoparticles.^268^ The combination of H_2_O_2_ and O_2_ was found to greatly outperform the use of either oxidant alone, particularly molecular O_2_, which when utilized in conjunction with the AuPd@ZIF-8 catalyst offered no activity toward methane activation. In agreement with Agarwal et al.^278^ isotopic labeling experiments revealed the incorporation of O_2_ into reaction products, which primarily consisted of CH_3_OOH at short reaction times when H_2_O_2_ was also present in the reaction mixture. With growing interest into the role of AuPd colloids for the selective oxidation of methane and based on numerous studies that have previously elucidated the efficacy of Pt clusters in stabilizing the intermediate methyl species,^282−284^ Chen et al. have recently demonstrated the high activity and selectivity that can be achieved over Pd@Pt core–shell colloids and further identified that the presence of Pd is crucial, acting as an electronic modifier for Pt sites.^285^

#### 4.2.3 In Situ H_2_O_2_ Generation for Methane Oxidation

While the selective oxidation of methane using commercially generated H_2_O_2_ may be interesting at the academic level, the economic and technical challenges associated with H_2_O_2_ formation via current industrial routes, dominated by the anthraquinone oxidation process, in addition to its safe transport and storage, is likely to preclude the application of preformed H_2_O_2_ at an industrial level. This is especially the case when considering the cost of H_2_O_2_ can exceed that of methanol. While substantial savings can be achieved through improved utilization of H_2_O_2_, the selective partial oxidation of methane via in situ production of H_2_O_2_ from molecular H_2_ and O_2_ would offer an attractive alternative and substantially reduce costs associated with the oxidant.

Initial studies by Sen and co-workers, which utilized gaseous mixtures of CO/H_2_O/O_2_ in order to generate H_2_O_2_ over homogeneous Pd^286,287^ and Rh^233^ systems, via a metal catalyzed water gas shift reaction, clearly demonstrated the efficacy of the in situ route.^286^ However, this approach was limited by (i) The homogeneous nature of the catalyst, (ii) the choice of solvent, utilizing aqueous solutions of perfluorobutyric or trifluororacetic acid, which ultimately resulted in the formation of complex product mixtures. amd (iii) the requirement of several equivalents of halide (notably chloride or iodide) to maintain the stability of the homogeneous catalysts. Further studies, overcame concerns associated the formation of unwanted byproducts, such as acetone, through utilizing a heterogeneous Pd/C catalyst in addition to a Cu(II) salt.^286^ However, the need for a co-reductant, in this case carbon monoxide, in order to maintain high selectivity toward partially oxidized products again limited the industrial viability of the in situ approach.^287,288^

Subsequent seminal studies by Park and co-workers building on the Pd–Cu system developed by Sen and co-workers reported the selective oxidation of methane in the liquid phase by utilizing H_2_O_2_ generated in situ from H_2_ and O_2_ over heterogeneous Pd catalysts in conjunction with homogeneous Cu or V cocatalytsts.^289,290^ Notably, unlike the Cu based system, the methane oxidation mechanism catalyzed by homogeneous V species did not appear to proceed via an hydroxyl radical-based route. Instead, it was proposed that the generation of the intermediate monoperoxomonovanadate species, formed through co-ordination of VO_2_^+^ with H_2_O_2_ can promote the activation of methane via hydrogen abstraction.^290^ Alternative studies by Fan et al. demonstrated that the homogeneous metal species used in previous works could be replaced with quinones, namely *p*-tetrachlorobenzoquinone (TCQ) in order to direct the oxidation of methane away from formic acid, formed in the absence of TCQ and instead toward methanol derivatives.^291^

More recent studies highlighting the high catalytic perfomance that could be achived through in situ H_2_O_2_ generation over supported Pd catalysts and subsequent methane actvation with Fe incorporated ZSM-5 have been reported.^292^ However, these works rely on the presence of relatively high concentrations of acid to both promote catalytic performance and inhibit metal leaching, which can lead to decreased reactor lifetime, through corrosion. Alternatively, V centered catalyts, including vanadyl oxysulfate (VOSO_4_)^293^ or heteropolyacid based Pd catalysts,^294^ have been reported to offer reasonable selectivity toward the low temperature oxidation of methane to formic acid, using either preformed H_2_O_2_ or H_2_O_2_ generated in situ. Min et al. demonstrated not only the key role of support acidity in promoting H_2_O_2_ stability but in a manner similar to that reported for homogeneous V species, the ability of V centers within the heteropolyacid Keggin structure to synthesize monoperoxomonovanadate species, which are ultimately responsible for methane activation.^294^

**Figure 19:**
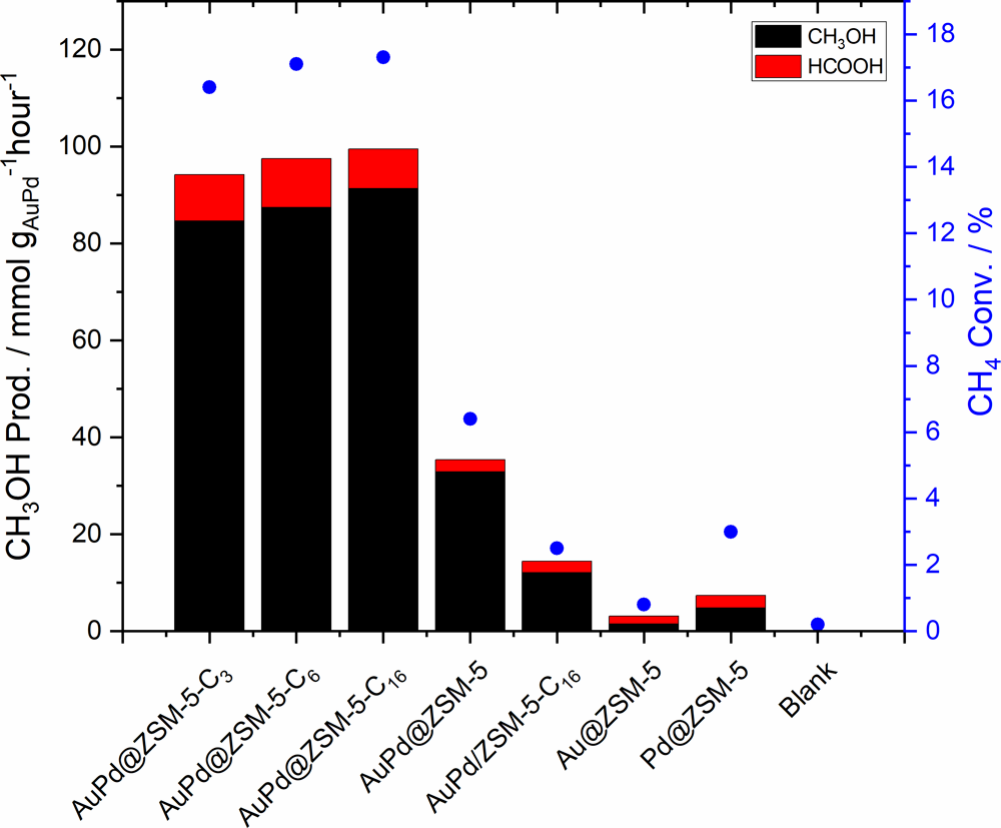
Oxidation of methane via the in situ generation of H_2_O_2_ from H_2_ and O_2_ over various AuPd@ZSM-5 catalysts. Histograms show methanol (black) and formic acid (red) productivities, blue points give methane conversion. Reaction conditions: 10 mL of water, 30 min, 70 °C, 27 mg of catalyst, feed gas at 3.0 MPa with 3.3% H_2_/6.6% O_2_/1.6% CH_4_/61.7% Ar/26.8% He, and 1200 rpm (rpm). Adapted with permission from ref (262). Copyright 2020 AAAS.

The utilization of H_2_O_2_, generated in situ from molecular H_2_ and O_2_ over AuPd surfaces has been well studied for the selective oxidation of methane. Indeed, the in situ route has been demonstrated to offer significantly improved selectivity toward methanol compared to the use of preformed H_2_O_2_,^250^ although productivities could still be considered somewhat limited regardless of the route to methane oxidation. Typically, far lower product formation rates have been reported through in situ H_2_O_2_ formation, compared to using preformed H_2_O_2_ directly. This is likely a result of the low concentrations of H_2_O_2_ present near catalytically active sites.^295^ To overcome H_2_O_2_ diffusion limitations Jin et al.^262^ have modified the external surface of AuPd@ZSM-5, where AuPd alloys are incorporated within the aluminosilicate framework, with an organosilane layer. The hydrophobic layer was found to both promote the diffusion of reagents (H_2_, O_2_, and CH_4_) and to confine the generated H_2_O_2_ near the AuPd nanoparticles (Figure 19). Notably, the performance of the bimetallic alloy was found to greatly exceed that observed over the monometallic analogues, which may be unexpected given the numerous studies which report the synergistic enhancements that can be achieved through the formation of AuPd alloys. This approach represents a major step forward toward the application of direct methane activation, achieving high rates of methane conversion (17.3%) and methanol selectivity (92%). However, despite these impressive improvements in catalytic activity achieved over supported AuPd nanoparticles, there is still a need to increase product formation rates.

Alternative approaches to improve methanol production rate have focused on the introduction of Cu, a key catalytic species in the oxidation of methane using preformed H_2_O_2_, into supported AuPd catalysts. While a clear improvement was observed when utilizing commercial H_2_O_2_ as the oxidant, the addition of Cu was found to deleteriously affect catalytic activity toward the in situ oxidation of methane, with similar inhibitions in catalytic performance observed when evaluating these materials for the direct synthesis of H_2_O_2_. This is possibly indicative of Cu species blocking catalytic sites responsible for H_2_O_2_ production.^296^ However, more recent studies have suggested the inclusion of Cu at loadings far lower than those utilized in earlier works can significantly enhance H_2_O_2_ production rates, although investigation of these materials for in situ methane oxidation are yet to be reported.^297^

### 4.3 Microporous Materials in Aqueous Media

The efficacy of methane monooxygenase at comparable reaction conditions to those utilized for the chemocatalyst mediated oxidation of methane in the liquid phase (water solvent, 50 °C) is well-known (section 2.2). The active site in the enzyme system is based on diiron clusters, and so, inspired by these biological routes, numerous studies have set out to investigate the low temperature oxidation of methane. The use of aqueous media and low temperature broadens the range of oxidants that can be used, and H_2_O_2_ has been a popular choice because the oxidation side product is limited to water (CH_4_ + H_2_O_2_ = CH_3_OH + H_2_O). Indeed, Sorokin and co-workers^298,299^ have reported the activation of methane using H_2_O_2_ under comparably mild conditions, using a μ-nitrido iron phthalocyanine complex immobilized on a silica carrier, with the use of a mildly acidic reaction media promoting catalytic activity. However, according to Forde et al.,^300^ this type of catalyst has relatively low activity (i.e., the TOF was around 2 h^–1^) and is found not to be stable under methane oxidation reaction conditions.

Many researchers have drawn analogies between the restricted space of an enzyme active site and the well-defined pore space of inorganic microporous materials such as zeolites.^144,251^ Accordingly, the use of metal exchanged zeolites for direct methane activation has attracted considerable attention, with iron containing systems center stage.

#### 4.3.1 Fe/ZSM-5 with H_2_O_2_

**Figure 20:**
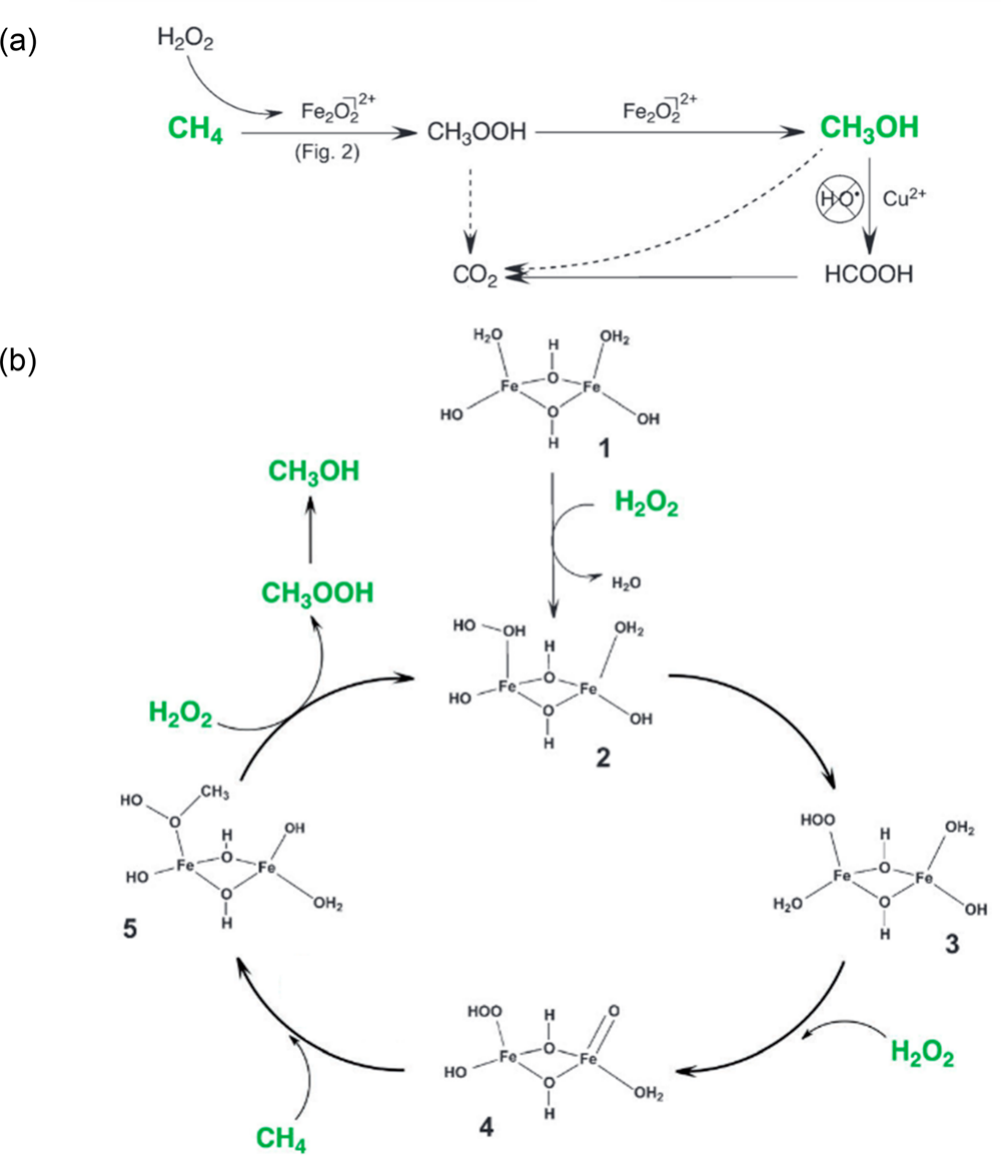
(a) A potential reaction scheme for the oxidation of methane proposed by Hammond et al.^144^ Methanol is formed through the conversion of the methyl hydroperoxide intermediate over the Fe sites present in the catalyst. ^•^OH radicals produced during the reaction are later responsible for the overoxidation of methanol. (b) The catalytic cycle for the oxidation of methane to CH_3_OOH using H_2_O_2_, catalyzed by a binuclear Fe species in ZSM-5, proposed by Hammond et al.^11^ The overall charge in each case is formally +2 as the species act as an extra-framework cation within the zeolite. Adapted with permission from ref (144). Copyright 2012 Wiley-VCH.

Hutchings and colleagues^144^ demonstrated that commercial ZSM-5 can be very effective in converting methane to oxygenates in aqueous media with H_2_O_2_ as the oxidant. They report that a methane conversion of 0.3% and oxygenate selectivity of 95% (i.e., 77.1 mmol of oxygenates) were obtained with 27 mg of ZSM-5 catalysts under a typical batch reaction at 50 °C for 30 min, with 10 mL of water, 0.5 M H_2_O_2_, and 30.5 bar of CH_4_. A key step in the catalyst preparation was found to be calcination at 550 °C for 3 h, prior to use. Although the main product generated was formic acid (HCOOH) with about 54% selectivity, detailed time-online studies revealed that the initial primary product was methyl hydroperoxide (CH_3_OOH). The selectivity toward HCOOH increased with a longer reaction time, suggesting that HCOOH is at least partly formed by the consecutive oxidation of CH_3_OOH and CH_3_OH. While other reactions may also coexist (e.g., HCOOH directly formed from CH_4_ oxidation^251^), the major reaction route was believed to proceed as shown in Figure 20a). In this scheme, CH_4_ is initially oxidized to CH_3_OOH, which subsequently reacts to form CH_3_OH that then is sequentially oxidized to HCOOH and finally CO_2_. Al-Shihri et al.^301,302^ later reported the detection of formaldehyde (H_2_CO) and its hydrated form (H_2_C(OH)_2_), which could also be short-lived intermediates that will quickly be oxidized to HCOOH.

Further detailed investigation demonstrated that this reaction followed a mechanism that had not been reported at the time.^144,251,303−305^ Fe impurities at a level of 140 ppm were found to be the active species in the commercial ZSM-5. Based on the Fe content, the TOF was estimated to be >2200 h^–1^ for a standard 30 min reaction, but the TOF was more than 14000 h^–1^ in the initial reaction period (2 min). These values were up to 3 orders of magnitude greater than any chemical systems reported prior to that point. EPR radical trapping experiments detected only oxygen-based radicals but not carbon-based ones, which suggested that the current process works differently to the established mechanisms such as α-oxygen or Fenton’s chemistry. In the N_2_O-based gas phase reaction discussed in section 3.2 in which the N_2_O is used to prepare α-oxygen species prior to reaction with methane, a high-temperature pretreatment (>800 °C) is typically needed for the (auto)reduction of Fe^3+^ to Fe^2+^. However, the use of materials prepared in this way was found to be detrimental to the activity of ZSM-5 catalysts used in the aqueous process because H_2_O_2_ decomposition was also catalyzed by the resulting agglomerated iron oxide species.^303^

Various spectroscopy techniques, electron microscopy studies, and density functional theory (DFT) calculations were used to further identify the active species for the Fe/ZSM-5 with H_2_O_2_ system. These have identified extra-framework diiron [Fe_2_(μ_2_-OH)_2_(OH)_2_(H_2_O)_2_]^2+^ species, containing antiferromagnetically coupled high-spin octahedral Fe^3+^ centers.^251^ A molecular-level mechanism was proposed (Figure 20b) using DFT simulations. The diiron site (1) first coordinates with H_2_O_2_ through the exchange of a water ligand to give species (2), H^+^ transfer and solvent rearrangement together give the species (3), which is formally a Fe^4+^/Fe^2+^ dimer, a second H_2_O/H_2_O_2_ then place H_2_O_2_ at the Fe^2+^ site. The formation of the Fe^4+^=O adjacent to a Fe-OOH site results in a novel, bifunctional oxidation center (4). The methyl radicals resulting from the C–H bond activation by the iron oxo group are immediately captured by the hydroperoxyl ligand at the second Fe center. The active site is regenerated after releasing the methyl hydroperoxide into the solution, closing the catalytic cycle. An H_2_O_2_/product stoichiometry of 2:1 is expected from the mechanism described above. Kinetics studies showed that under the standard condition (which uses an excess amount of CH_4_), only H_2_O_2_, but not CH_4_, is involved in the rate-determining step. A 61 kJ mol^–1^ activation energy was experimentally observed, which is of similar magnitude to, but lower than that measured for the soluble methane monooxygenase and is close to the theoretically predicted value.^144,251^

More active catalysts than the “bare” commercial ZSM-5 can be prepared by incorporating an additional amount of Fe onto ZSM-5 or Silicalite-1 support during or after the zeolite synthesis. However, similar approaches did not work for other types of zeolites such as β, Y, or ferrierite, indicating that the MFI framework played a critical role in the catalysis of this reaction. It was suggested that the Brønsted acidic sites induced by Al^3+^ or other trivalent cations (e.g., Ga^3+^) in the zeolite are indeed important for accommodating and dispersing the active Fe species. However, too low of a Si/Al ratio in ZSM-5 (e.g., 12.4^306^) was found to be detrimental, possibly due to unproductive decomposition of H_2_O_2_ by the acidic sites or agglomerated iron oxide particles. Likewise, high Fe loadings (e.g., 2.5 wt %) can also lead to a significant H_2_O_2_ decomposition, way beyond the 2:1 stoichiometry ratio needed for the selective oxidation reaction, possibly due to the presence of α-Fe_2_O_3_ particles.

The decomposition of H_2_O_2_ and MeOOH can both lead to the release of ^•^OH radicals, which was proposed to be the key reason for the formation of overoxidized products. This was supported by the observation that the MeOH selectivity was significantly improved for the ZSM-5 catalyst (i.e., increasing from 19% to 32%) after adding a ^•^OH radical scavenger (i.e., Na_2_SO_3_) to the reaction mixture. Surprisingly, it was found that adding Cu^2+^ to the reaction together with a Fe/ZMS-5 catalyst, either as a component of the heterogeneous catalyst (e.g., Cu-Fe/ZSM-5) or as a heterogeneous (e.g., Cu/silicate-1) or homogeneous additive (e.g., Cu(NO_3_)_2_) will significantly improve MeOH selectivity (>80%) while the catalyst activities remain largely unchanged. While having Cu^2+^ alone in the zeolite cannot activate methane, ^•^OH radicals were no longer observed via the EPR spectroscopy. This has led to the hypothesis that different forms of Cu in the reaction mixture eliminated the ^•^OH radicals. In an optimized setup, an impressive methane conversion of 10% with a methane selectivity of 93% was achieved, using a physical mixture of Cu/Silicalite-1 and Fe/Silicalite-1 catalyst with 1 M of H_2_O_2_, 3 bar CH_4_, 70 °C, and twice the amount of catalyst compared to the standard condition. Notably, the MeOH is not stable and will be further oxidized in the presence of H_2_O_2_ and the zeolite catalyst under these reaction conditions. The presence of an excess amount of CH_4_, therefore, had a stabilizing effect on the MeOH, possibly through CH_4_ competing for the active sites.

The observation that Cu practically eliminated the production of ^•^OH radicals is unusual because Cu and H_2_O_2_ are known to produce ^•^OH radicals. It is possible that Cu acted as a catalytic ^•^OH radical scavenger, quenching or converting ^•^OH radicals into nonparticipative species such as O_2_, H_2_O, H_2_O_2_, or even O_2_^–^.^251^ Alternatively, Cu^2+^ may deter the Fe active sites from producing ^•^OH radicals. The latter hypothesis is supported by the observation that adding Au^3+^ can have a similar (albeit smaller) boosting effect on the MeOH selectivity. However, both hypotheses have difficulties explaining the observation that a physical mixture of Fe/Silicalite-1 and Cu/Silicalite-1 can produce methane in a highly selective manner, but in this case, a high Cu:Fe ratio >10 was used, compared to about 1 for the cases when Cu was deposited onto the zeolite with Fe or by adding Cu^2+^ into the solution.

**Figure 21:**
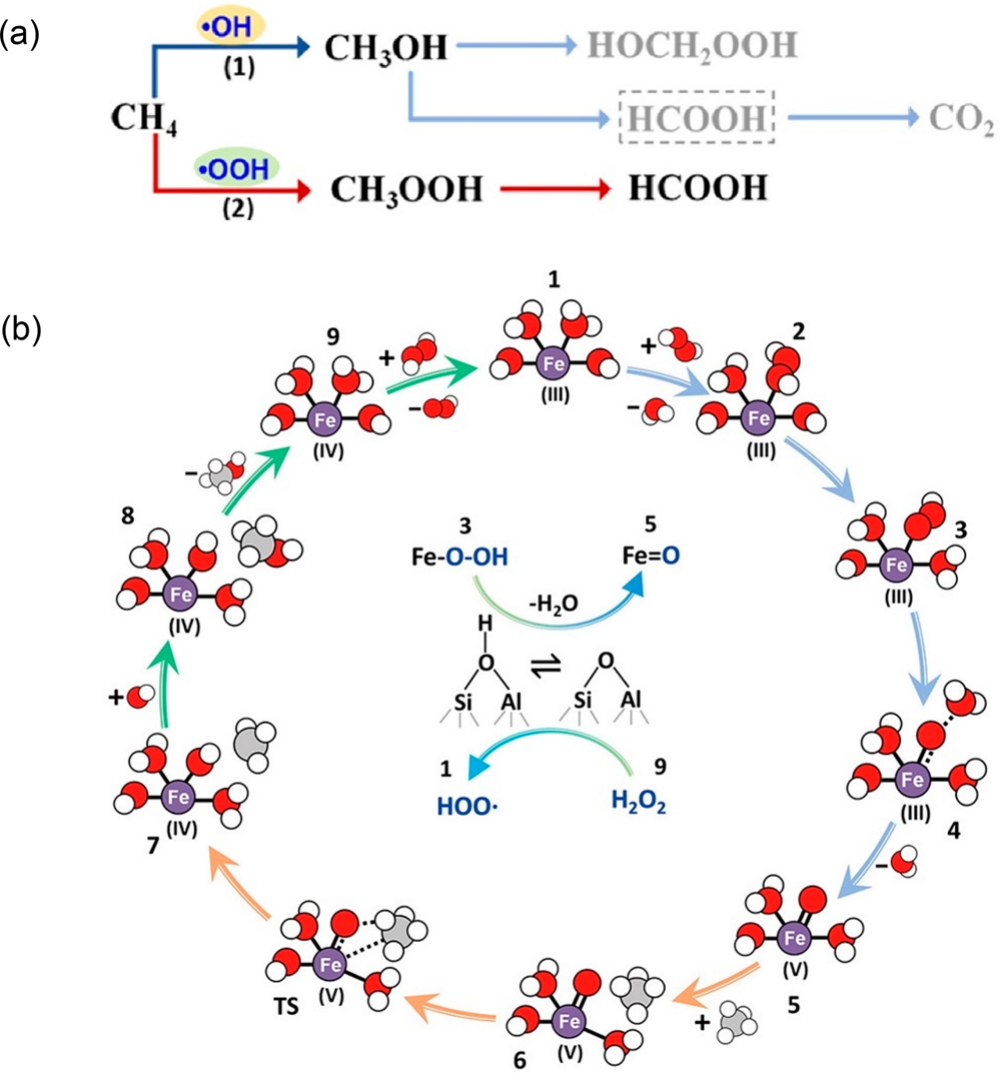
(a) Proposed two parallel pathways for direct methane oxidation to methanol in the aqueous media using H_2_O_2_ as the oxidant. (b) A posed molecular mechanism for the direct oxidation of methane to methanol over a mononuclear Fe^5+^=O active site. Red, purple, gray, and white spheres represent O, Fe, C, and H atoms, respectively. Adapted with permission from ref (308). Copyright 2021 American Chemical Society.

Even though many groups have since reproduced the excellent catalytic performance of the Cu/Fe/ZSM-5 system, the nature of active sites and the role of Cu have stirred up a lot of debate in the research community. Notably, one alternative mechanism was proposed in a recent paper by Beale, Weckhuysen, and Luo.^307,308^ Consistent with previous studies, they have also observed that 0.1 wt % Fe/ZSM-5 catalysts are highly active in the selective oxidation of methane in aqueous media containing H_2_O_2_. They also observed a significant increase in MeOH selectivity to about 80% after adding Cu to the catalysts via coimpregnation, although a Cu:Fe molar ratio of about 17 was used instead of the 1:1 ratio reported by Hammond et al.^144^ Using Mössbauer spectroscopy, the authors were able to quantify different types of Fe species present and found that only the population of the mononuclear Fe species exhibited a positive correlation with the apparent methanol turnover rates for catalysts with different Fe loadings.^308^ Hence isolated Fe mononuclear species were proposed as the active sites. Comparing the two catalysts, namely 0.1 wt % Fe/ZSM-5 and the 2 wt % Cu-0.1 wt % Fe/ZSM-5 in a time-online analysis, they were found to have different primary intermediates and products during the methane oxidation reaction. This led to the proposal that methane oxidation has two parallel pathways via ^•^OH and ^•^OOH radicals, respectively, as shown in Figure 21a. EPR studies found more ^•^OH radicals, rather than less, after adding 2 wt % of Cu onto the catalyst. Combining all the evidence, the authors also constructed a molecular level mechanism shown in Figure 21b based on the mononuclear Fe^5+^=O site, whereby CH_4_ gets activated and creates a CH_3_ radical, which reacts directly with ^•^OH to form MeOH. The active sites were then restored via interaction with H_2_O_2_. The authors proposed that the effect of having Cu is to create more OH radicals that not only facilitated the formation of MeOH but also eliminated HCOOH via quick overoxidation. The 2 wt % Cu-0.1 wt % Fe/ZSM-5 catalyst achieved a performance of 431 mol_MeOH_ mol_Fe_^–1^ h^–1^, which equated to about 7.7 mol_MeOH_ kg_Catalyst_^–1^ h^–1^.

Neither of the mechanisms described in Figures 20 and 21 can explain all of the experimental data and both have some weaknesses. For instance, in the initial studies by Hammond et al.,^144,251,303,304^ the absence of OH radicals in the earlier work was only obtained from the experiment by adding a small amount of Cu(NO_3_)_2_ to the reaction mixture, and the cases where Cu was added as a part of the catalyst or a heterogeneous additive were not examined. In some cases, a similar amount of Cu was added to the catalyst compared to the later study of Yu et al.,^308^ in which more hydroxyl radicals were indeed found. Although the diiron species matches the best match for the XAFS data, other types of Fe species most likely coexist and their contribution to the catalysis may also be significant. On the other hand, it remains challenging to quantify different types of Fe species on the catalyst. For instance, Yu et al.^308^ acknowledged that Mössbauer spectroscopy cannot distinguish diiron species from other types of oligomeric Fe species. They also observed a partial agglomeration of isolated Fe species after introducing CH_4_ during the in situ XAS and UV–vis experiments. Hence, the new evidence at least did not exclude the possibility of diiron species being the active species. In terms of the role of Cu in eliminating HCOOH and improving CH_3_OH selectivity, it is more likely that the Cu switches off HCOOH production in the first place rather than overoxidizing HCOOH as proposed by Yu et al.^308^ because no significant CO_2_ production was observed. It is possible that different types of active species and reaction mechanisms coexist and that both the materials and reaction parameters employed collectively decide the observed outcome.

Despite the debate over the mechanisms, the reaction itself has been reproduced by many and extended to other light alkanes such as ethane^309,310^ and propane.^311^ Many have also attempted to bring this chemistry from batch to flow reactors. For instance, Xu et al. reported a 0.5% conversion with 92% selectivity to methanol using a Fe-Cu/ZSM-5 catalyst, with a productivity of 0.34 mol MeOH per kg catalyst. Armstrong et al.^15^ reported a 23% ethane conversion with a selectivity to oxygenates of 98% using a Fe/ZSM-5 catalyst in a flow reactor operating at 30 bar. A shift in selectivity toward ethene from acetic acid has been observed when adding Cu into the catalyst. Klemm and colleagues^312,313^ studied methane oxidation using microchannel reactors. Due to a very low liquid-to-solid ratio, formic acid was found to be the main product even when a Cu-Fe-Silicalite-1 catalyst was used. Under optimal conditions, a selectivity to formic acid of 96.7% at a methane conversion of 10.3% could be achieved.

Many catalysts besides zeolites containing extra-framework Fe species have also been explored for the selective oxidation of methane using H_2_O_2_. Kwon et al.^314^ reported a 0.3 wt % Rh/ZrO_2_ catalyst that can achieve 0.3 mol_MeOH_ kg_cat_^–1^ h^–1^, using H_2_O_2_ at 70 °C, and no HCOOH product was reported. Interestingly this catalyst was also active at 300 °C using O_2_ as the oxidant. Cui et al.^315^ reported a graphene-confined single Fe atom catalyst that achieved 0.47 mol mol_Fe_^–1^ h^–1^ of oxygenated product at room temperature. Catalysts prepared with other 3d transition metals including Co, Ni, Cu, and Mn were all found inactive. Zhou et al.^316^ reported a nickel single-atom catalyst on N-doped amorphous carbon that can activate methane. A CH_4_ conversion of >1 mol kg_cat_^–1^ h^–1^ and a methanol selectivity of >90% was observed under optimized conditions. Many other single-atom catalysts have also been reported for activating CH_4_, including isolated Fe sites prepared on MOF^317,318^ and Cr catalysts on TiO_2_,^319^ but they were found to be much less effective in producing MeOH. These novel catalysts, however, still exhibit lower activity values when compared to the best reported Cu-Fe/ZSM-5 catalyst.^308^

#### 4.3.2 CO Assisted Methane to Methanol and Acetic Acid

Despite the progress of methane oxidation with H_2_O_2_, which is too expensive an oxidant, new processes that can utilize molecular oxygen still need to be developed. In 2017, Shan et al.^320^ reported that single atom Rh catalysts supported on zeolites can effectively convert methane to acetic acid and methanol using O_2_ and CO in aqueous media under mild conditions. A typical reaction involves 20 mg of catalyst, 0.5–4 bar of O_2_, 5 bar of CO, 20 bar of CH_4_, and 20 mL of water and was carried out at 150 °C. The optimized 0.5Rh-ZSM-5 catalyst can yield around 22000 μmol_acetic acid_ g_catalyst_^–1^ with about 60% selectivity after a 3 h reaction. Remarkably, the reaction can be tuned toward methanol by utilizing a low or nonacidic support, such as Na-ZSM-5 or TiO_2_. In the optimum case, 230 μmol_methanol_ g_catalyst_^–1^ can be achieved in 3 h with near 100% selectivity. Almost at the same time, Tang et al.^321^ independently reported similar results. In their work, single-atom Rh catalysts on zeolites were also used. and a higher acetic acid selectivity of about 70% was achieved. In both cases, acetic acid was found to be the more pronounced product. Because acetic acid is produced industrially via methanol carbonylation with CO^322^ via Rh catalysts (the Monsanto process), which was later replaced by Ir catalysts (the Cativa process), it may not be a coincidence that Rh was found to be the best catalyst. Shortly after, Ir-based catalysts were reported to be effective for similar processes. Jin et al.^323^ reported that Ir clusters and single atoms on nanodiamond convert ethane to oxygenates with an ethane conversion activity of 7.5 mol mol_metal_^–1^ h^–1^ at 100 °C. This new CO-assisted methane oxidation process is at least a few orders of magnitude more effective compared to the similar approaches reported in the 1990s by Sen and Fujiwara, as well as the more recent gas-phase reaction reported by Roman-Leshkov. It has provided another promising route for utilizing CH_4_ resources, although the activities of the catalysts remain too low to be commercialized.^320^

Many common features can be found in the studies^320,321,323−328^ of this new CO-assisted methane oxidation process, which gives us useful insights into its working mechanism. First, both CO and O_2_ are necessary for the reaction to proceed.^320,321,328^ No oxygenate products were found if either CO or O_2_ were used separately under otherwise identical conditions. Experimental results confirmed that the formation of the acetic acid involves getting the methyl group from methane, followed by a CO insertion mechanism.^320^ Neither methanol carbonylation nor the coupling between formic acid and CO or CH_4_ is a pathway for forming acetic acid. Although methanol was formed via a separate pathway that does not directly involve CO as a reactant, CO is still found necessary for the methanol formation to take place.

Second, the most active catalysts in literature often contain Rh or Ir in the form of positively charged isolated cations or small clusters. These highly dispersed species not only ensured high material-utilization efficiency but more importantly also created unique active sites for alkane activation.^320^ Nanoparticles of these precious metal tend to form CO_2_ instead.^321,329^ In terms of the catalyst support, zeolites were often used due to the presence of Brønsted acidic sites, which play at least two roles: (i) they stabilized the isolated metal cation species such as Rh^+^,^321^ (ii) they are known to promote the carbonylation reaction.^330^ Indeed, experimentally, it was found that having Brønsted acidic sites is critical for the formation of C2 oxygenates but not for methanol.^320,321^ This has allowed tuning of the reaction selectivity toward methanol with nonacidic support, as mentioned above.^320^ Moteki et al.^328^ showed that using small-pore zeolite, SSZ-13, can also suppress the acetic acid formation and promoted methanol selectivity.

It is also agreed that CO has played multiple roles in this reaction. Besides being the reactant for the carbonylation, CO serves as a ligand and can act as a reductant to maintain the desired oxidation state of the metal active centers.^323^ Other precious metals were not as efficient at producing C2 oxygenates, possibly due to their higher activities toward CO oxidation to CO_2_, thereby consuming CO.^324^ However, increasing the CO concentration could further saturate the coordination of the metal and significantly enhance the energy barrier for methane activation.^321,329^ The role of water was also investigated, although other solvents can be used such as *n*-dodecane,^321^ water is much more effective. In addition to the hydrolysis of reaction intermediates toward methanol or acetic acids, Bunting et al.^329^ suggested that water can prevent poisoning of the active sites by CO.

**Figure 22:**
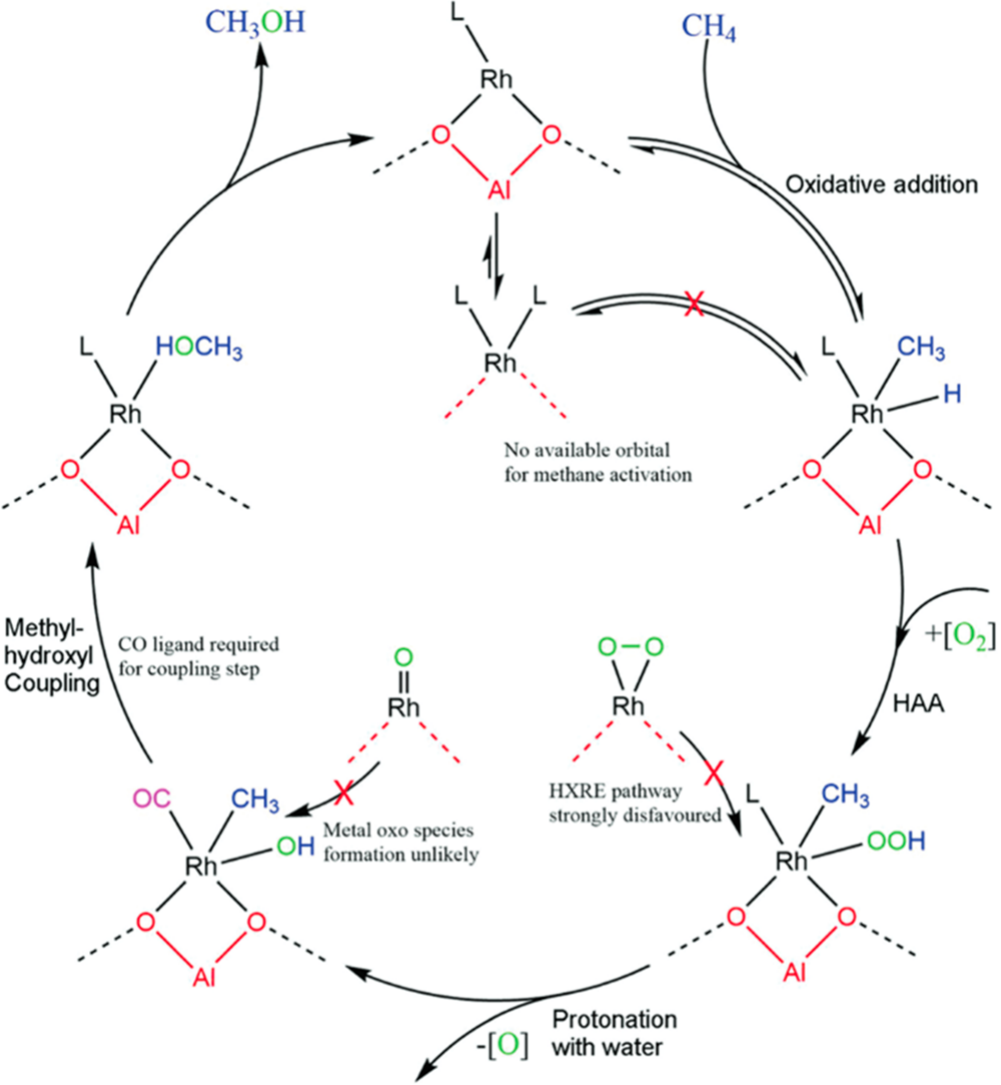
Proposed catalytic cycle for methane partial oxidation to methanol over Rh@ZSM-5. The steps, going clockwise, are methane activation, oxygen insertion, peroxide decomposition, methyl–hydroxyl coupling, and methanol desorption. CO is required for a ligand effect for the methanol formation step. Adapted with permission from ref (329). Copyright 2020 the Royal Society of Chemistry.

According to experimental observations and DFT investigations, several reaction mechanisms were proposed. Shan et al.^320^ suggested that CH_4_ is activated on the isolated Rh^+^ site and forms Rh-CH_3_, which is then functionalized through two different pathways; O-insertion toward methanol and CO insertion toward acetic acid before the hydrolysis recovers the Rh^+^ sites. A more detailed two-step mechanism was proposed by Tang et al.^321^ In the first step, Rh cations in the zeolite first interacted with the O_2_ to form the Rh_1_O_5_ species, which then activates CH_4_ with an energy barrier of 1.29 eV to form methyl and hydroxyl groups on the Rh atom. Then CO is inserted into the Rh–O bond and forms COOH, which is coupled with the methyl group to give out acetic acid. The second step begins with the remaining Rh=O oxo group that can activate a second CH_4_ molecule to again form methyl and hydroxyl groups, while CO binds with the unsaturated Rh site and then is inserted into the Rh-CH_3_, followed by coupling with the hydroxyl group to form a second acetic acid molecule with a relatively low energy barrier of 0.72 eV. Meanwhile, the DFT investigation by Bunting et al.^329^ focused on the methanol formation pathway (Figure 22). Similarly, they showed that methane will be activated at the unsaturated metal center, followed by the formation of a peroxide species via the oxygen insertion, which is energetically more favorable than the alternative routes such as the hydrogen reductive elimination or the formation of Rh=O oxo species. Then the methanol was formed by a methyl-hydroxyl coupling step which is the rate-determining step. It was found that the presence of the CO-ligand, as well as the presence of water, are necessary to reduce the energy barrier for this step. It is interesting to see that “partially CO-poisoned” metal sites are necessary for the methanol formation to take place on this route.

The involvement of peroxide species in the mechanism described in Figure 22 somewhat resembles the H_2_O_2_ route discussed in section 4.3.1. Notably, Cu and Pd were found to be effective additives to promote methanol production. For instance, Li et al.^325^ reported that IrCu and IrCuPd catalysts can produce methanol much more effectively compared to Ir catalysts and their original Rh catalysts. The optimum methanol productivity reaches 1200 μmol_methanol_ g_catalyst_^–1^ and can be achieved in 1 h at 150 °C with a selectivity of ∼80%. It was believed that the Cu hindered the formation of hydroxyl radicals from peroxide species, hence decreasing the tendency for methanol overoxidation, just like in the case of the Fe catalysts in the H_2_O_2_ route. Pd was thought to promote methane activation with the presence of *in situ* formed H_2_O_2_. It may be inferred that these bimetallic or trimetallic systems enabled a more complicated reaction network that differs from the original studies discussed earlier and are more closely aligned to the H_2_O_2_-based mechanisms discussed in section 4.2.

#### 4.3.3 Copper Modified Zeolite for Methane Oxidation to Methanol

Copper modified zeolites (Cu-zeolites) were initially used in a selective catalytic reduction (SCR) process for cutting NO_*x*_ emissions from compression-ignited diesel engines.^331^ Cu-zeolite catalysts were also employed in the decomposition of nitrogen-containing exhaust gases to N_2_ and O_2_.^332^ In recent years, the catalytic applications of Cu-zeolites toward the production of value-added products has rapidly increased. Many reports show that Cu-zeolites have marked activity in the selective oxidation of hydrocarbons,^333^ particularly in the most challenging catalytic reaction, namely the partial oxidation of the methane C–H bond to produce methanol using O_2_ as an oxidant.^146,226,334^ The aim of this section is to provide a review on the conversion of methane into methanol over Cu-zeolites. The methanol productivity on Cu-zeolites depends on the catalyst preparation strategies and catalytic routes, which are closely related to the properties of the zeolite support,^335,336^ such as the morphological/topological structure and the Si/Al ratio. These factors will be discussed in the subsection ***Catalyst and reaction process***. Clarifying the active Cu sites is the critical step to developing Cu-zeolite catalysts that are highly active toward methane conversion. The formation, identification, structure and properties of active Cu sites and their catalytic activity will be discussed in the subsection ***Active Cu Sites***. The last part of this section, ***Reaction mechanism***, deals with the experimental and theoretical understanding of the catalytic mechanism for the conversion of methane into methanol on Cu zeolites, with a particular focus on how methane is activated (i.e., by direct dissociation or stepwise reduction–oxidation) on active Cu sites and what is the effect of the water on the methane to methanol reaction.

##### 4.3.3.1 Catalyst Structure and Reaction Mechanism

###### Two-Step Stoichiometric Reaction

**Figure 23:**
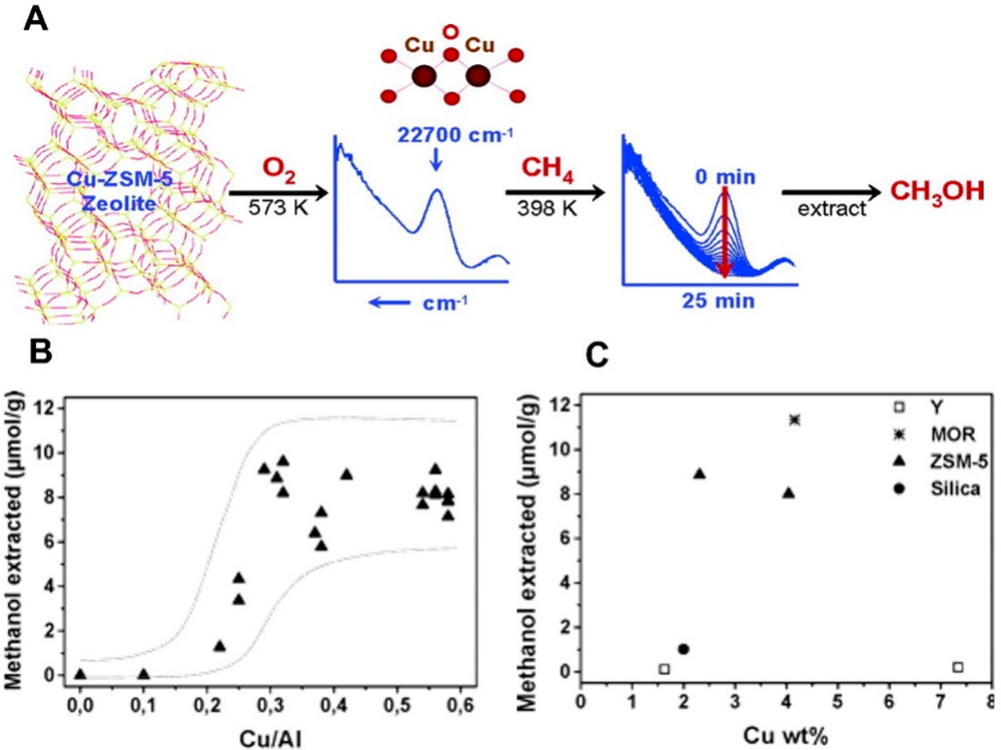
(A) Two-step stoichiometric methane-to-methanol reaction on Cu-ZSM-5 zeolite, in which O_2_ was first activated to form [CuO_2_Cu]^2+^ species on Cu-ZSM-5, then CH_4_ was introduced with CH_3_OH detected after extraction. (B) Amount of methanol extracted per gram of Cu-ZSM-5 sample as a function of the Cu/Al ratio of the Cu-ZSM-5 samples. (C) Amount of methanol extracted per gram of Cu sample as a function of the Cu wt % of the Cu containing zeolites. Adapted with permission from ref (335). Copyright 2005 American Chemical Society.

The work of Groothaert et al.^335^ showed that Cu modified ZSM-5 and MOR zeolites, after being activated in O_2_ or N_2_O, could oxidize methane to methanol with high selectivity. This was achieved in a stoichiometric way, which was often operated by a two-step process as shown in Figure 23A. Cu-zeolite was activated by O_2_ at a temperature above 300 °C to a highly activated bis(μ-oxo)dicopper species in the first step, and then methane was introduced onto the activated catalyst at temperatures of at least 125 °C. Extraction of the catalyst using acetonitrile and water gave a highly selective production of methanol (98%). GC analysis showed no products in the reactor outlet, and elevating the desorption temperature to 300 °C caused overoxidation to CO_2_. This indicated that the methanol desorption from the catalyst might be a problem in an online process.

Table 2 summarizes the Cu-zeolites that have been used for the catalytic conversion of methane into methanol. Previous studies showed that the preparation method for the Cu-zeolites influences their methane-to-methanol performance. It was found that methanol productivity increased linearly with the Cu/Al ratio in the range of 0.1–0.32 and stayed around the maximum value of ca. 9 μmol g^–1^ in the Cu/Al ratio range of 0.32–0.58 after methane reaction (175 °C) on O_2_-activated (450 °C) Cu-ZSM-5 zeolites (Figure 23B, and Table 2, entries 1–7). This indicates that not all the Cu species acted as sites for the activation of methane. Assuming a Cu/methanol stoichiometric ratio of 2:1, it can be estimated that less than 5% of the Cu atoms in the Cu-ZSM-5 zeolite with a Cu/Al ratio of 0.32 are actually involved in production of methanol.^335^ In comparison (Figure 23C and Table 2), Cu-ordenite (MOR) (Si/Al = 8.8, Cu/Al = 0.43) produced a higher methanol yield (11.34 μmol g^–1^) than Cu-ZSM-5 (Table 2, entry 20), while zeolite Y (Si/Al = 2.7, Cu/Al = 0.05 and 0.29, Table 2, entries 30 and 31) and amorphous silica (Si/Al = 141, 2 wt % Cu) showed very low methanol yields (below 1 μmol g^–1^). The Si/Al ratio of Cu-zeolites also influences the catalytic activity. The methanol productivity gradually decreased with increasing Si/Al ratios on Cu-ZSM-5 (Table 2 entries 8–12).^336^ A linear correlation between the methanol yields was found for the samples with a Si/Al ratio range of 12 to 120. In contrast to Cu-ZSM-5, the methanol yield was found to increase with the Si/Al ratio (5.3 and 8.8) on Cu-MOR zeolites.

**Table 2:** Conditions and Performances of the Two-Step Stoichiometric Methane-to-Methanol Reaction on Various Cu Modified Zeolites

					O_2_	CH_4_	CH_4_	methanol	methanol		
entry	zeolite	Si/Al ratio	Cu/Al ratio	preparation	activation *T* (°C)	reaction *T* (°C)	pressure (bar)	productivity (μmol g^–1^)	selectivity (%)	extraction method	ref
1	ZSM-5	12	0.1	IE	450	175	1	0		a	(335)
2	ZSM-5	12	0.22	IE	450	175	1	1.27		a	(335)
3	ZSM-5	12	0.25	IE	450	175	1	3.8		a	(335)
4	ZSM-5	12	0.32	IE	450	175	1	8.9		a	(335)
5	ZSM-5	12	0.42	IE	450	175	1	9		a	(335)
6	ZSM-5	12	0.54	IE	450	175	1	8.2		a	(335)
7	ZSM-5	12	0.58	IE	450	175	1	8.2	98	a	(335)
8	ZSM-5	12	0.54	IE	450	150	1	8.19		a	(336)
9	ZSM-5	25	0.51	IE	450	150	1	4.1		a	(336)
10	ZSM-5	30	0.47	IE	450	150	1	2.7		a	(336)
11	ZSM-5	77.5	0.55	IE	450	150	1	0.9		a	(336)
12	ZSM-5	120	0.88	IE	450	150	1	0.66		a	(336)
13	ZSM-5	11.5	0.34	IE	450	200	1	16		b	(337)
14	ZSM-5	11.6	0.53	IE	400	200	8	31	52	a	(338)
15	ZSM-5	11.6	0.53	IE	400	360	8	0		a	(338)
16	Na-ZSM-5	14	0.65	IE	450	200	1	9		a	(339)
17	MOR	5.3	0.39	IE	450	150	1	3.6		a	(336)
18	MOR	5.3	0.39	IE	450	200	1	13		a	(336)
19	MOR	8.8	0.5	IE	450	150	1	6		a	(336)
20	MOR	8.8	0.43	IE	450	175	1	11.34		a	(335)
21	MOR	8.8	0.5	IE	450	200	1	16		a	(336)
22	MOR	5	0.34	IE	450	200	1	31		b	(337)
23	MOR	10.5	0.39	IE	400	200	8	119	91	a	(338)
24	MOR	10.5	0.39	IE	400	360	8	5		a	(338)
25	MOR	10	0.27	SSIE	450	200	1	37.3		a	(340)
26	MOR	10	0.27	SSIE	550	200	1	55.3		a	(340)
27	MOR	10	0.27	SSIE	650	200	1	65.2		a	(340)
28	Na-MOR	5	0.3	IE	450	200	1	21		a	(339)
29	Na-MOR	8.5	0.38	IE	450	200	1	25.8		a	(339)
30	Y	2.7	0.05	IE	450	175	1	<1		a	(335)
31	Y	2.7	0.29	IE	450	175	1	<1		a	(335)
32	Y	2.7	0.45	IE	450	200	1	<1		a	(336)
33	Na-Y	3	0.32	IE	450	200	1	<1		a	(339)
34	Y	2.6	0.41	IE	400	360	1	90	91	a	(338)
35	Y	2.6	0.41	IE	400	360	1	88	92	b	(338)
36	Y	2.6	0.41	IE	400	360	8	303	93	b	(338)
37	Y	2.6	0.41	IE	400	360	15	360	93	b	(338)
38	USY	27.5	0.32	IE	450	200	1	<1		a	(336)
39	EMT	4	0.36	IE	450	150	1	<1		a	(336)
40	FER	6.2	0.42	IE	450	150	1	1.6		a	(336)
41	FER	6.2	0.42	IE	450	200	1	12		a	(336)
42	Na-FER	8.9	0.38	IE	450	200	1	10.4		a	(339)
43	BEA	9.8	0.5	IE	450	150	1	1.6		a	(336)
44	BEA	9.8	0.5	IE	450	200	1	4.2		a	(336)
45	BEA	12.4	0.4	IE	400	200	8	55	98	a	(338)
46	BEA	12.4	0.4	IE	400	360	8	8		a	(338)
47	SSZ-13	6	0.35	IE	450	200	1	28		b	(337)
48	SSZ-13	12	0.35	IE	450	200	1	31		b	(337)
49	Na-SSZ-13	15.8	0.84	IE	450	200	1	30		a	(339)
50	SSZ-16	6.5	0.34	IE	450	200	1	39		b	(337)
51	SSZ-36	10	0.26	IE	450	200	1	36		b	(337)
52	SAPO-34	6	0.6	IE	450	200	1	15		b	(337)
53	OME	3.2	0.07	IE	450	200	1	1.9		a	(339)
54	Na-OME	3.2	0.12	IE	450	200	1	17.7		a	(339)
55	Na-OME	3.2	0.29	IE	450	200	1	86.1		a	(339)
56	Na-OME-fast	4.3	4.78 wt %	IE	450	200	1	*ca*. 100		a	(341)
57	Na-OME-slow	4.3	4.64 wt %	IE	450	200	1	150.9		a	(341)
58	ECR	3.5	0.09	IE	450	200	1	2.6		a	(340)
59	Na-ECR	3.5	0.14	IE	450	200	1	9		a	(340)
60	Na-ECR	3.5	0.33	IE	450	200	1	19.7		a	(340)

a Extraction of methanol with water at ambient temperature.

b Desorption of methanol in a stream of wet inert gases at an elevated temperatures ≥200 °C.

Reaction conditions have also been intensively investigated for the conversion of methane to methanol. Reducing the methane reaction temperature from 175 to 150 °C did not affect the methanol productivity on Cu-ZSM-5 (Table 2 entry 6 and 8), but it did cause a significant reduction of methanol yields on Cu–MOR (Table 2 entry 19 and 20). Elevating the reaction temperature to 200 °C resulted in higher methane yields on both Cu-ZSM-5^337^ (Table 2 entry 13) and Cu–MOR zeolites^336^ (Table 2 entry 18 and 21). For Y and USY zeolites, the methanol yields were lower than 1 μmol g^–1^ even at a high reaction temperature (Table 2 entry 32 and 38).^336^ Besides reaction temperature, it was found that the methane reaction pressure significantly affected the methanol yield on Cu-zeolites. For Cu loaded ZSM-5, MOR, and BEA zeolites, increasing the methane pressure to 8 bar, notably enhanced catalytic performance. Cu–MOR showed the highest methanol yield of 119 μmol g^–1^ and Cu–BEA produced the highest methanol selectivity of 98%, while methanol productivity (16 μmol g^–1^) and selectivity (52%) were observed on Cu-ZSM-5 catalyst (Table 2 entries 14, 23, and 45).^338^ Further increasing the reaction temperature caused a sharp drop in methanol yields on these three zeolites (Table 2 entries 15, 24, and 46). Cu–Y zeolites had been previously considered to be inactive for methane conversion to methanol under conventional conditions (catalyst activated in O_2_ at 450 °C and methane reaction at 200 °C)^335,336^ (Table 2, entries 30–33). However, in the work by van Bokhoven et al., the methane reaction was operated at 360 °C on a Cu–Y zeolite (catalyst activated in O_2_ at 400 °C), which produced a high methanol yield of 90 μmol g^–1^ and 92% selectivity even at ambient methane reaction pressure (Table 2, entry 34 and 35). Further increasing the methane reaction pressure to 8 and 15 bar produced an unprecedented high methanol yield of 303 and 360 μmol g^–1^, with more than 90% selectivity, respectively (Table 2, entry 36 and 37). Thermal O_2_ activation in the range of 400–750 °C is often required for the generation of catalyst activity. The Cu-zeolites prepared by conventional liquid-phase ion exchange showed a limited methanol productivity even under intense O_2_ activation conditions,^342,343^ i.e., higher than 550 °C. However, a report by Le and co-workers indicated that the methanol productivity was remarkably and continuously enhanced by raising the O_2_ activation temperature up to 650 °C on a Cu-MOR catalyst prepared by a solid-state ion-exchange (SSIE) approach.^340^ As listed in Table 2, entries 25–27, the reaction with methane at 200 °C yielded 37.3, 55.3, and 65.2 μmol g^–1^ of methanol using Cu–MOR zeolites activated at 450, 550, and 650 °C, respectively. No significant decrease in methanol productivity was observed even at O_2_ activation temperatures up to 750 °C. It is worth noting that Cu–MOR catalysts prepared by solid state ion exchange, SSIE, exhibited a higher activity for the conversion of methane to methanol than Cu–MOR prepared by conventional liquid-phase ion exchange, when activated by O_2_ at 450 °C (Table 2, entry 18 and 21).

Besides ZSM-5, MOR, and Y zeolites, various zeolites with different pore sizes, properties, and morphology have been used as supports for Cu-zeolites in the conversion of methane into methanol. By comparison, Cu-modified EMT, FER, BEA, and Omega zeolites showed extremely low methanol productivity under the conditions of O_2_ activation at 450 °C and methane reaction at 150 or 200 °C (Table 2, entries 39–41, 43, and 53).^336,339^ Wulfers and co-workers reported for the first time that a variety of copper-containing small-pore zeolites (i.e., with 8-membered rings) such as Cu-SSZ-13, Cu-SSZ-16, Cu-SSZ-39, and Cu-SAPO-34 also display some activity for methane to methanol conversion.^338^ Generally, the methanol productivity on these small-pore zeolites is higher than on the medium-pore ZSM-5 and large-pore MOR tested under the same reaction conditions (Table 2, entry 13 and 22). The small-pore zeolites were crystallographically different from ZSM-5 and MOR, having a lower framework density (in terms of tetrahedral “T” sites, 15.1 T nm^–3^ for SSZ-13 vs 18.1 T nm^–3^ for ZSM-5) and larger micropore volume (ca. 700 m^3^ g^–1^ for SSZ-13 vs ca. 400 m^3^ g^–1^ for ZSM-5). Those features would be favorable for the dispersion of active Cu site and thus higher catalytic performance for methanol production.

Methanol extraction is a critical step in the two-step methane conversion process. Work by Grothaert et al., indicated that at 300 °C, CO_2_ was produced from Cu-ZSM-5 due to the presence of residual methanol even after the methanol extraction step had been carried out. The observed level of CO_2_ was much higher following low temperature extraction than for methanol extracted at ambient temperature (Table 2 entry 7).^335^ This was explained by incomplete methanol extraction at low temperatures, leaving methanol in the Cu-ZSM-5 samples which could be oxidized to CO_2_ when the temperature was increased. Replacing off-line extraction with liquid water at ambient temperature by online desorption in a stream of wet inert gas at elevated temperature is helpful for methanol desorption. Taking advantage of the online approach, Wulfers and co-workers obtained a higher amount of methanol than previous reports, particularly for ZSM-5 (Table 2, entry 4 and 13) and MOR samples (Table 2, entry 18 and 22).^337^ Recently, van Bokhoven et al. reported a comparison of the two methods for methanol desorption on Cu–Y catalysts, showing that the methanol yield obtained by 2-fold extraction with 2–4 mL of pure water is similar to that obtained by desorption using a wet stream of helium (2.6 vol % H_2_O, 40 mL min^–1^, 1 bar) (Table 2, entry 34 and 35).^338^

Many studies have explored the effect of metal cations (e.g., Na^+^) on methane oxidation performance over Cu-zeolites. Park et al. prepared a series of Cu-Zeolites using Na-form zeolites as the supports.^339^ The amount of methanol produced on Cu-Na-ZSM-5 (Table 2, entry 16) was equivalent to that on a copper exchanged H-ZSM-5 catalyst (Cu-ZSM-5). Similar cases were reported on Na–MOR zeolites with different Si/Al ratios (Table 2, entry 28 and 29), Na–Y (Table 2, entry 33), Na-FER (Table 2, entry 42), and Na-SSZ-13 (Table 2 entry 49). These results indicate that Na^+^ ions have a negligible influence on the active Cu sites in zeolites. Interestingly, Omega (OME) and ECR zeolites are exceptions (Table 2, entry 54 and 59), producing higher amounts of extracted methanol on the copper exchanged Na-form zeolite than on the catalyst prepared using the H-form (Table 2, entry 53 and 58). Much higher methanol productivity values of 86.1 and 19.7 μmol g^–1^ were obtained on the two Na-form zeolites, respectively, by further increasing the copper loading (Table 2, entry 55 and 60). The nonrandom distribution of Na^+^ ions in these zeolite channels^344,345^ could affect the distribution of the Cu^2+^ ions in the zeolite, resulting in a high methane-to-methanol activity.

**Figure 24:**
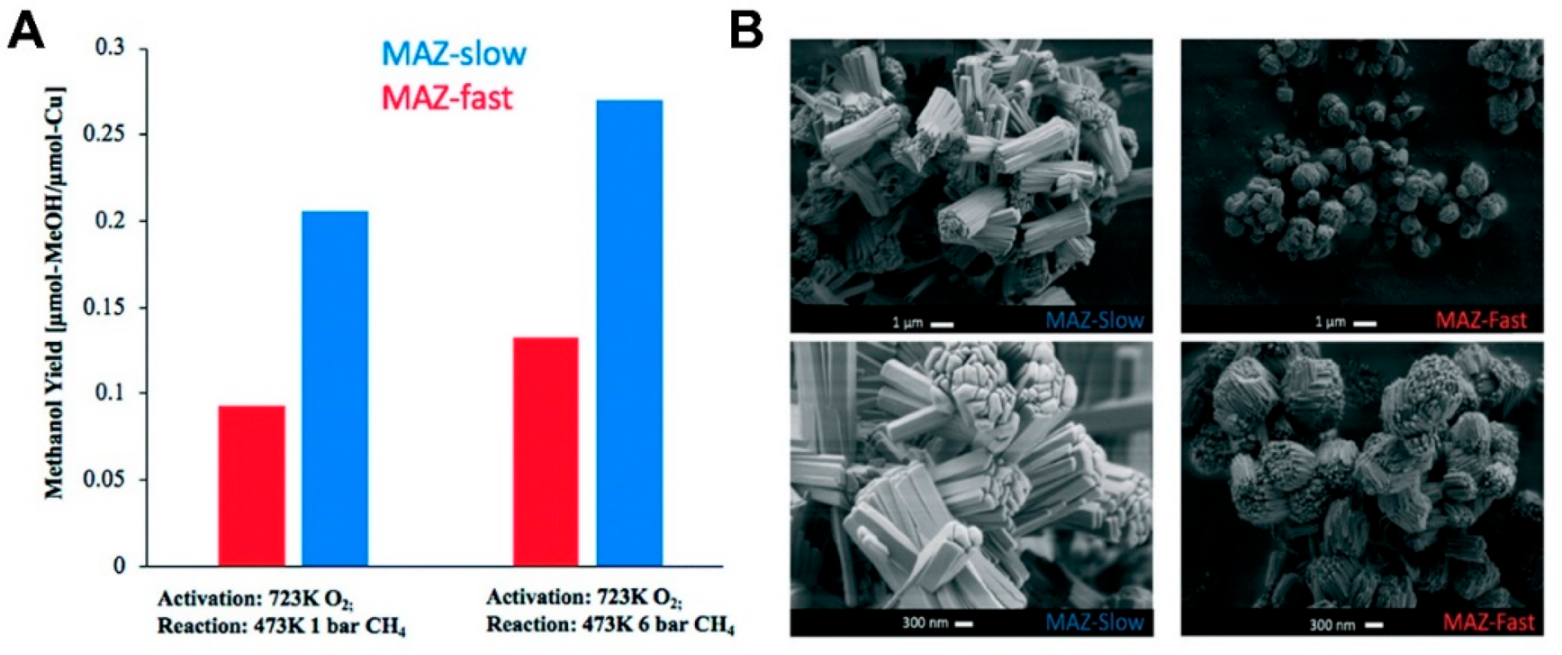
(A) Methanol yield per copper for MAZ-fast and MAZ-slow for different stepwise procedures for the conversion of methane to methanol. (B) SEM micrographs of MAZ-slow and MAZ-fast. Adapted with permission from ref (341). Copyright 2011 The Royal Society of Chemistry.

Zeolite morphology also plays an important role in the methane to methanol reaction. The conversion of methane into methanol on Cu–OMG zeolite was demonstrated to be influenced by the zeolite morphology.^341^ Two omega zeolites (MAZ topology) denoted MAZ-fast and MAZ-slow were crystallized at different rotational speeds in the hydrothermal process. As shown in Figure 24A, MAZ-slow yields 150.9 μmol g^–1^ of methanol, which is nearly 1.5–2 times more methanol than from the MAZ-fast sample. Characterization of the samples indicated that the two zeolites have the same framework structure, Si/Al ratio, and BET surface area. The only differences between the two zeolites were morphology and crystallite size. The SEM images presented in Figure 24B show the MAZ-slow material has bundles of interconnected rods with lengths of 2–4 μm and 100 nm thickness, while the MAZ-fast showed spherulitic aggregates of small rods with lengths of ∼300 and 10 nm thickness. The influence of the zeolite morphology on the methane oxidization performance was confirmed by changing the synthesis conditions (i.e., silicon source or structure directing agent) to obtain similar morphologies. The long-bundled rods produced methanol yields of around 140–150 μmol g^–1^, which was significantly reduced on the zeolites displaying spherulitic aggregates.

###### Isothermal and Cyclic Reaction

**Figure 25:**
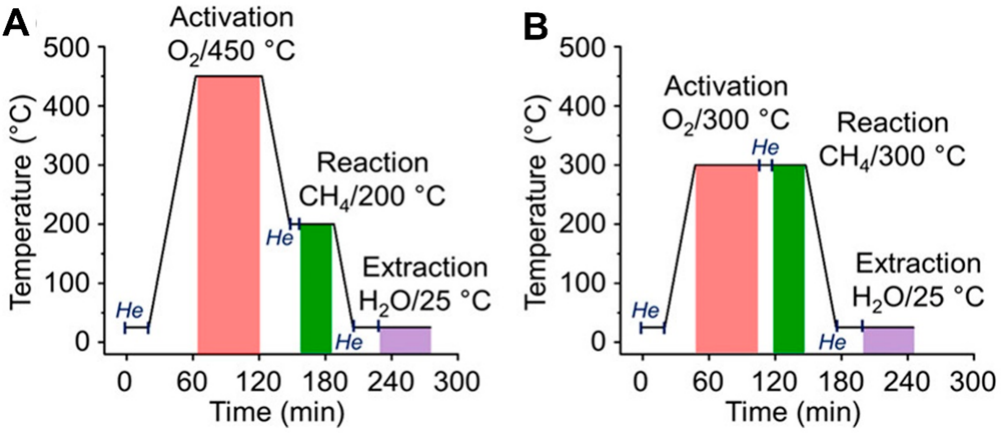
Comparison of (A) the conventional procedure and (B) the isothermal procedure of the stepwise oxidation of methane to methanol with offline water extraction. Adapted with permission from ref (346). Copyright 2020 American Chemical Society.

Cyclic operation of the two-step stoichiometric reaction is an effective way to improve methanol production. However, in the conventional two-step reaction, Cu-zeolites were activated with oxygen at temperature (e.g., 450 °C), followed by the reaction with methane at a lower temperature (e.g., 200 °C), and finally methanol was obtained by water extraction at ambient temperature or desorption by steaming at an elevated temperature (Figure 25A). The repetitious heating and cooling procedure involved in this approach limits its practical application. Indeed, van Bokhoven et al. found that low temperature O_2_ activation was sufficient for Cu-zeolites to convert methane into methanol, for example by activation of Cu–MOR at 200 °C and Cu-erionite (ERI) at 300 °C.^343^ These results led to the development of an isothermal methane to methanol process over Cu-zeolites, in which both oxygen activation and methane reaction were operated at the same temperature (Figure 25B).

**Figure 26:**
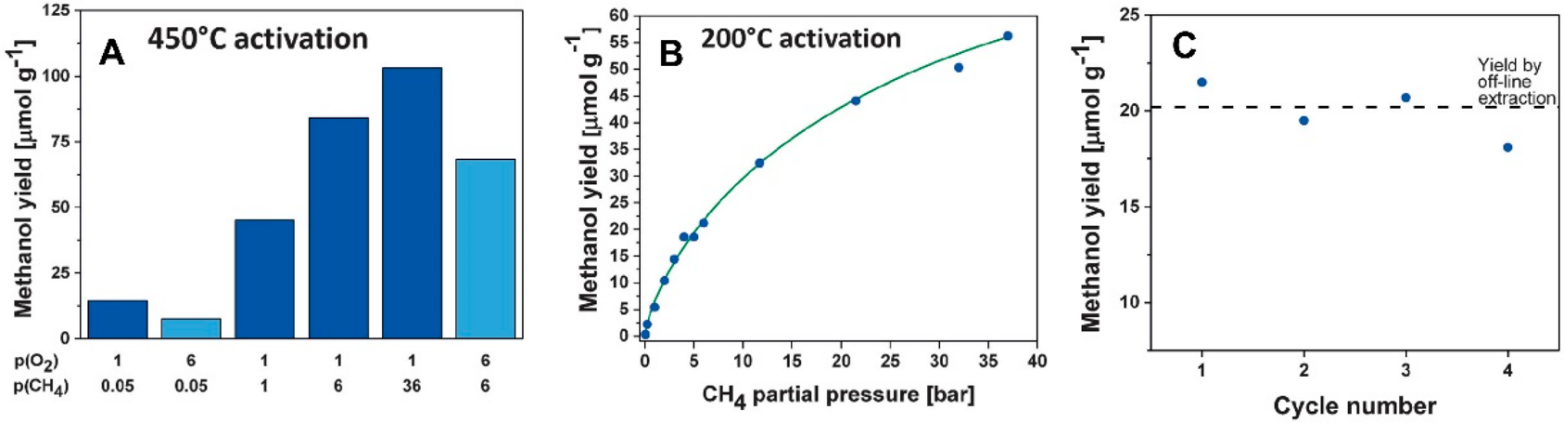
Catalytic cycle of methane oxidation to methanol on Cu–MOR. (A) Methanol yields after activation at 450 °C and off-line extraction at different pressures of oxygen and methane. (B) Dependence of methanol yield on methane pressure after 13 h activation at 200 °C, 1 bar of oxygen, and off-line extraction. (C) Methanol yields after consecutive cycles, consisting of activation for 8 h at 1 bar of oxygen, reaction with methane at 6 bar, and extraction with steam. The liquid was collected by condensation of the reactor effluent. Adapted with permission from ref (343). Copyright 2016 John Wiley and Sons.

The isothermal process, avoiding the tedious heating and cooling procedure, is more suitable for the cyclic methane-to-methanol reaction than the conventional process. The effect of oxygen and methane pressures on methanol yield was analyzed on Cu-MOR catalyst (Si/Al = 6, 4.7 wt % Cu) (Figure 26A). Activation at 732 K under 1 bar of oxygen and reaction with methane (5% in helium) at 723 K under the pressure of 6 and 36 bar yielded 84.1 to 103.3 μmol g^–1^ of methanol, respectively, which were comparable to the values reported in the two-step stoichiometric reaction (Table 2, entry 23).^338^ It was found that increasing the methane reaction pressure was also helpful for methanol productivity on Cu–MOR activated in oxygen at a lower temperature of 473 K (Figure 26B). The cyclic reactions were carried out by activation for 8 h with 1 bar of oxygen and subsequent reaction under 6 bar of methane at 473 K, followed by online extraction of the produced methanol with water at the same temperature. As shown in Figure 26C, a stable methanol yield of about 20 μmol g^–1^ was achieved in each cycle, demonstrating the feasibility of a continuous cyclic process and the stability of Cu–MOR catalyst.

**Figure 27:**
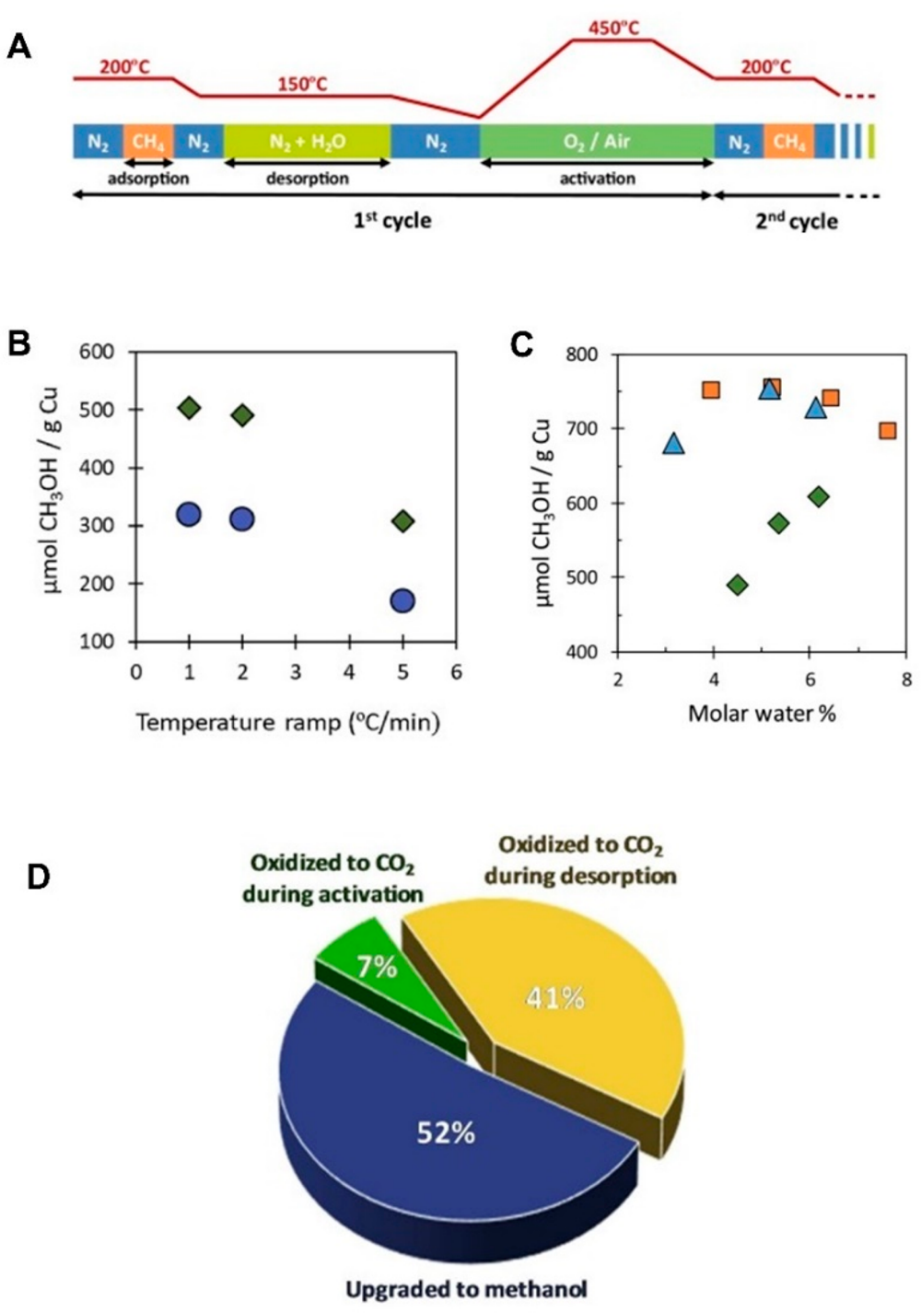
(A) Three-step cyclic reaction of methane oxidation to methanol on Cu–MOR. (B) Effect of regeneration gas composition and temperature ramp on methanol yield. Symbols: activation with pure oxygen (blue dot) and activation with synthetic air (green diamond). (C) Effect of desorption gas composition and N_2_ flow rate on methanol yield. Symbols: 150 mL min^–1^ (yellow square), 190 mL min^–1^ (blue triangle), and 220 mL min^–1^ (green diamond). (D) Percentage of the CH_4_ adsorbed on the catalyst that was transformed into methanol (blue: 52%), fully oxidized during desorption (yellow: 41%), and eliminated during the activation of the catalyst (green: 7%). Adapted with permission from ref (348). Copyright 2020 Elsevier.

Kim and co-workers optimized the preparation, pretreatment, and reaction conditions for methane conversion on Cu–MOR, obtaining only 4% enhancement of methanol productivity.^347^ To maximize the methanol yield, Álvarez and co-workers developed a three-step cyclic procedure on Cu–MOR catalys.^348^ As shown in Figure 27A, methane was fed at 200 °C in the adsorption step. Then, methanol was desorbed by feeding a flow of water in nitrogen gas at 150 °C. Finally, the catalyst was activated by oxygen or air at 450 °C. Ambient pressure (1 bar) was selected for all the adsorption, desorption, and activation steps. They found that the methanol yield was significantly influenced by the activation and desorption conditions. Figure 27B shows the impact of gas composition and temperature ramps on the methanol yield in the activation step. Using synthetic air (20% oxygen) instead of pure oxygen led to a notable increase in methanol productivity. An elevation of the activation temperature ramp from 1 to 2 °C min^–1^ has no influence on the reaction, but further increasing to 5 °C min^–1^ resulted in a remarkable reduction on methanol yield. This indicates that the catalyst can only be efficiently regenerated under lower oxygen composition and slow temperature ramp. The effect of water concentration and desorption gas flow rate on the reaction is shown in Figure 27C. The preliminary desorption flow rate was a N_2_ flow rate of 220 mL min^–1^.

Increasing the water amount from 4.5 to 6.2% at this flow rate resulted in increased methanol production, but a maximum was not reached because no more water can be introduced into the extraction gases at such a rapid flow rate (Figure 27C, green diamond). When the flow rate was reduced to 190 or even 150 mL min^–1^, the maximum methanol 754 μmol g^–1^ Cu (corresponding to 32 μmol g^–1^ Cu–MOR) was desorbed. A methanol yield of 725 μmol g^–1^ Cu was observed after 18 reaction cycles under the optimized reaction conditions. There was only a 3.9% reduction in methanol yield after 18 reaction cycles, indicating the Cu–MOR catalyst is quite stable. The maximum methanol yield of 754 μmol g^–1^ Cu is equivalent to 34 μmol g^–1^ catalyst, which was much higher than that (5 μmol g^–1^ catalyst) obtained in van Bokhoven’s isothermal procedure at ambient pressure. To further understand the three-step cyclic reaction, the amount of methane adsorbed on the Cu-MOR was analyzed by a temperature-programmed desorption in air flow without the addition of water. Because the adsorbed methane could be oxidized to CO_2_ during high temperature desorption, quantitative analysis of the CO_2_ production showed the adsorbed methane was 1482 μmol g^–1^ Cu. Taking the maximum methanol yield of 754 μmol g^–1^ Cu into account, only 52% of the adsorbed methane was converted to methanol. The amount of CO_2_ desorbed from the activation step was also analyzed, which was only 7% of the total adsorbed methane. Thus, 41% of the methane was calculated to be oxidized to CO_2_ during desorption (Figure 27D).

**Figure 28:**
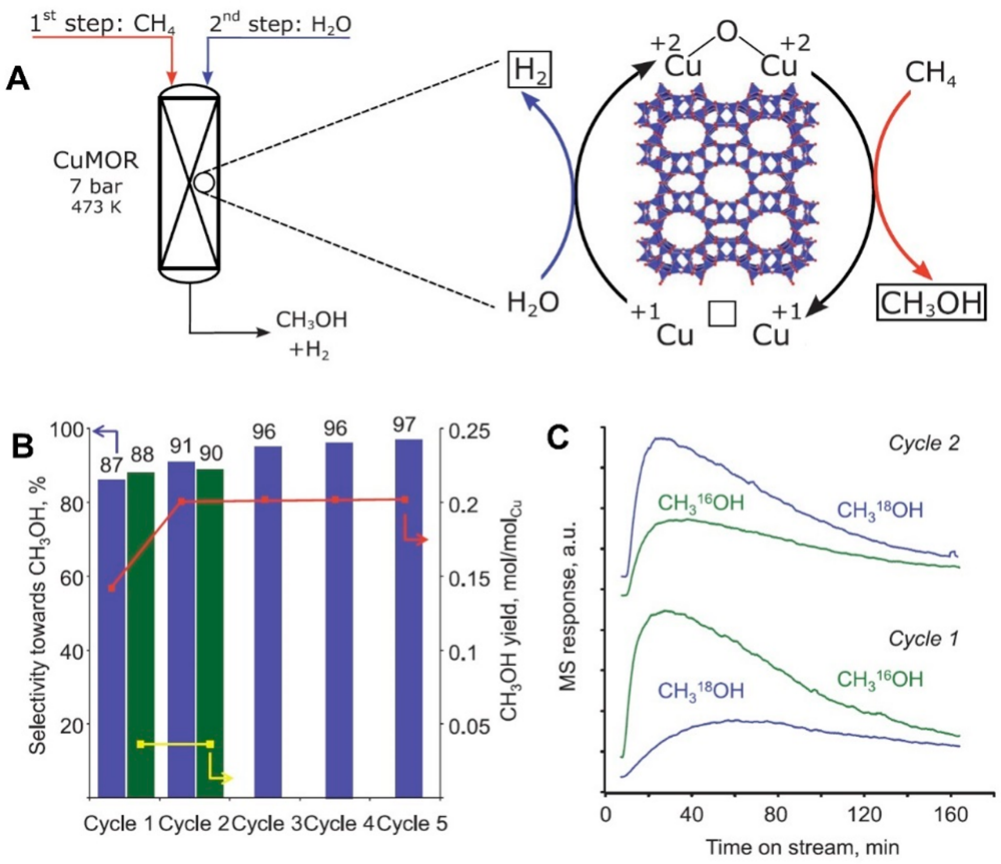
(A) Schematic representation of the reaction conditions of the partial oxidation of methane by water, involving the regeneration of the active oxygen site on Cu–MOR by water. (B) Methanol yield and selectivity across multiple cycles, each involving a helium activation at either 400 °C (red line and blue bars) or 200 °C (yellow line and green bars), followed by methane reaction and then catalyst reactivation by water at 200 °C. (C) Mass spectral responses for unlabeled (*m*/*z* = 31) and ^18^O-labeled (*m*/*z* = 33) methanol after first and second cycle with labeled H_2_^18^O, respectively. Adapted with permission from ref (349). Copyright 2017 AAAS.

To avoid the overoxidation of methane to CO_2_, water was used as the soft oxidant for methane oxidation on Cu–MOR zeolite.^349^ As illustrated in Figure 28A, the methane conversion to methanol was performed in multiple cycles. First, Cu–MOR was activated at 400 or 200 °C in a He flow, followed by cooling to 200 °C and exposure to 7 bar CH_4_. Then, water vapor in a flow of helium was introduced into the reactor to desorb the products. Thereafter, the second cycle started by treating the catalyst under He flow. The methane oxidation over a 400 °C activated Cu–MOR was shown in Figure 28B (red line and blue bars), 0.142 mol_methanol_ mol_Cu_^–1^ being detected in the first cycle with a relatively low selectivity of 87%. In the second cycle, both selectivity and methanol yield, increased substantially, suggesting that water is able to regenerate the active sites. The catalyst was stabilized after three reaction cycles, showing a methanol productivity of 0.202 mol_methanol_ mol_Cu_^–1^ and a selectivity of 97%. Reducing the activation temperature to 200 °C led to a remarkable reduction in methanol productivity. However, almost no obvious influence was observed on the selectivity (Figure 28B, yellow line and green bars). This implied insufficient regeneration of active sites at the low activation temperature. ^18^O-Labeled water resulted in the formation of ^18^O-labeled methanol (Figure 28C), confirming water as “soft” oxidant for the high selective production of methanol on Cu-MOR zeolites.

###### Direct Catalytic Reaction

**Figure 29:**
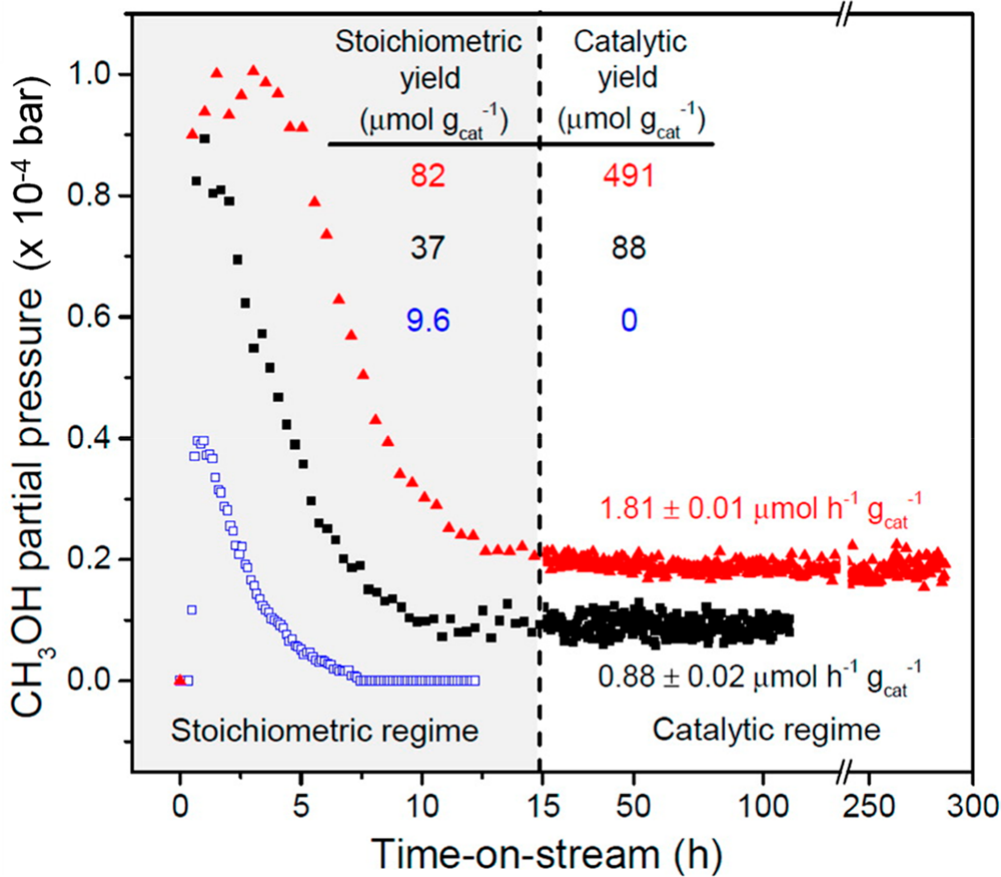
Methane oxidation over Cu-ZSM-5 after an initial dry methane oxidation (under 2400 mL h^–1^ g_cat_^–1^ of methane at 210 °C for 0.5 h). Methanol partial pressure with He (0.981 bar), H_2_O (0.032 bar), and O_2_ (0.000025 bar, 25 ppm) over (blue open squares) Cu-Na-ZSM-5 (Cu/Al = 0.37, Na/Al = 0.26). Methanol partial pressure with CH_4_ (0.981 bar), H_2_O (0.032 bar), and O_2_ (0.000025 bar, 25 ppm) over (solid squares) Cu-Na-ZSM-5 and (red solid triangles) Cu-H-ZSM-5 (Cu/Al = 0.31). Catalyst pretreatment: 5 h at 550 °C under oxygen flow, cooled to 210 °C under oxygen flow, and then purged under He for 0.5 h. Initial dry methane oxidation: 0.5 h under 2400 mL h^–1^ g_cat_^–1^ of methane at 210 °C. Reaction conditions: *T* = 210 °C, WHSV = 2400 mL h^–1^ g_cat_^–1^. Adapted with permission from ref (350). Copyright 2016 American Chemical Society.

The stepwise cyclic reaction of methane-to-methanol with oxidants (e.g., O_2_ or H_2_O) on Cu-zeolites brings commercial difficulties because of its stoichiometric property and the requirement of frequent temperature variations. Roman-Leshkov and co-workers reported that the active Cu sites in zeolites could directly catalyze methane conversion to methanol using O_2_ as the oxidant.^350^ As shown in Figure 29, stoichiometric and catalytic methanol production regimes were observed during the gas phase oxidation of methane over copper-exchanged zeolites with the MFI topology both in the sodium (Cu-Na-ZSM-5) and proton (Cu-H-ZSM-5) forms at 210 °C and atmospheric pressure.

As with the stepwise cyclic reaction, the catalysts were activated by an oxygen flow at 550 °C, cooled to reaction temperature, purged with He, then reacted with methane. A mixed gas (0.032 bar of water, 2.5 × 10^–5^ bar of oxygen and balance methane) was used to hydrolyze the surface-bound methoxy species to methanol. Under these conditions, Cu-Na-ZSM-5 (Cu/Al = 0.37, Na/Al = 0.26) and Cu-H-ZSM-5 (Cu/Al = 0.31) resulted in 37 μmol g^–1^ and 82 μmol g^–1^ of methanol, respectively. These values were higher than that obtained with Cu-Na-ZSM-5 using the extraction gas without use of methane (9.6 μmol g^–1^) or those reported by Lobo et al.^337^ (16 μmol g^–1^) using a wet inert gas (Table 2, entry 13) and Grouthaert et al.^335^ (8.2 μmol g^–1^) using an off-line solution extraction (Table 2, entry 7). This result may indicate that methane in the extraction gas could be also oxidized to methanol, causing an extra increase in methanol productivity. Importantly, a steady methanol production was observed by continually feeding the extracting gas (CH_4_, H_2_O, and O_2_) after all stoichiometrically produced methanol was desorbed (Figure 29, solid symbols). The methanol production as observed in a hundred-hours steady period with activity rates of 0.88 and 1.81 μmol h^–1^ g_cat_^–1^ over Cu-Na-ZSM-5 and Cu-H-ZSM-5, respectively. However, when methane was absent in the extraction gas mixture, methanol could not be continuously produced over Cu-Na-ZSM-5 catalyst (Figure 29A, open symbols), supporting the catalytic conversion of methane to methanol.

Lobo and co-workers^351^ reported the catalytic conversion of methane to methanol on SSZ-13 with N_2_O in place of O_2_, showing that using N_2_O resulted in higher methanol production than oxygen at temperatures of 200 and 300 °C (Table 3, entries 12–15). Further increasing reaction temperature to 450 °C led to lower production of methanol than oxygen, owing to the decomposition of N_2_O to O_2_ and N_2_ (Table 3, entries 16–17). To avoid N_2_O decomposition, the reaction was operated in the temperature range of 250–300 °C using a gas composition consisting of 30% CH_4_, 30% N_2_O, and 3% H_2_O (He balance). Steady state methanol production was achieved over Cu-SSZ-13 with a lifetime up to 23 h. However, under these conditions CH_3_OH, CO, and CO_2_ were observed as the main products, and the methanol selectivity was only between 20% and 27%. The data listed in Table 3 compares the catalytic activity of Cu-zeolites with different pore size for the direct catalytic conversion of methane to methanol. Cu-ZSM-5 showed the highest specific activity and STY among the medium- and large- pore zeolites. The small-pore zeolites SSZ-13 and SAPO-34 featured higher STY than Cu-ZSM-5 at 210 °C. Further increasing the reaction temperature to 260 °C achieved a much higher methanol STY yield on Cu-Na-SSZ-13 catalyst. These studies indicated that a crystalline, microporous structure with small pores was preferable for catalytic methane oxidation to methanol.

**Table 3:** Catalytic Methane Oxidation Rates at 1 bar over Various Cu-Zeolite Topologies

entry	zeolite	Si/Al ratio	Cu/Al ratio	WHSV (mL h^–1^ g^–1^)	activation *T* (°C)	CH_4_ reaction *T* (°C)	specific activity (μL h^–1^ g^–1^)	STY^c^(h^–1^ (×10^–3^))	methanol selectivity (%)	ref
1^a^	H-ZSM-5	11.5	0.31	2400	550	210	1.81	5.2		(350)
2^a^	H-β	12.5	0.30	2400	550	210	0.80	2.4		(350)
3^a^	MCM-41	12	0.74	2400	550	210	0.36	0.6		(350)
4^a^	H-ZSM-5	11.5	0.13	2400	550	210	0.84	6.0		(350)
5^a^	H-MOR	10	0.14	2400	550	210	0.84	4.6		(350)
6^a^	H-FER	10	0.12	2400	550	210	0.44	2.7		(350)
7^a^	Na-ZSM-5	11.5	0.37	2400	550	210	0.88	2.2	70.6	(350)
8^a^	Na–Y	5.1	0.45	2400	550	210	0.30	0.3		(350)
9^a^	Na-SAPO-34	0.3	0.02	2400	550	210	0.84	7.9		(350)
10^a^	Na-SSZ-13	15	0.50	2400	550	210	3.12	6.1		(350)
11^a^	Na-SSZ-13	15	0.50	2400	550	260	16.16	31.6		(350)
12^b^	SSZ-13	12	0.40		200 (N_2_O)	200	6.55	13.1		(351)
13^b^	SSZ-13	12	0.40		200	200	4.45	8.9		(351)
14^b^	SSZ-13	12	0.40		300 (N_2_O)	200	15.00	30.0		(351)
15^b^	SSZ-13	12	0.40		300	200	9.50	19.0		(351)
16^b^	SSZ-13	12	0.40		450 (N_2_O)	200	17.50	35.0		(351)
17^b^	SSZ-13	12	0.40		450	200	22.50	45.0		(351)

a Entries 1–11, catalyst pretreatment: activated at 550 °C under oxygen flow, cooled to 210 °C under oxygen flow, and then purged under He flow. Initial CH_4_ oxidation: 0.5 h under 2400 mL h^–1^ g^–1^ CH_4_ at 210 °C. Catalytic reaction conditions: He (0.981 bar), H_2_O (0.032 bar), and O_2_ (0.000025 bar, 25 ppm).

b Entries 12–17, catalyst pretreatment: activated at 200, 300, and 450 °C under oxygen or N_2_O and then cooled to 200 °C under He flow. Initial CH_4_ oxidation: 1 h under 7000 mL h^–1^ g^–1^ CH_4_ at 200 °C. Catalytic reaction conditions: The feed mixture containing CH_4_/N_2_O/He or CH_4_/O_2_/He was diverted through a water-containing saturator kept at 30 °C.

c Space time yield (STY) defined as mol_methanol_ mol_Cu_^–1^ h^–1^.

##### 4.3.3.2 Active Sites

###### Monocopper Species

**Figure 30:**
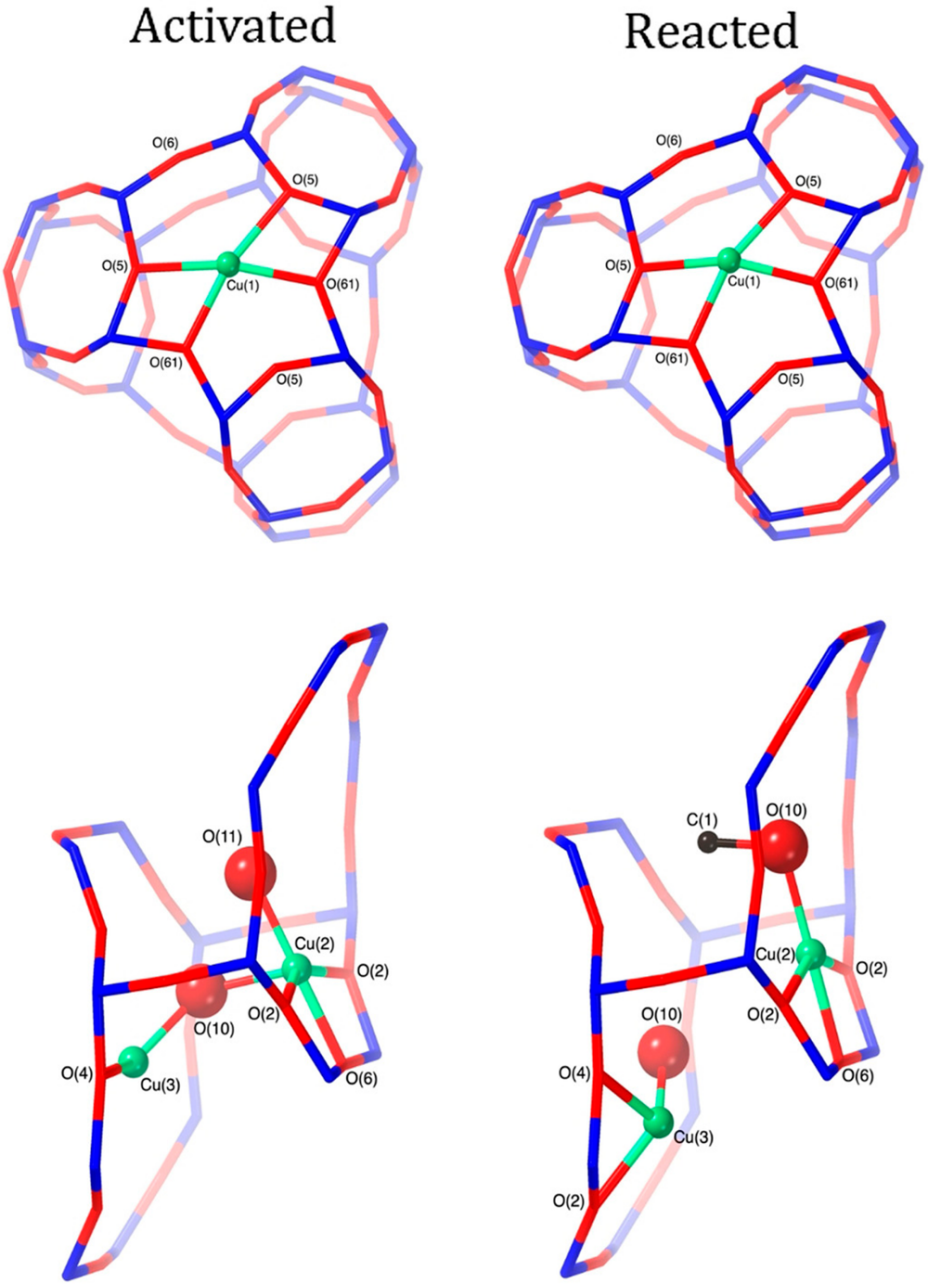
Structures of Cu-OMG zeolites, Cu/O_2_ (450 °C), and Cu/CH_4_ (200 °C). (top)\ Coordination of the Cu(1)^2+^ ions in a 6-ring of a gme cavity showing the difference between an occupied (front) and an unoccupied (back) 6-ring. (bottom) Coordination geometries of Cu(2) and Cu(3) before and after the introduction of CH_4_. Reproduced with permission from ref (356). Copyright 2021 John Wiley and Sons.

Different Cu sites can be formed on Cu-zeolites due to the structure of zeolite support, the method of introduction of the Cu species, and post-thermal treatment. The structure of Cu sites in zeolites significantly influences the activity in the methane to methanol reaction. To gain a deeper understanding of the reaction, significant effort has been devoted to the study of the Cu sites in Cu-zeolites by using various spectroscopic techniques.^335,336,352,353^ Monocopper species, for instance, Cu^+^, Cu^2+^, and [CuOH]^+^, were experimentally evidenced on Cu-zeolites by EPR, XAFS, and FTIR.^352,354^ These Cu species were, however, often considered to be precursors or spectators rather than active sites for methane conversion before the theoretical study by Kulkarni and co-workers.^355^ In their work, the low Al content CHA zeolite containing only one Cu atom on per Al site was used as the model system. At a temperature of 450 °C with 5% water partial pressure, equilibrium analysis, using Gibbs formation energies, predicted the Cu species to be comprised of 53% of 8-membered ring (8MR) Cu–H_2_O, 33% of 6-membered ring (6MR), MR–Cu, and 11% of 8-membered ring 8MR–Cu-OH. On the basis of Wulfers’s experimental observation that only 3–9% of total Cu species in Cu-CHA zeolites was involved in the oxidation of methane,^337^ the 8MR–Cu-OH species were suggested as the active sites and an energetically feasible path for the methane oxidation to methanol on this site was theoretically proposed. Recently, paired copper monomers [Cu-OH]^+^ were experimentally evidenced to be responsible for the methane-to-methanol conversion on Cu–OMG zeolite.^356^ Ex situ XAFS measurements and data analysis revealed three distinct locations of the Cu species on O_2_-activated OMG zeolites (Cu/O_2_/450 °C): Cu(1) in the 6-ring, and Cu(2) and Cu(3) in the gme-cavity 8-ring (Figure 30, top left). The Cu(1)^2+^ ion in the 6-ring was coordinated to four framework oxygen atoms (two at O(61) and two at O(5)); the Cu(2)^2+^ ion in the 8-ring was bonded to the framework oxygen atoms O(6), O(2), and a nonframework oxygen atom (O(11)); the Cu(3)^2+^ ion was connected with the framework O(4) and a nonframework O(10).

The nonframework O(10) has two possible positions: it could act as a bridge to the Cu(2) (Figure 30, bottom left) or point in the other direction down the narrow 8-ring channel; it was not associated with Cu(2). The Cu^2+^ ions at Cu(2) and Cu(3) are 3.45 Å apart, and they appear to exist as two proximal Cu[OH]^+^ monomers. Further experiments and analysis of the reacted sample (Cu/CH_4_/200 °C) showed that Cu(1) did not change after methane reaction (Figure 30, top right), whereas Cu(3) in the 8-ring moved from its position on the horizontal mirror plane to a location where it can interact with two framework oxygens, resulting in equivalent O(10) and O(11) positions. Electron density mapping showed that the C1 atom on methane was added on O(10) (Figure 30, bottom right). These extra-framework oxygen and carbon atoms probably constitute the adsorbed intermediate, methoxy [Cu^1+^-OCH_3_] species, which was observed by ^13^C NMR.^357^

###### Dicopper Species

**Figure 31:**
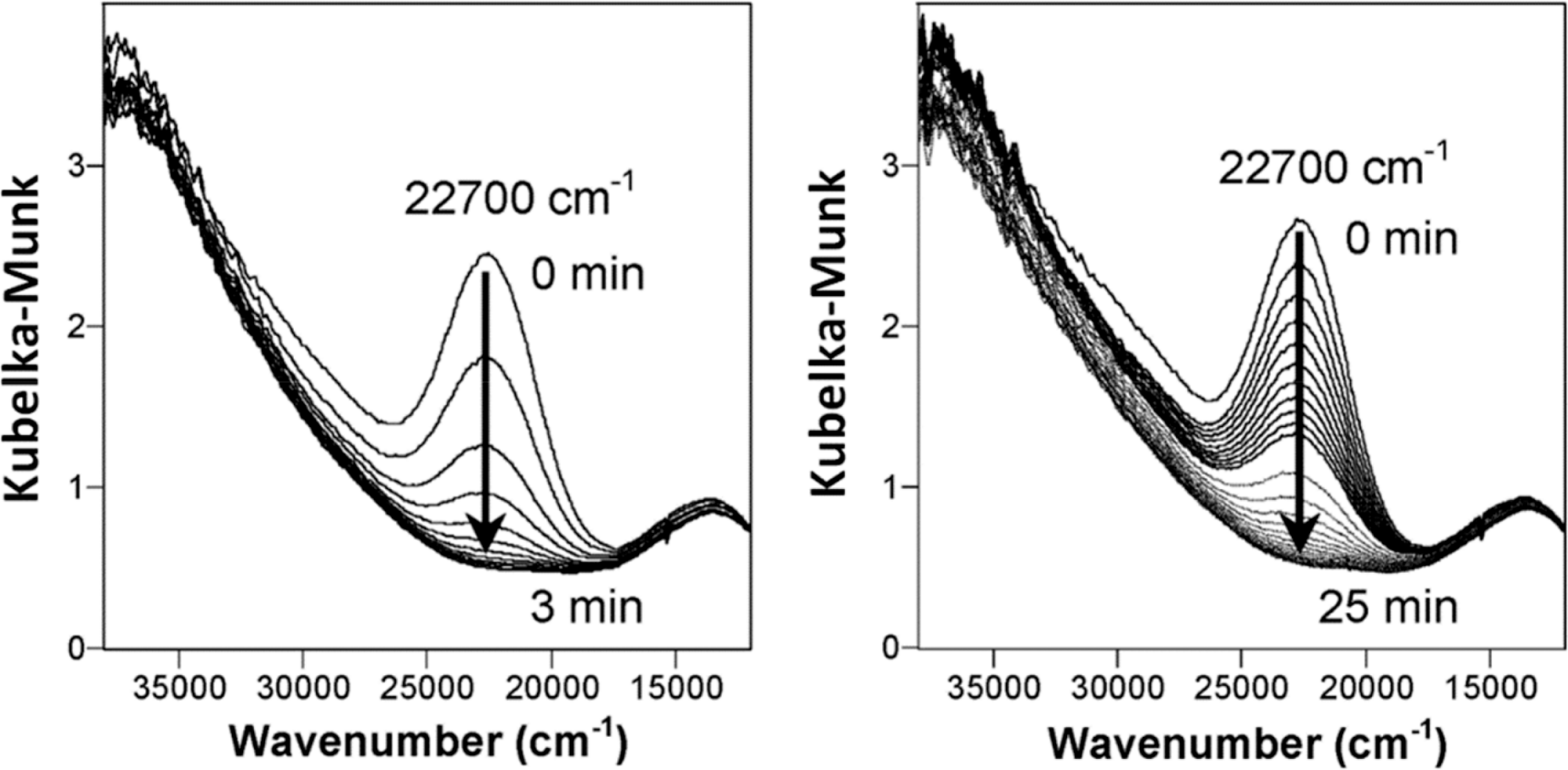
UV–vis spectra of O_2_-activated Cu-ZSM-5 during reaction with CH_4_ (5% in N_2_, 25 mL min^–1^) at 175 °C (left) and at 25 °C (right). Reproduced with permission from ref (335). Copyright 2005 American Chemical Society.

**Figure 32:**
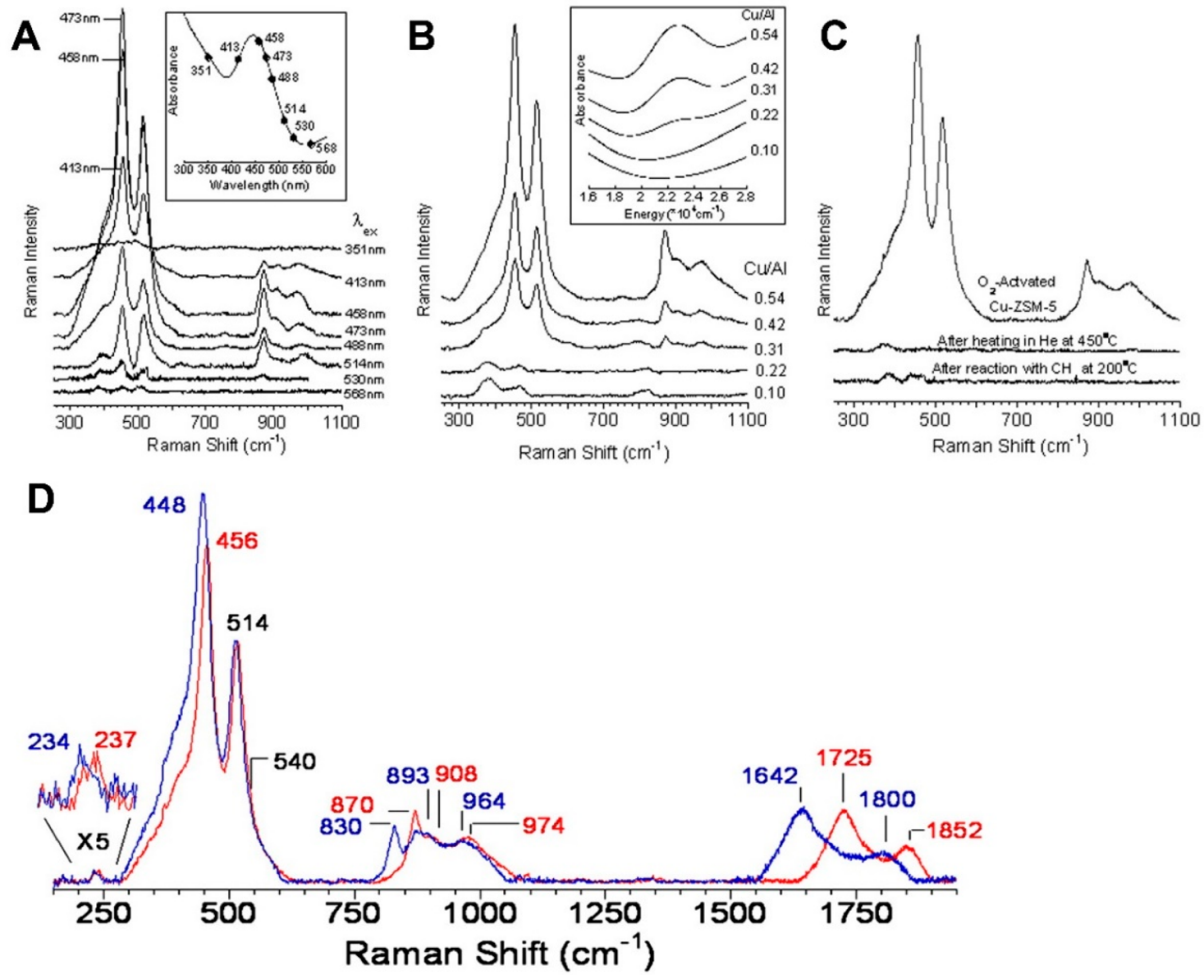
Resonance Raman (rR) spectra of O_2_-activated Cu-ZSM-5. (A) rR spectra of O_2_-activated Cu-ZSM-5 (Cu/Al = 0.54) collected at eight λ_ex_s from 351 to 568 nm with corresponding absorption spectrum inset. (B) rR spectra (λ_ex_ = 457.9 nm) of O_2_-activated Cu-ZSM-5 with varying Cu/Al ratios from 0.10 to 0.54 with corresponding absorption spectra inset. (C) rR spectra (λ_ex_ = 457.9 nm) of Cu-ZSM-5 (Cu/Al = 0.54) pretreated in O_2_ at 450 °C, recorded before and after heating in He at 723 K and after reaction with CH_4_ at 473 K. (D) rR spectra (λ_ex_ = 457.9 nm) of Cu-ZSM-5 activated by ^16^O_2_ (red) and ^18^O_2_ (blue). Reproduced with permission from ref (361). Copyright 2009 PNAS.

Inspired by the (μ-η^2^: η^2^-peroxo) dicopper structure in the natural protein hemocyanin,^358,359^ dicopper oxygen species have been considered as the most possible active sites on Cu-zeolites. Dicopper oxygen structures were identified with different atomic configuration and spectroscopic features (Table 4). In the report of Groothaert and co-workers,^335,353^ two UV–vis bands at 22700 (strong) and 30000 cm^–1^ (weak) were observed on Cu-ZSM-5 zeolite after it was treated with oxygen at high temperature; these bands fell within the UV–vis spectral range of *O*_bridge_ to Cu charge-transfer transitions on organic bis (μ-oxo) dicopper complexes.^360^ EXAFS analysis also showed the Cu–Cu and Cu–O distances in Cu-ZSM-5 zeolite were very similar to those of organic bis (μ-oxo) dicopper complexes.^353^ Therefore, a bis (μ-oxo) dicopper structure was tentatively assigned on the Cu-ZSM-5 zeolite. After the Cu-ZSM-5 was cooled to room temperature under a He flow, CH_4_ was introduced on to the sample at an elevated temperature. The band at 22700 cm^–1^ was observed to disappear (Figure 31), which did not occur when methane was absent, indicating that the reaction between methane and the assumed bis (μ-oxo) dicopper structure.^335^ To obtain an unambiguous identification of the active Cu species, resonance Raman (rR) spectroscopy was employed to determine the geometry of the active site of oxygen-activated Cu-zeolites.^361−364^ As shown in Figure 32A, the characteristic UV–vis absorption range (from 351 to 568 nm, corresponding to 17600 cm^–1^ to 28500 cm^–1^) of the oxygen-activated Cu-ZSM-5 sample produced resonance enhancement of Raman vibrations associated only with the active site for methanol oxidization.^361^ The rR vibrations intensity seesawed within the λ_ex_ range of 351 to 568 nm, indicating that these vibrations were in-resonance with this Cu–O electronic transition. The maximum resonance was observed using λ_ex_ = 457.9 nm, showing rR vibrations at 237, 456, and 870 cm^–1^ and a broad resonance at 974 cm^–1^. These vibrations gained intensity with increasing the Cu/Al ratio, the same tendency was found for the 22,700 cm^–1^ absorption band in UV–vis spectroscopy (Figure 32B), and they were not observed on the sample after being reacted with CH_4_ (at 200 °C) or heated under He at 350 °C (Figure 32C). These results confirmed that the rR vibrations observed were from the active Cu site. According to a previous report,^360^ bis (μ-oxo) dicopper complexes could be characterized by the isotope-sensitive vibrations at ∼600 cm^–1^. Isotope ^16/18^O_2_ rR experiments (Figure 32D) showed an isotope sensitive feature on Cu-ZSM-5 at 456 cm^–1^ [Δ(^18^O_2_) = 8 cm^–1^], 870 cm^–1^ [Δ(^18^O_2_) = 40 cm^–1^], and 1725 cm^–1^ [Δ(^18^O_2_) = 83 cm^–1^]. The absence of the featured rR vibration at ∼600 cm^–1^ precluded the existence of a bis (μ-oxo) dicopper species on O_2_-activated Cu-ZSM-5 zeolite, while a similar Raman profile was observed on a mono (μ-oxo) diferric complex.^365^ Therefore, a mono (μ-oxo) dicopper active site for the oxygen bridging two Cu structure was identified on Cu-ZSM-5 zeolite.

**Table 4:**
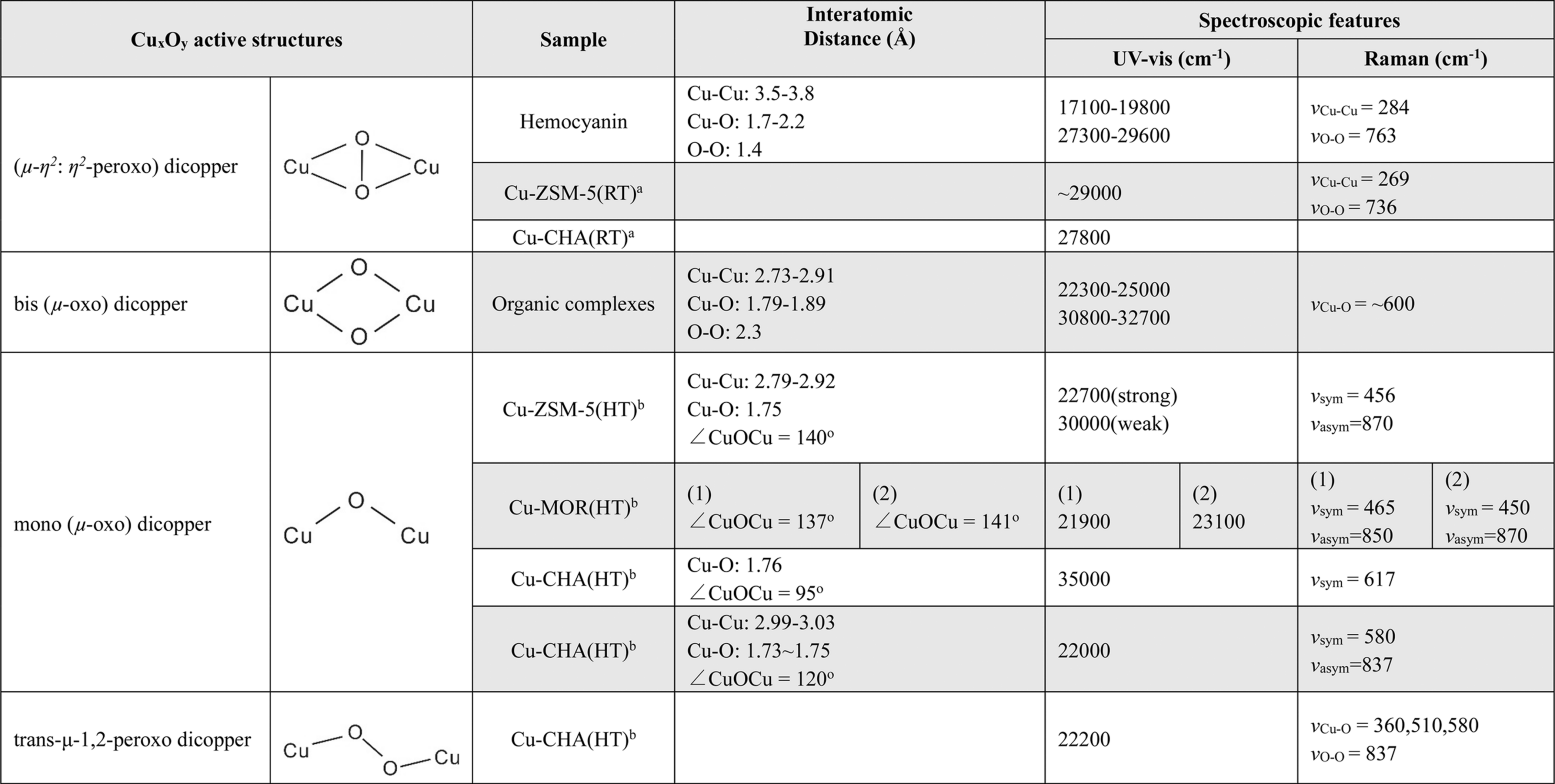
Proposed Dicopper Structures and Their Spectroscopic Features

a RT denotes room temperature treatment.

b HT denotes high temperature treatment.

**Figure 33:**
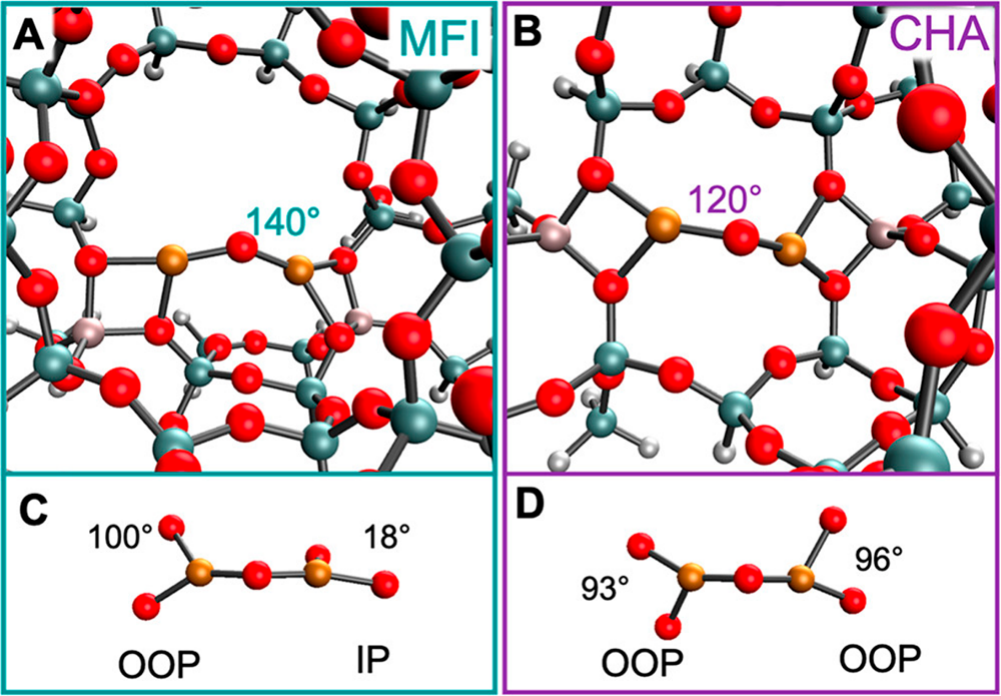
Mono (μ-oxo) dicopper species in the MFI (A) and CHA (B) lattices and their bidentate oxygen ligation in MFI (C) and CHA (D) assigning out-of-plane (OOP) and in-plane (IP) ligation with respect to the Cu–O–Cu plane. Reproduced with permission from ref (364). Copyright 2021 American Chemical Society.

Analogously, a mono (μ-oxo) dicopper active site was also identified on Cu–MOR^363^ and Cu–CHA^364,366^ zeolites after O activation at high temperature. There were two distinct mono (μ-oxo) dicopper active sites on Cu–MOR. They have very similar spectral features both on UV–vis and rR spectroscopy to those on Cu-ZSM-5 (Table 4), indicating that the mono (μ-oxo) dicopper species has a very similar structure on different zeolites. Normal coordinate analysis (NCA) was used to correlate the observed *v*_sym_ and *v*_asym_ vibrations and their isotope shifts to the bending angle (∠CuOCu) for the mono (μ-oxo) dicopper species. By fitting the observed *v*_sym_ and *v*_asym_ vibrations with ^16^O_2_ and ^18^O_2_ in NCA calculations, the mono (μ-oxo) dicopper species on ZSM-5 and MOR zeolites have ∠CuOCu of 140° and 137°(1)/141°(2), respectively (Table 4). Based on the NCA-calculated ∠CuOCu angle, the detailed structure of mono (μ-oxo) dicopper species and their locations on the zeolite lattice were further explained by DFT calculations. The mono (μ-oxo) dicopper site was stabilized in the 10-membered ring (MR) by an O-Al-O-(Si-O)_2_-O-Al-O unit on ZSM-5 (Figure 33A,C). Each Cu atom was bounded with two oxygen atoms on the Al sites. The bridge oxygen atom of the mono (μ-oxo) dicopper site pointed toward the middle of the 10-membered ring of ZSM-5. The location of the mono (μ-oxo) dicopper site on MOR were also figured out with the help of DFT calculations.^363^ Two O-Al-O-(Si-O)_2_-O-Al-O units at the side pocket of 12 MR and 8 MR channels have very similar Al–Al atom distance with that on ZSM-5 zeolite, indicating the ideal positions for the formation of two distinct mono (μ-oxo) dicopper sites. The formation of the mono (μ-oxo) dicopper active site on Cu–CHA zeolite was more complicated because of the small pore size of CHA zeolite (8 MR window). Two kinds of mono (μ-oxo) dicopper site were proposed (Table 4). One was located on an O-Al-O-(Si-O)_2_-O-Al-O unit in the 8MR with a small Cu–O–Cu angle (95°), showing a rR characteristic vibration at 617 cm^–1^ (not shown).^366^ The other was located on an O-Al-O-(Si-O)_3_-O-Al-O unit across the 8 MR with the bridge oxygen atom being out of the 8MR and forming a 120 °Cu-O-Cu angle (Figure 33B,D), which has rR characteristic vibrations at 580 (*v*_sym_) and 837 (*v*_asym_) cm^–1^.^361,364^

**Scheme 1:**
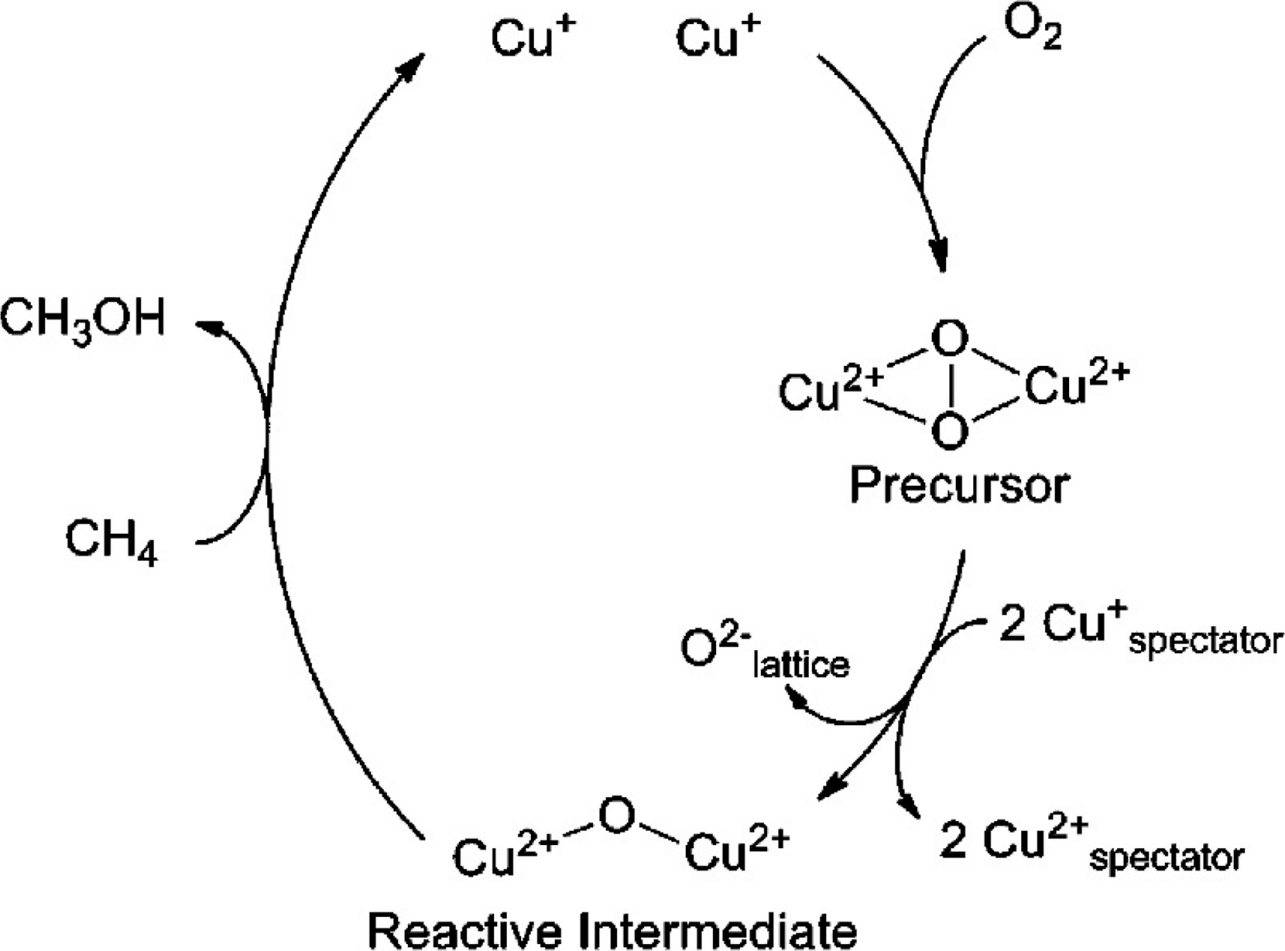
Formation of Mono (μ-oxo) Dicopper Reactive Intermediate on Cu-Zeolite Reproduced with permission from ref (367). Copyright 2010 American Chemical Society.

To understand the formation of dicopper oxygen species on zeolites, Smeets and co-workers examined the interaction between O_2_ and Cu-ZSM-5 at different temperature by UV–vis and Raman spectroscopy. An oxygen precursor species was identified prior to the formation of mono (μ-oxo) dicopper active site (Scheme 1).^367^ However, no oxygen precursor was found before the formation of mono (μ-oxo) dicopper active site when the He-activated Cu-ZSM-5 zeolite was exposed to N_2_O instead of O_2_,^335,361^ implying the presence of an O_2_-free formation route for the mono (μ-oxo) dicopper site. The results revealed a so-called “self-reduction” process of Cu species,^354,366,368^ in which H_2_O and OH could also offer oxygen atom for the formation of active Cu_*x*_O_*y*_ species such as the mono (μ-oxo) dicopper site.

DFT calculations coupled with microkinetic modeling have also been used by Engedahl and co-workers using the example of Cu-SSZ-13.^369^ They compare the reaction mechanism with and without water present. In both cases, the activation of methane to form ^•^CH_3_ radicals gives the highest barrier on the calculated potential energy surface. Water plays two roles in the calculations. First, it facilitates the migration of Cu cations through the zeolite lattice increasing the rate of formation of the active site dicopper structures. Second, the overall potential energy surface for methane to methanol is flattened in the presence of water, leading to lower barriers for the intermediate steps. The microkinetic model shows how this results in saturation of the catalyst by methanol which is stable up to 277 °C, effectively poisoning the low temperature reaction. When water is present the methanol desorption is enhanced, and active sites remain available even at low temperatures.

###### Tricopper and Larger Cu_*x*_O_*y*_ Species

**Figure 34:**
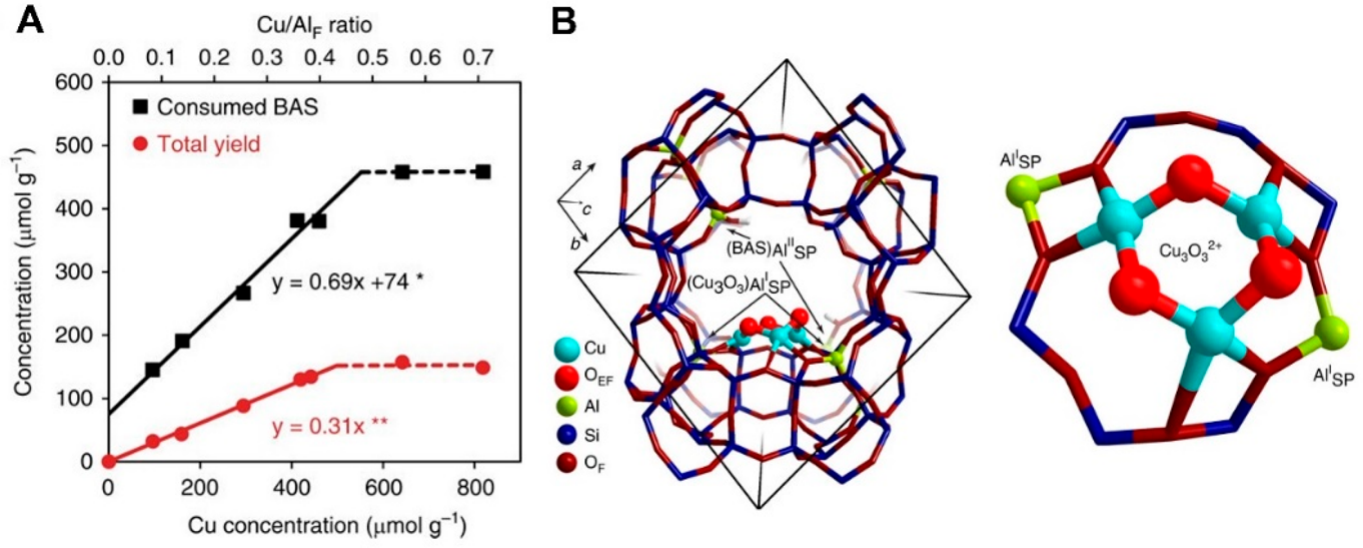
(A) BAS consumption and total methanol yield as a function of the Cu concentration for Cu–MOR with Si/Al = 11. *The slope of 0.69 indicates an exchange stoichiometry of 2/3, meaning that two H^+^ are substituted by three Cu^2+^. The offset of 74 μmol g^–1^ shows slight dealumination of framework Al (∼5%) during Cu exchange. **The slope of 0.31 indicates that three Cu atoms are involved in the oxidation of one methane molecule. (B) Optimal model structure stabilized by two anionic Si-O-Al sites at the entrance of the MOR side pocket. Reproduced with permission from ref (370). Copyright 2015 Springer Nature.

Grunder and co-workers prepared Cu–MOR using an improved ion-exchange method, in which the pH value was controlled at 5.7 to maximize the Cu-OH moieties and avoid further hydrolysis that could result in the precipitation of Cu(OH)_2_.^370,371^ The obtained catalysts showed a maximum methanol productivity of 160 μmol g^–1^, which was an order of magnitude higher than the reported value^335^ (13 μmol g^–1^, Table 2, entry 18) under the same reaction conditions. More interestingly, a quantitative analysis of the consumed Brønsted acid sites (BAS) and the Cu loading amount showed that two H^+^ were stoichiometrically substituted by three Cu^2+^ on Cu–MOR zeolite (Si/Al = 11). This stoichiometric substitution was found on a large series of Cu–MOR zeolites when the Cu/Al ratio was lower than 0.5 (Figure 34A, black). A linear dependency between the productivity of methanol and Cu concentration was also ascertained with a stoichiometry of three Cu cations being required to produce one methanol molecule (Figure 34A, red). This relationship between activity and Cu concentration was proved to be valid on Cu–MOR zeolites with various Si/Al ratios and Cu loading. The stoichiometry of the consumed BAS per Cu site together with the Cu/produced methanol ratio strongly suggested that only one kind of active site involving three Cu atoms anchored to two Al framework sites ([Cu_3_O_3_]^2+^) was formed on those Cu–MOR zeolites.

Molecular probe infrared (IR) techniques showed the Cu cations were perfectly exchanged on the BAS sites in the side pockets, indicating the [Cu_3_O_3_]^2+^ balanced two Si–O–Al sites near the pore mouth of MOR. X-ray absorption spectroscopy (XAS) analysis strongly supported the generation of the [Cu_3_(μ-O)_3_]^2+^ cluster on Cu–MOR. The trinuclear copper oxo-clusters were also predicted and identified on other zeolites.^372,373^ On a Cu-ZSM-5 catalyst, binuclear and trinuclear copper oxo-clusters could be preferentially stabilized depending on the conditions of catalyst activation.^372^ The possibility of formation of larger copper clusters containing more than three Cu were further theoretically evaluated,^374^ indicating that the stability of the system generally increases with the cluster size. The tetra- and pentamer clusters are more stable than dimers and trimers due to the additional stabilizing effect of the multiple Cu–O linkages on the overall cluster integrity. More complex situations were found by the comparison of the copper species on Cu–MOR and Cu–MAZ zeolites. Multiple copper species coexistent on Cu-zeolite catalysts, including the Cu^2+^ cations coordinated with framework oxygen, mono (μ-oxo) dicopper, bis (μ-oxo) dicopper, tricopper species, and Cu–OH^+^. Their structure, formation, composition, and stabilization were strongly influenced by the type of zeolite and the Si/Al ratio.^373^ The oxidation state, proximity, and mobility of the Cu sites upon different redox treatments also influence the structure of Cu clusters.^375^

##### 4.3.3.3 Mechanism

###### Direct Dissociation of Methane by Mono Copper–Oxygen Species

**Scheme 2:**
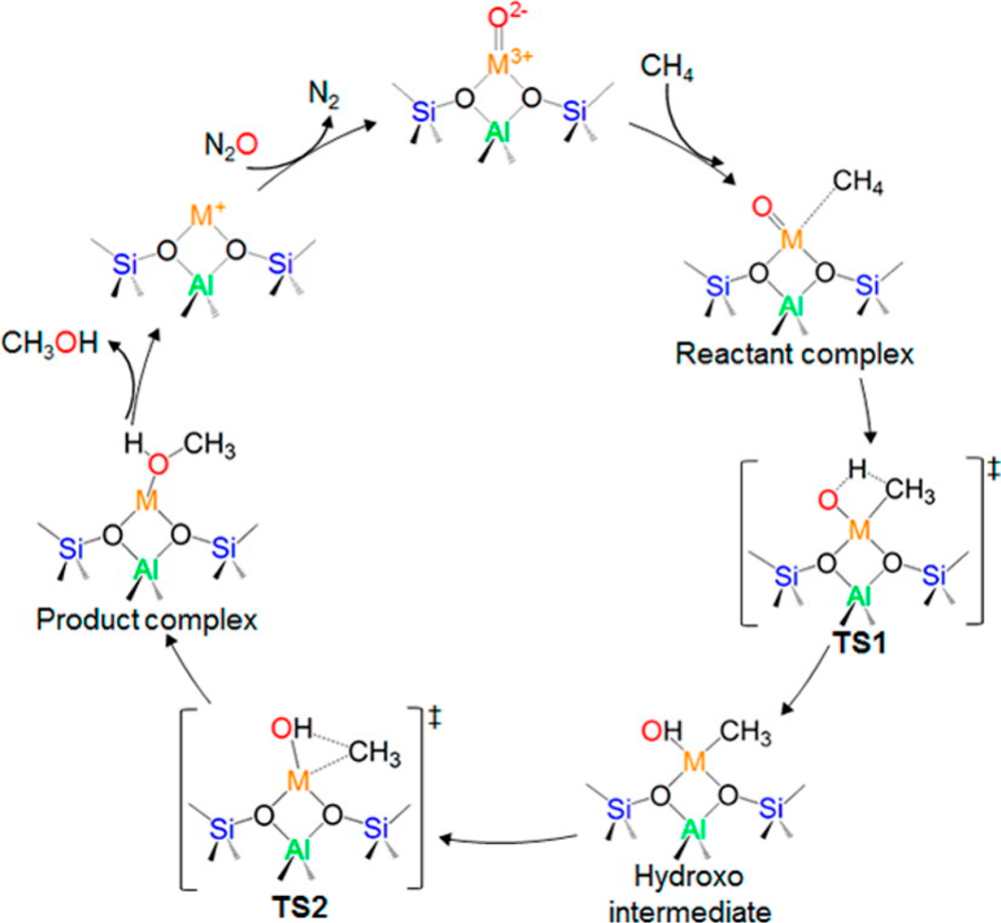
Possible Catalytic Cycle for the Methane to Methanol Conversion by MO^+^-ZSM-5 Reproduced with permission from ref (379). Copyright 2016 American Chemical Society.

The pioneering work of Panov and co-workers indicated that the surface α-oxygen on N_2_O activated Fe-ZSM-5 is able to oxidize methane to methanol with relatively high activity and selectivity (section 3.2).^376^ The methane activation on Cu-zeolites reported by Schoonheydt and co-workers^335^ stimulated much research effort on the understanding of methane oxidation on copper active sites.^377^ DFT predictions showed that in the presence of water, the dicopper species on Cu-zeolites can transform to a mono CuO species, which is capable of activating methane with a lower energy barrier.^378^ Mahyuddin and co-workers compared the direct oxidation of methane on mono CuO and other metal–oxygen (MO) sites located on a ZSM-5 framework using the well-established catalytic cycle for methane oxidation.^379^ As shown in Scheme 2, the production of methanol from methane includes two-step conversion via two transition states (TSs): MO^+^-ZSM-5 + CH_4_ (dissociation limit) → [MO(CH_4_)]^+^-ZSM-5 (reactant complex) → TS1 → [HOM-CH_3_]^+^-ZSM-5 (hydroxo intermediate) → TS2 → [M(CH_3_OH)]^+^-ZSM-5 (product complex) → M^+^-ZSM-5 + CH_3_OH (final complex).^380,381^

**Scheme 3:**
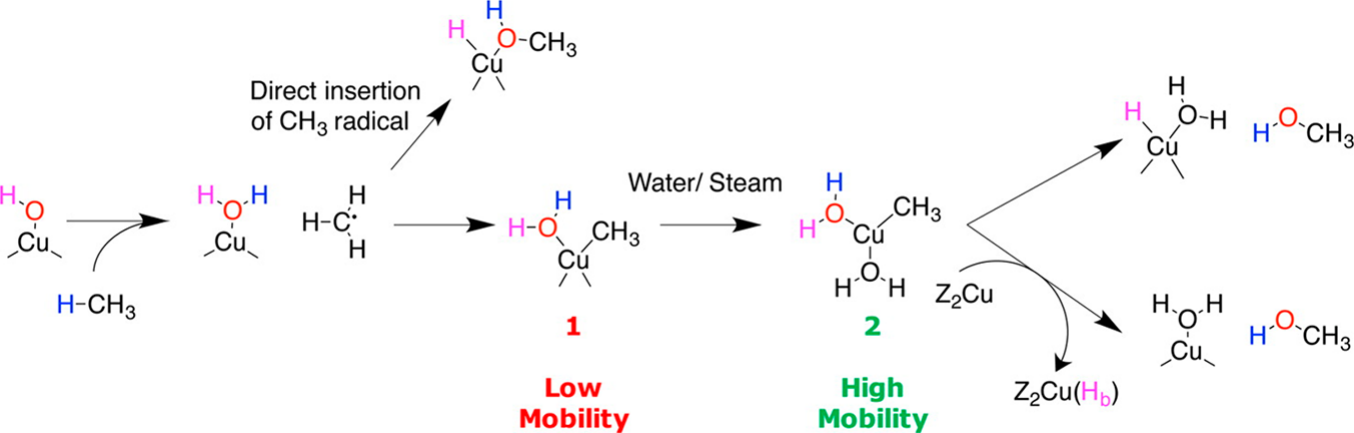
Reaction Scheme for Partial Methane Oxidation to Methanol for 8MR-[CuOH]^+^ Site in Cu–CHA Zeolite Reproduced with permission from ref (355). Copyright 2016 American Chemical Society.

On the basis of the calculated activation energies, together with the confinement effect of ZSM-5 on the activity, the dissociation of the C–H bond in methane to the hydroxo intermediate was predicted in the order CoO^+^-ZSM-5 < NiO^+^-ZSM-5 < FeO^+^-ZSM-5 < CuO^+^-ZSM-5, while the selectivity of methanol production from the intermediate was estimated to increase in the order FeO^+^-ZSM-5 < CoO^+^-ZSM-5 < NiO^+^-ZSM-5 < CuO^+^-ZSM-5. The mono CuO^+^ sites were predicted to show advantages both in the high activity for methane C–H bond activation and high selectivity for methanol production. These results show that the methane-to-methanol reaction on mono metal–oxygen species was highly dependent on the metal speciation. Similar methane C–H bond dissociation was also predicted on the [Cu-OH]^+^ site in small pore CHA zeolite. The insertion of CH_3_ onto the oxygen atom of [Cu-OH]^+^ site was energetically unfeasible. In contrast, the addition of CH_3_ on to the Cu atom to form a [Cu-H_2_O-CH_3_]^+^ species attached to the CHA framework (denoted as 1 in Scheme 3) was easier and has low mobility. When water or steam was introduced into this system, a freely diffusible [Cu-2(H_2_O)-CH_3_]^+^ species (denoted as 2 in Scheme 3) was released from the framework. Therefore, two methanol production routes were proposed from the solvated [Cu-2(H_2_O)-CH_3_]^+^ species, one being the self-decomposition to methane and the other being the migration of one H atom from the solvated species to the zeolite framework, resulting in the formation of a new Brønsted acid site. On the basis of the energy profiles, the latter process was significantly more favorable.^355^ Although the above theoretical work showed several feasible pathways for methane activation on mono copper–oxygen sites, there are still some uncertainties due to the experimental difficulty of in situ observation and capturing the reaction intermediate.

###### Stepwise Reduction–Oxidation Mechanism on Cu_*x*_O_*y*_ Clusters

**Scheme 4:**
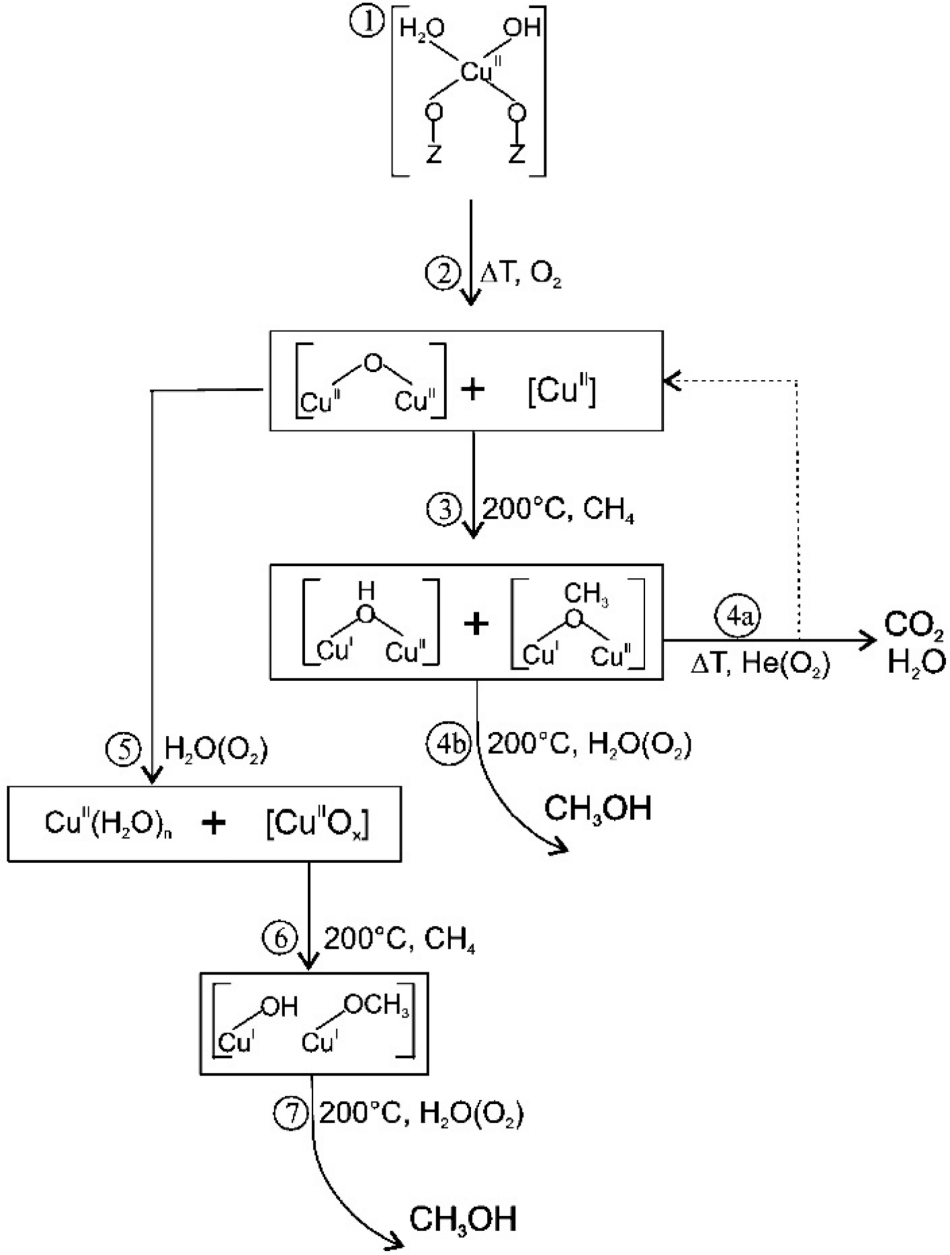
Proposed Scheme of Structural Changes of the Active Cu Species in Cu-Zeolite Reproduced with permission from ref (383). Copyright 2014 American Chemical Society.

The rR spectroscopy and DFT calculation have shown that mono(μ-oxo) dicopper, trinuclear, and larger copper oxygen clusters can act as the active site on Cu-zeolites depending on the type of zeolite framework and catalyst preparation and activation conditions.^335,361,370,374^ The DFT calculations by Woertink and co-workers suggested a radical rebound mechanism for the oxidation of methane on mono(μ-oxo) dicopper site.^361,382^ This kind of active site can abstract a H atom from CH_4_, resulting in the formation of a [Cu-OH-Cu]^2+^ intermediate and a CH_3_ radical with an activation energy of 18.5 kcal mol^–1^. The delocalized-radical structure of the [Cu-OH-Cu]^2+^ intermediate, together with its strong O–H bond, promoted the reaction. In the following step, the rebound of the hydroxyl radical to couple with the CH_3_ radical produced methanol, leaving two Cu^I^ on the zeolite framework. Alayon and co-workers studied the reduction–oxidation of Cu atoms in high temperature-activated Cu-MOR by in situ XAS spectroscopy, showing more details on the valence state change of the copper atoms caused by methane activation (Scheme 4).^383^ High-temperature dehydration and O_2_ activation transformed the Cu^2+^ cations into a mono(μ-oxo) dicopper active site (step 1–2, Scheme 4). In the reaction with methane, almost half of the Cu^II^ sites were reduced to Cu^I^ and a small fraction of Cu^II^ was found to coordinate with water or OH species (step 3, Scheme 4).

The abstraction of a H atom from CH_4_ by two mono(μ-oxo) dicopper active sites is an energetically favorable reaction, producing two stable [Cu^I^–OCH_3_–Cu^II^] and [Cu^I^–OH–Cu^II^] species (step 3 Scheme 4). Introducing water/steam allowed desorption of methanol from the [Cu^I^–OCH_3_–Cu^II^] intermediates (step 4b, Scheme 4). The water-stable Cu^II^ oxide species were also able to oxidize methane to methanol (step 5–7, Scheme 4), whereas the formation of water-stable Cu^II^ oxide species did not require such a high temperature as that for mono(μ-oxo) dicopper species. This indicated the existence of reduction–oxidation process in the low temperature methane to methanol oxidation.

Knorpp and co-workers compared the redox Cu species in isothermal low-temperature and conventional high-temperature methane oxidation on Cu-zeolite.^384^ Owing to the presence of moisture and the relatively low activation and reaction temperature in the isothermal procedure, the Cu species were not able to form the same active species as the dimer or oligomeric copper in the high-temperature activation procedure. Therefore, the water-stablized Cu species was proposed to be the dominant active site for methane oxidation to methanol. They analyzed the amount of Cu^I^ formation using XANES and the methanol yield, showing that the copper-to-methanol ratio converged to 2 mol_Cu(I)_ mol_methanol_^–1^. For comparison, the high-temperature procedure exhibited a similar ratio of Cu^I^ per mol of methanol. This indicated the same two-electron redox mechanism involving a Cu^I^ and Cu^II^ couple in both the isothermal low-temperature and conventional high-temperature procedures,^385^ although the actives sites were different. The exact structure of the water-stable Cu sites was not determined, however; the methane oxidation pathway was expected to be similar to that on the mono(μ-oxo)dicopper site.^383^ Recently, the water-stable Cu species was theoretically identified by Göltl and co-workers using DFT calculations.^386^ The result showed that two hydroxylated dimers, Cu_2_O_2_H_2_ and Cu_2_OH were thermodynamically preferred for the oxidation of methane on Cu-SSZ-13 zeolite. When these hydroxylated dimers were exposed to methane, site-bound methanol molecules were formed and subsequently released by the increase of water vapor pressure.

**Scheme 5:**
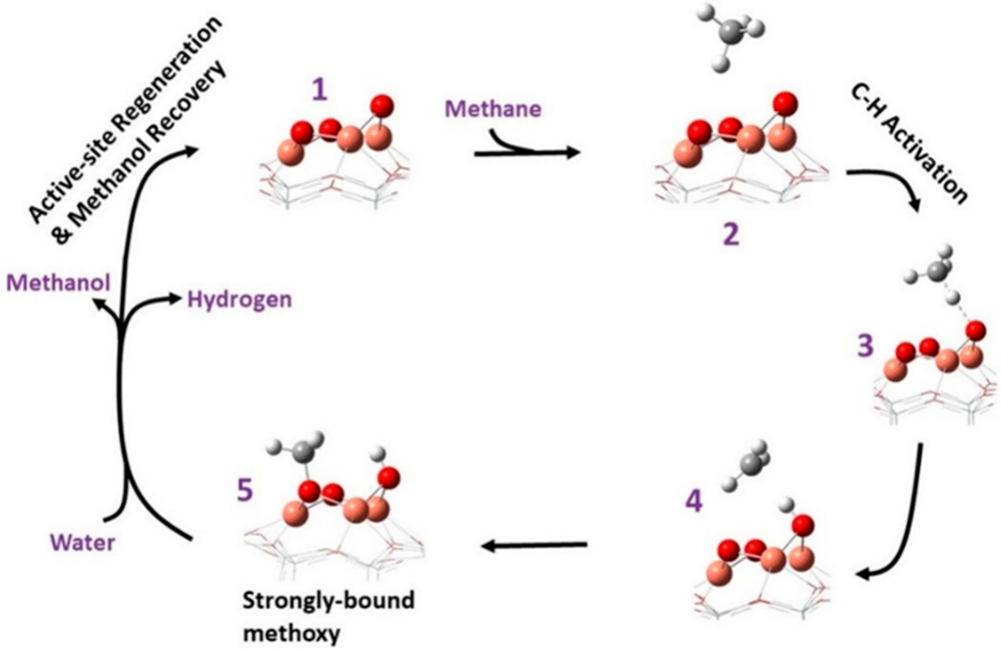
Activation of Methane C–H Bond to Methanol by [Cu_3_O_3_]^2+^ Active Site, with Steam-Facilitated Extraction of Products and Regeneration of the Active Copper Oxo Cluster Reproduced with permission from ref (388). Copyright 2021 John Wiley and Sons.

Most of the reports discuss the redox mechanism for methane oxidation on dimer or paired Cu sites with coupled Cu^I^ and Cu^II^.^385^ For the trinuclear and larger copper–oxygen clusters the redox couple was analogously proposed as Cu^II^ and Cu^III^.^370,387^ The investigation of the reduction–oxidation on larger Cu clusters, however, was complicated because of the difficulty in spectral distinction of Cu^III^ from Cu^I^ and Cu^II^ atoms. DFT calculations have often been employed to understand the methane to methanol oxidation on the larger copper–oxygen clusters.^370,387,388^ The analysis of the electronic structure of the [Cu_3_(μ-O)_3_]^2+^ site suggested that the Cu was predominantly present in the Cu^II^ state with a minor contribution of Cu^III^, resulting in radical character on the O(1) and O(3) sites. The C–H activation barrier (electronic energies) analysis showed that the O(1) site is more active than the O(2) and O(3) sites. Methane was activated through a homolytic C–H bond cleavage on the O1 site, producing gas-phase CH_3_ radical and the OH group bonded to the trimer active site; the CH_3_ radical then rebounds to the active site, forming a methoxy species. Finally, CH_3_OH and H_2_ are released by the addition of water (Scheme 5). Moreover, the presence of multiple Cu–O linkages on the larger Cu-oxy clusters endowed them with a more favorable electronic structure and better stabilizing effect on the OH group and CH_3_ fragment, generating higher activity in the oxidation of methane.^372,374^

###### Effect of the Water in the Methane to Methanol Reaction

Being the most ideal extractor, water is employed to release methanol from Cu-zeolite in methane oxidation reactions achieved by stepwise^335,343^ and catalytically continuous manners.^350^ Water also has an important role in modulating the activity of dicopper [Cu–O–Cu]^2+^ sites in zeolites.^378^ DFT calculations showed that the introduction of one water molecular on to the [Cu–O–Cu]^2+^ site finally resulted in the formation of oxygen-containing radical intermediates, HO–Cu–O–Cu–OH and HO–Cu–OH–CuO. Energy analysis indicated that those intermediates can effectively catalyze the homolytic cleavage of the methane C–H bond. Under specific conditions the oxygen activation was not necessary and anaerobic oxidation of methane was possible on Cu-zeolite, in which water molecules acted as an oxygen source to oxidize methane.^349,389,390^ The role of water was 2-fold: first a contribution of the oxygen atom for the two-electron reduction–oxidation, and second the desorption of methanol. In the comparison of the anaerobic and aerobic continuous partial methane oxidation on Cu-zeolites, it was found that the solo water oxidant has an advantage over the O_2_ and water mixed oxidant in suppressing overoxidation, resulting in the highly selective production of methanol.^389,390^ Additionally, it was also found that water has a promoting effect on hydrogen release in anaerobic oxidation of methane to methanol over Cu–MOR.^391^ Recently, it was demonstrated that H_2_O molecules can participate in continuous methane oxidation on Cu–BEA through a proton transfer pathway, in which a high-speed proton transfer between the generated ^•^CH_3_ and ^•^OH was mediated by H_2_O molecules. As a result, the methane oxidation reaction performance, including the selectivity and productivity of methanol and the stability of catalyst, was significantly boosted compared with the reaction without H_2_O.^392^ Combining a D_2_O isotopic tracer technique and ab initio molecular dynamics (AIMD) simulation, the authors unravelled the proton transfer mechanism for methane oxidation to methanol over the dicopper [Cu–O–Cu]^2+^ site.

## 5 Conclusions and Outlook

Research into designing catalysts capable of the selective oxidation of methane to methanol has represented a grand scientific challenge for over a century. In the last few decades, a new approach has appeared approximately every 10 years, and this engenders a new surge of research activity. So as a research topic there is always interest and something new to consider. Of course, with the current global interest in climate change and the need to stop the use of fossil sources of carbon, it could be thought that interest in the reaction would start to wane. However, this is not the case, and there is now interest in bioderived methane which can be a feedstock of the future. However, even though there has been sustained research interest, it can be argued that despite an enormous amount of excellent scientific work, currently the known methods for selective oxidation of methane to methanol fall substantially short of the performance required at larger scale. In this sense, formidable commercial and environmental performance targets must be met, at least a high selectivity of ca. 75% at meaningful once-through conversions (i.e., ≥5%) will be needed. Despite this, the opportunity space for direct conversion of methane has evolved over the past 40 years from exploitation of remote “stranded” or “associated” natural gas to competition with an increasing number of viable, near-term alternatives for capturing and valorizing methane emissions and resources, including gas that is currently flared and biomethane. This review covers diverse processes and in particular focuses on the contribution that heterogeneous catalysis has made on this important chemical reaction. However, the low reactivity of methane under conditions that facilitate isolation or recovery of desirable products such as methanol remains a distinct challenge to both the catalysis and more broadly to the scientific community.

Due to the high C–H bond strength in CH_4_, high temperature catalytic approaches were initially pursued, especially using metal oxide catalysts. However, it is evident that high temperature approaches have significant contributions from homogeneous gas phase reactions. As such, the control that could potentially be offered by surface catalyzed reactions is diminished. Subsequently, new low temperature methodology would appear to be a more promising approach. Nature offers us clues for more successful methodologies, as at lower temperatures the potential to promote selectivity to oxygenated products, like methanol, can be achieved and a reduction of over oxidation to carbon oxides more readily realized. At low temperatures, aqueous environments appear favorable for selectivity to oxygenated products through a combination of solvation effects (on transition states) and possible influence on radical abundances and kinetics.

Using H_2_O_2_ as an oxidant can lower the reaction temperature significantly compared to those that are required by other chemocatalytic approaches. Practically speaking, the cost of ex situ generated H_2_O_2_ prohibits the use of the preformed oxidant. Alternatively, in situ generation of H_2_O_2_ from the elements would significantly lower production costs. Great strides in catalyst design have led to near-total catalytic selectivity toward direct H_2_O_2_ synthesis under optimized conditions.^243,393^ There are a growing number of reports that have demonstrated that in situ H_2_O_2_ synthesis is possible under conditions far more detrimental to H_2_O_2_ stability than those typically utilized for methane oxidation and that near total H_2_ utilization (another major hurdle that must be overcome prior to industrial application) can be achieved. Indeed, such approaches have been utilized for methane oxidation leading to significant advances in recent years. An increased focus should be placed on the design of catalysts that promote the generation and release of reactive oxygen species (^•^OOH and ^•^OH), through combination of H_2_ and O_2_, rather than relying on a tandem approach where H_2_O_2_ is synthesized and subsequently cleaved to form radical species. While there is still great promise in the H_2_O_2_ driven route to methane oxidation, as yet both the need for extended contact times and limited catalytic activities has hampered development, and future studies would benefit from a focus on improving methane conversion rates and shifting toward continuous flow systems.

Cu-containing zeolites have been demonstrated to be one of the most promising catalysts for partial oxidation of methane to methanol. Both stoichiometric and catalytic reactions have been achieved. Various factors such as the zeolite framework type, morphology, and chemical composition of zeolite, Cu species, and the reaction conditions (e.g., temperature and pressure) significantly influence the activation of methane and methanol formation. Therefore, despite notable progress, the Cu-zeolites catalyzed methane oxidation reaction is far away from the practical application regarding the low methane turn over frequency and methanol productivity.

Although the direct C–H bond dissociation and stepwise reduction–oxidation routes have been often considered, the methane oxidation mechanism on Cu-zeolites remains elusive. However, the role of water in methane oxidation merits further research as water acts not only as solvent to release methanol from the zeolite but also as a promoter to modulate the activity of Cu active sites. Importantly, the unusual function of water as the oxidant for methane conversion provides a promising route for the selective formation of methanol by avoiding the notorious overoxidation by using O_2_ or N_2_O oxidant. The understanding of the structures, interactions and dynamics of water molecules in zeolite channels would greatly benefit the efficacy of Cu and other metal modified zeolites on taking advantage of water in methane oxidation toward high methanol productivity under mild conditions.

However, on a larger scale, dilute aqueous products are not attractive, and substantially higher product concentrations are required. Low temperature approaches using zeolites with metal nanoparticles work, but the product concentrations are vanishingly small. Water is important in these systems, but the products are then dilute and require challenging separation and treatment. Different metals operate with different final mechanistic steps (i.e., with Cu–water is the source of O in methanol, with Au, the O comes from O_2_). In the presence of a coreductant (CO, H_2_), the reaction is much faster, but the products are still too dilute. Therefore, what is now needed is a wholly gas phase process operating at higher temperatures using steam. In some respects, this has been attempted using N_2_O as an oxidant and iron-containing zeolites (e.g., Fe-ZSM-5), where steam is required to ensure methanol is removed before transforming into undesirable side products via the hydrogen–carbon pool mechanism within the zeolite and fouling the catalyst. Excellent work has been devoted to understanding the active center (i.e., α-oxygen), both spectroscopically and through simulation. However, the methane conversion per pass remains low and the methanol concentration in the effluent needs to be significantly improved.

Although homogeneous gas phase radical chemistry can produce comparatively high yields of oxygenated products, there seems limited opportunity for further improvement. However, a potential gas phase contribution to heterogeneous catalysis at higher temperatures and pressures should always be considered and managed. Furthermore, driving radical chemistry in nonthermal plasmas can produce high oxygenate yields. However, in addition to practical scale-up challenges, the electrical power demand is currently too high in comparison to alternatives such as electrified reforming. To widen the applicability of any technology in this field, chemical oxidants should be either molecular oxygen or readily regenerable from O_2_. Efficient use of oxygen as well as any low-cost cofactors (for example CO) is also important. In the absence of cofactors, selective formation of methanol requires incorporation of both oxygens from O_2_ into the product, whereas more oxidized products such as formaldehyde or acetic acid require only 50%. Consequently, research strategies need to protect desired products from further oxidation or target more oxidation resistant end products such as acetic acid.

The increasing demand of methanol underline the need to develop effective strategies to exploit the abundance of fossil-derived methane and the emergence of biogas. The later can be a key driver in the transition to net-zero carbon processes, where heterogeneous catalysts can play a crucial role. Clearly, the demanding performance targets discussed in this review impede adoption of many of the technologies that have been thus far explored. Although there are promising strategies discussed here that can be developed further with continued catalyst design, advanced material characterization such as operando studies and supporting computational approaches, greater effort is required. The outlook for impactful research in this field remains encouraging and novel advances are applicable to other processes where C–H activation is required. We therefore remain optimistic that this long-term grand challenge of catalysis can and will be solved through a combination of innovative catalysis and engineering approaches, especially with the advent of sustainably sourced methane.
